# Noncanonical HPV carcinogenesis drives radiosensitization of head and neck tumors

**DOI:** 10.1073/pnas.2216532120

**Published:** 2023-07-31

**Authors:** Travis P. Schrank, Aditi Kothari, William H. Weir, Wesley H. Stepp, Hina Rehmani, Xinyi Liu, Xiaowei Wang, Andrew Sewell, Xue Li, Jason Tasoulas, Sulgi Kim, Gray Yarbrough, Yue Xie, Yael Flamand, Shanthi Marur, Michele C. Hayward, Di Wu, Barbara Burtness, Karen S. Anderson, Albert S. Baldwin, Wendell G. Yarbrough, Natalia Issaeva

**Affiliations:** ^a^Department of Otolaryngology/Head and Neck Surgery, The University of North Carolina at Chapel Hill, Chapel Hill, NC 27599; ^b^Lineberger Cancer Center, The University of North Carolina at Chapel Hill, Chapel Hill, NC 27599; ^c^Department of Pharmacology and Regenerative Medicine, University of Illinois at Chicago, Chicago, IL 60612; ^d^University of Illinois Cancer Center, Chicago, IL 60612; ^e^Dana Farber Cancer Institute Eastern Cooperative Oncology Group and the American College of Radiology Imaging Network Biostatistics Center, Boston, MA 02109; ^f^Johns Hopkins Univ/Sidney Kimmel Cancer Center, Baltimore, MD 21231; ^g^Department of Biostatistics, The University of North Carolina at Chapel Hill, Chapel Hill, NC 27599; ^h^Division of Oral and Craniofacial Health Sciences, Adams School of Dentistry, The University of North Carolina School of Medicine at Chapel Hill, Chapel Hill, NC 27599; ^i^Department of Internal Medicine and Yale Cancer Center, New Haven, CT 06510; ^j^Department of Pharmacology, Yale University School of Medicine, New Haven, CT 06520; ^k^Department of Molecular Biophysics and Biochemistry, Yale University, New Haven, CT 06520; ^l^Department of Pathology and Lab Medicine, The University of North Carolina at Chapel Hill, Chapel Hill, NC 27599

**Keywords:** HPV, head and neck cancer, radiosensitization, tumor microenvironment, patient outcome

## Abstract

We analyzed transcriptional data from 104 HPV+ (Human papillomavirus) HNSCC (head and neck squamous cell carcinoma) tumors together with two publicly available sources to identify highly robust transcriptional programs (modules) which could be detected consistently despite heterogeneous sequencing and quantification methodologies. Among 22 modules identified, we found a single module that naturally subclassifies HPV+ HNSCC tumors based on a bimodal pattern of gene expression, clusters all atypical features of HPV+ HNSCC biology into a single subclass, and predicts patient outcome in four independent cohorts. The subclass-defining gene set was strongly correlated with Nuclear factor kappa B (NF-κB) target expression. Tumors with high expression of this NF-κB module were rarely associated with activating PIK3CA alterations or viral integration, and also expressed higher levels of HPHPV E2 and had decreased APOBEC mutagenesis. Alternatively, they harbored inactivating alterations of key regulators of NF-κB, TNF receptor associated factor 3 (TRAF3), and cylindromatosis (CYLD), as well as retinoblastoma protein (RB1). HPV+ HNSCC cells in culture with experimental depletion of TRAF3 or CYLD displayed increased expression of the subclass-defining genes, as well as robust radio-sensitization, thus recapitulating both the tumor transcriptional state and improved treatment response observed in patient data. Across all gene sets investigated, methylation to expression correlations were the strongest for the subclass-defining, NF-κB-related genes. Increased tumor-infiltrating CD4+ T cells and increased Estrogen receptors alpha (ERα) expression were identified in NF-κB active tumors. Based on the relatively high rates of cure in HPV+ HNSCC, deintensification of therapy to reduce treatment-related morbidity is being studied at many institutions. Tumor subclassification based on oncogenic subtypes may help guide the selection of therapeutic intensity or modality for patients with HPV+ HNSCC.

## Significance

Human papillomavirus–associated (HPV+) head and neck squamous cell carcinoma (HNSCC) is now the most common HPV-associated cancer with increasing incidence. HPV-mediated oncogenesis is generally thought to rely on the integration of the viral DNA into the host genome, loss of HPV early gene 2 (HPV E2) expression, activation of Phosphatidylinositol-4,5-Bisphosphate 3-Kinase Catalytic Subunit Alpha (PIK3CA), and apolipoprotein B mRNA editing catalytic polypeptide (APOBEC)-mediated mutagenesis. We report the identification of a subclass of HPV+ carcinomas comprising ~45% of HPV+ HNSCC that is not associated with any of these classic features. Patients in this subgroup have robustly improved clinical outcomes, and cell models with genomic and transcriptomic features of this class have increased sensitivity to radiation. The recognition of biologically distinct tumor subclasses of HPV+ HNSCC with differential responses to radiotherapy may fundamentally alter how patients are treated.

The incidence of Human papillomavirus-associated (HPV+) head and neck squamous cell carcinoma (HNSCC) has dramatically increased over the last few decades and continues to rise (1, 2). Due to the long latency from infection to tumor formation, HPV vaccination is not expected to impact this epidemic until 2060 (3). Despite the higher cure rate associated with HPV positivity, 20 to 30% of patients with HPV+ HNSCC suffer recurrence and have limited curative options (4–7).

Aggressive multimodality therapy developed for HPV-negative HNSCC is used for HPV+ patients and results in better survival compared to HPV-negative patients. Treatment-related morbidity coupled with relatively good outcomes in HPV+ HNSCC has sparked interest in therapeutic de-escalation for these patients (1, 8). Presently, deintensification of therapy to reduce morbidity is being frequently studied in patients with locally advanced disease, and preliminary results indicate that in nonsurgical patients, the radiation dose or field, but not chemotherapy, can be reduced (4, 9–11). However, these deintensification studies exclude many patients because of disease extent or tobacco history and likewise may include patients with aggressive tumors despite early stage and minimal smoking history. Since standard intensive therapy fails to cure ~30% of patients (4, 6), appropriate patient selection has been a persistent barrier for the expansion of de-escalation strategies. As such, there is a growing demand for molecular tools to guide personalized treatment.

HPV+ HNSCC research has focused largely on HPV oncogenes *E6* and *E7* and how viral integration promotes carcinogenesis, tacitly assuming that HNSCC biology would mirror the historically more common HPV-induced cervical cancer. However, several key differences between cervical cancer and HPV+ HNSCC contradict the validity of this assumption, including near-complete absence of HPV type 18 in HNSCC (12, 13), and distinct gene and protein expression (14, 15), as well as mutation (16–19), profiles. Moreover, HNSCCs more commonly maintain expression of all HPV16 early genes and lack viral integrations (20–22). These differences suggest a broader understanding of oncogenesis in this relatively understudied disease is warranted (19).

Therefore, we hypothesized that tumors lacking classical features of HPV-driven carcinogenesis (loss of *E2*, viral integration, *PIK3CA* alteration, and APOBEC mutagenesis) might represent a group of tumors with alternate requirements for tumor development. To address this question in an unbiased fashion, we turned to unguided analysis of transcriptomic data. Transcriptome-wide profiling has transformed the classification of some cancer types impacting prognostication and early detection (23). Gene expression signatures have been widely used to identify important characteristics of genotypes or phenotypes of interest (24–31). The development of statistical tools to address gene set abundance in the context of the larger transcriptome has solidified the utility of gene expression signatures in the study of biology (32–34).

Given the high dimensionality of transcriptomic data, it is not surprising that gene sets often can be identified with strong correlation to phenotypes using single datasets (35), but many of these signatures become less cohesive and lose the power to predict phenotype when applied to external datasets (36). Increasing amounts of publicly available transcriptomic data will hopefully accelerate the identification of robust gene expression signatures that are transferrable across labs, experimental techniques, and cohorts of patients for a disease of interest (36). Therefore, we analyzed our transcriptional data from 104 HPV+ HNSCCs in tandem with two publicly available sources to identify gene sets that were detected consistently, despite heterogeneous sequencing and quantification methodologies.

Surprisingly, we identified a single transcriptional program that naturally subclassifies HPV+ HNSCC tumors based on a bimodal pattern of gene expression, clusters all atypical features of HPV+ HNSCC biology into a single subclass, and predicts patient outcome. The gene expression signature and related radiosensitivity were recapitulated in HPV-positive head and neck cancer cells in culture, validating our translational findings.

## Results

### Defining Robust Transcriptional Programs in HPV+ HNSCC.

**Fig. 1.**
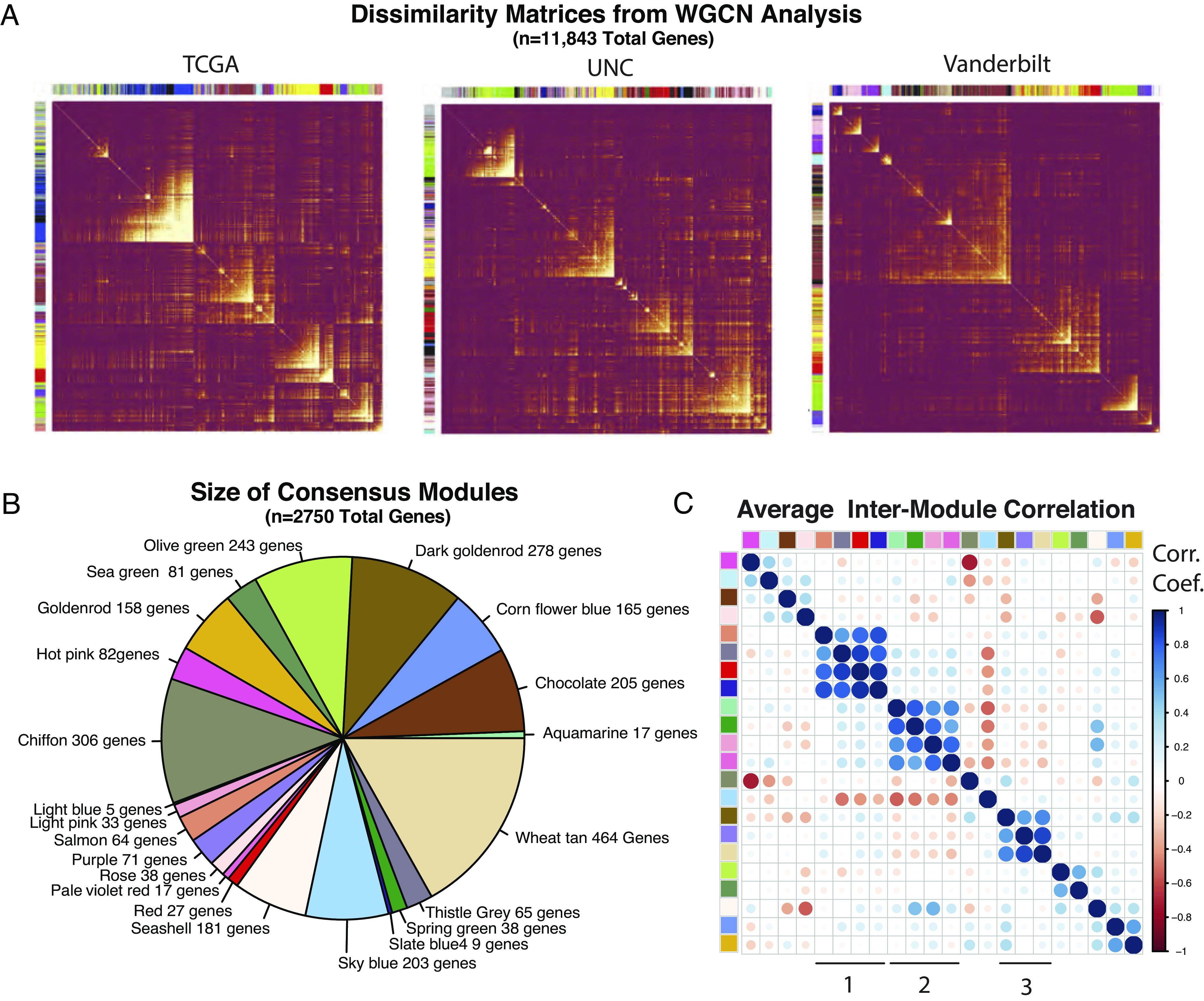
WGCNA of multiple cohorts reveals consensus transcriptional modules in HPV+ HNSCC. (*A*) Clustered dissimilarity matrices used for module selection in the WGCNA procedure. Columns/rows—sampled genes (n = 1,000) with module assignment. (*B*) Relative size of consensus modules. (*C*) Average intermodule correlation across all datasets. The mean Spearman's correlation coefficient is displayed. Color key as in panel *B*. Groups of modules with consistent intermodule correlation are indicated with numerals 1 to 3.

To identify robust gene expression modules indifferent to sequencing strategy and technical variability, we analyzed three independent datasets (16, 37). After normalization and filtering of very low expression genes, 11,843 genes were available in all three datasets. Internally correlated gene sets were identified from the three cohorts using weighted gene correlation network analysis (WGCNA). Dissimilarity matrices are displayed in Fig. 1*A*. In each dataset, genes were binned into groups (modules) with similar expression patterns using the WGCNA dynamic tree cutting algorithm (38). Consensus modules were then created by selecting genes that grouped together in WGCNA analyses from all three independent cohorts. To eliminate spurious assignment of consensus in the case of large modules, consensus modules were also required to comprise at least 5% of the largest single dataset module. Consensus module size ranged from 5 to 464 genes (Fig. 1*B*). As expected, intermodule correlation testing revealed relatively few consistent relationships between consensus modules (Fig. 1*C*), whereas many were apparent when examining single datasets (*SI Appendix*, Fig. S1). A list of genes included in each consensus module is provided in *SI Appendix*, *Dataset Module Definitions*.

### Gene Expression Module Characteristics and Quality Metrics.

**Fig. 2.**
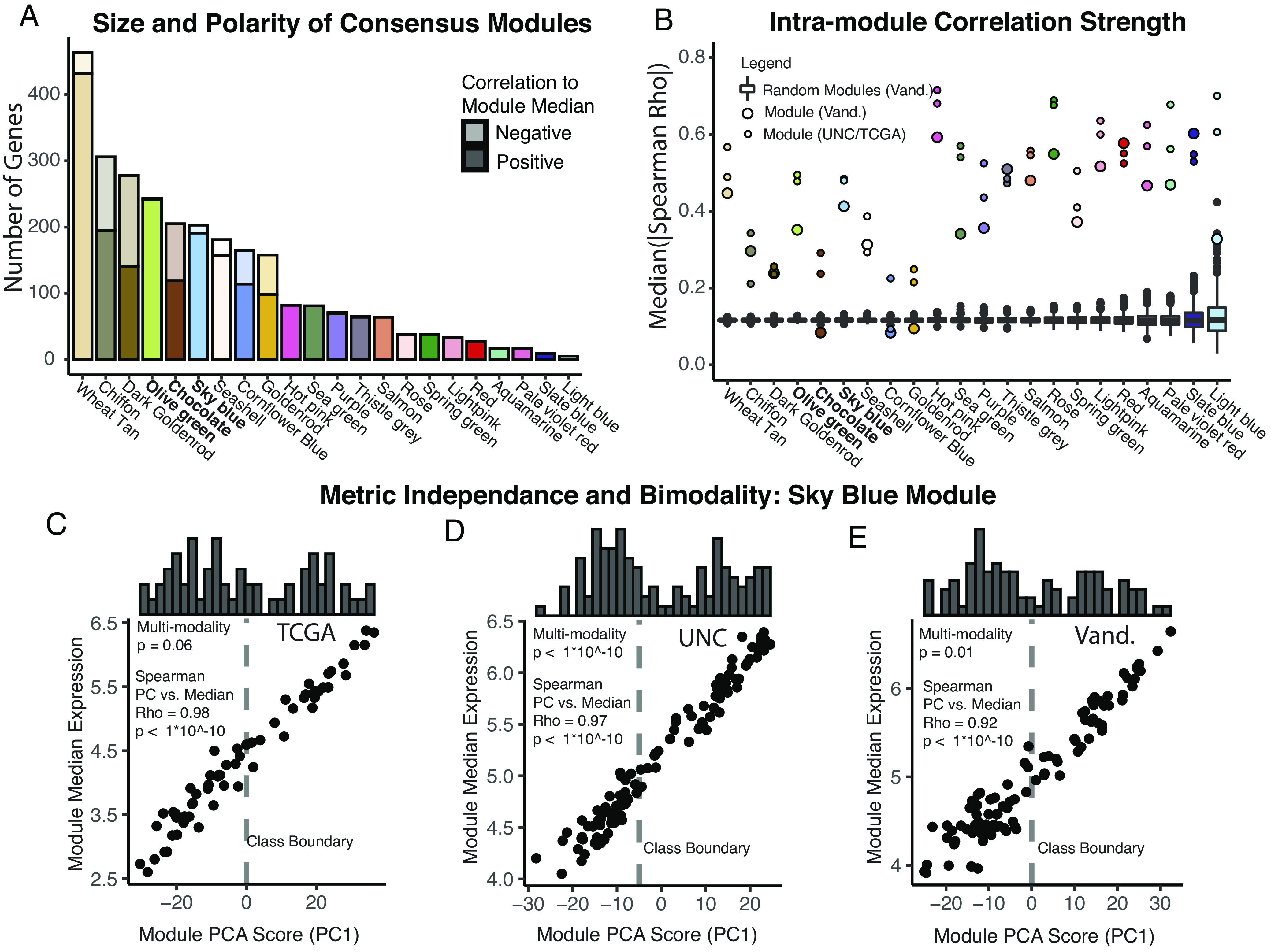
Module characteristics and quality metrics. (*A*) Bar plot of size and relative polarity of the consensus modules. Opaque—genes with expression positively correlated to the median expression of module genes. Transparent—genes with expression negatively correlated to the median expression of module genes. (*B*) Comparison of intramodule correlation of consensus modules to background. Box plot with black points—1,000 random gene sets of the size of the module in question were created. The median absolute value of Spearman's Rho is plotted for each resampling. The Vanderbilt cohort was used for this analysis, but resampling was also performed for TCGA and UNC which did not alter the results significantly. Colored points—median absolute value of Spearman's Rho for the consensus modules in all three datasets. (*C*–*E*) Ideal module characteristics of the Sky Blue module. Scatter plot—comparison of principle component 1 values to the median expression of all module genes. Histogram—frequency of PC1 scores. Correlation statistics—Spearman. Multimodality testing—excess mass method. Dashed line—empiric class boundary used for subsequent analyses.

WGCNA modules are defined independent of polarity, that is anticorrelated genes may be grouped together; however, the polarities are important for biological interpretation. The gene numbers and polarities of comprising genes for each module are summarized in Fig. 2*A*. Since module compactness (intramodule correlation) is a key quality metric for gene expression signatures (36), the compactness of modules was compared to that of randomly selected genes showing that consensus module genes were correlated (intramodule correlation) far above background (Fig. 2*B*). Interestingly, modules with higher levels of negatively correlated genes (chiffon, dark goldenrod, chocolate, cornflower blue, and goldenrod) had weaker intramodule correlation despite the use of the absolute value of Spearman’s Rho. In a few cases (chocolate, cornflower blue, and goldenrod), intramodule correlations were no better than randomly selected genes, suggesting that these modules were of lower quality. The compactness of gene expression modules can also be assessed with principle component analysis (36), specifically examining how much variance of the module genes can be explained by one principal component. Although polarity independent, the bidirectional modules were also less well described by one principal component (*SI Appendix*, Table S1).

We also assessed modules for evidence of multimodality distribution of gene expression across tumor samples using excess mass-based testing (39). Interestingly, only the *sky blue* module displayed multimodality in all cohorts with tumors grouping to high and low expression (Fig. 2 *C*–*E* and *SI Appendix*, Table S1). Quantification metric independence is also a key feature of high-quality gene signatures (36). Quantification of the *sky blue* module was also highly metric independent, as seen by the correlation between the PC1 score and median of expression (Fig. 2 *C*–*E*). Similar metrics for each consensus module are presented in *SI Appendix*, Table S1.

### Gene Set Enrichment Testing.

**Fig. 3.**
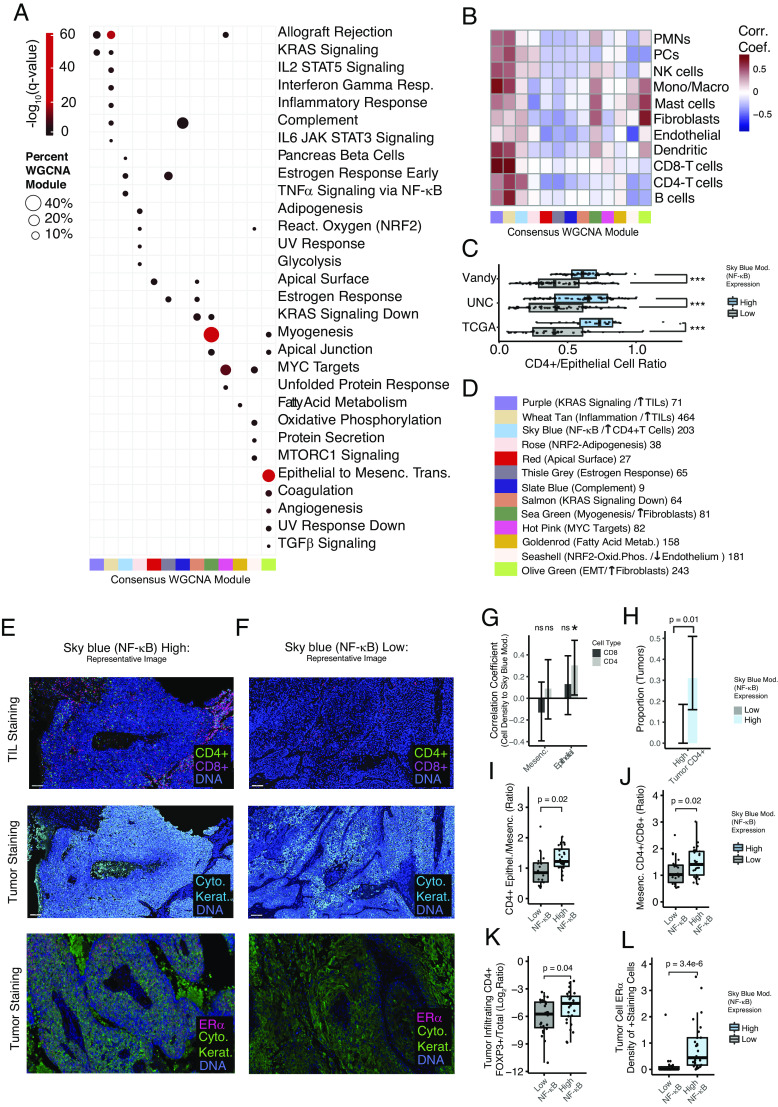
Hypergeometric enrichment analysis of HPV+ HNSCC consensus modules and correlation to tumor microenvironment. WGCNA modules were screened for enrichment in Hallmark gene sets from MiSigDB. Warmer colors represent lower adjusted *P* value (q value). Only results with q < 0.05 were displayed. The percent of module genes in the Hallmark gene set is represented by point size. Q values represent hypergeometric enrichment as reported by the EnrichR R package. (*A*) Enrichment analysis of individual consensus modules. (*B*) Heatmap of correlation coefficients. EcoTyper was applied to three datasets to estimate the proportion of cell types. The log2 (cell type intensity/epithelial intensity) was correlated to WGCNA module expression. The average Spearman's Rho considering all three datasets is displayed. (*C*) Box plots comparing the ratio of CD4+ T cell to epithelial cells in all three cohorts, stratified by expression of the sky blue (NF-κB) module. Significance tested with the Wilcoxon test. (*D*) Graphical summary of WGCNA modules with associated biological processes and tumor microenvironment, as well as the module size (number of genes). (*E* and *F*) Representative images of immunofluorescent staining of HPV+ HNSCCs from the UNC cohort. (*E* and *F*) tumors with low and high CD4+ cell infiltration into the tumor epithelial component, respectively. (*G*) Correlation between CD8+ and CD4+ cells in the tumor epithelial regions and expression of the sky blue (NF-κB) module. Pearson correlation coefficients with 95% CIs are provided. (*H*) Proportion of tumors with high CD4+ to nuclear staining ratios in the epithelial tumor regions. Significance based on the chi-squared test. (*I*) Box plot, comparing proportions of CD4+ cells in the tumor mesenchymal vs. epithelial components. Two outlier points related to low CD4 scoring in the stroma are not visualized for clarity but were included in statistical analysis. (*J*) Box plot, comparing proportions of CD4+ to CD8+ cells in the tumor mesenchymal component. (*K*) Box plot, showing proportions of FoxP3+CD4+ vs. CD+ cells in the tumor component. One outlier point with no FoxP3 staining was imputed to the lowest detectable value of FoxP3+; results were unchanged if this data point was excluded from the analysis. (*L*) Box plot, displaying the relative density of high ERα staining cells in the tumor component. *(I–L)* Significance tested with the Wilcoxen test. (*G*–*L*) Epithelial tumor components were defined by central areas of cytokeratin-positive staining. *P value < 0.05. ***P value < 0.0005.

To begin determining the biological context of identified modules in HPV+ HNSCC, hypergeometric enrichment analysis was performed for MSigDB Hallmark gene sets, (Fig. 3*A*). Estrogen receptor alpha expression, defects in NF-κB regulators, and immune infiltration have been correlated with outcomes in HPV+ HNSCC. Interestingly, the *sky blue* module associated with NF-κB signaling and early estrogen response genes, and because of this association, we dubbed the *sky blue* module the NF-κB related module. The *wheat tan* module associated with various signatures related to inflammation, and therefore may correlate with tumor immune infiltration, and the *thistle grey* module associated with early and late estrogen response genes. A total of 30 Hallmark gene sets had a significant association with the consensus WGCNA modules; of these, 19 were associated with only one module (Fig. 3*A*). Only the Hallmark Allograft Rejection signature (1/30) was associated with more than 2 WGCNA modules, suggesting that these consensus modules are relatively specific for associated biological functions.

We identified three groups of consensus modules containing strong intermodule correlations across all cohorts (Fig. 1*C* No. 1 to 3) and interrogated whether they might also have biological relevance using a similar enrichment analysis. While these module groups (meta-modules) retained associations identified by analysis of the individual modules, no additional biological associations arose when modules were analyzed as a group.

### Tumor Microenvironment (TME).

Since the modules were developed from bulk tumor sequencing, both cancer cells and components of the TME were sequenced. Using EcoTyper, a non-negative matrix factorization-based approach, we estimated the proportion of various cell types in the TME (40). The average correlation between the consensus module expression and TME components across the three cohorts is displayed in Fig. 3*B*. Because of the bimodal distribution of tumors in the NF-κB module, we were particularly interested in the correlation between the *sky blue* module and CD4+ T cells. To begin determining whether CD4+ T cell abundance in tumors correlated with identified subclasses, we queried CD4 signatures in high and low expressers (Fig. 2 *C*–*E*). Across all three cohorts, CD4+ T cells in tumors were elevated in the NF-κB (*sky blue*) high samples compared to NF-κB low samples (Fig. 3*C*). A graphical summary of the biological profiling associated with gene expression modules, including both gene set enrichment and TME analyses, is presented in Fig. 3*D*.

Using a subset of tumors with available FFPE from the UNC cohort (n = 50), we validated the RNA-based findings related to CD4+ cell infiltration (Fig. 3 *E*–*J*). Interestingly, the CD4+ cells correlated with the NF-κB module exclusively in the epithelial component defined by cytokeratin staining (Fig. 3*G*), but not in mesenchymal areas of the tumors. The nine tumors with the most tumor-invading CD4+ cells were all in the high *sky blue* (NF-κB) expressing group, Fig. 3*H*. The relative proportion of CD4+ cells in the epithelial component versus the mesenchymal component was also increased in the NF-κB high group, consistent with the correlation being specific to the regions of epithelial (cancer) cells (Fig. 3*I*). The ratio of CD4+ to CD8+ cells in the tumor mesenchymal tumor component was also distinct, with the high NF-κB expressers having a higher mesenchymal CD4+/CD8+ ratio (Fig. 3*J*). Based on these initial findings, we also performed multiplex immunofluorescence microscopy on the same samples staining for FoxP3 and CD4. We found that NF-κB high tumors had a higher proportion of FoxP3+/CD4+ T cells, suggesting that they may be enriched in regulatory T cells (Fig. 3*K* and *SI Appendix*, Fig. S2). Considering the correlation between the *sky blue* (NF-κB) module and early estrogen response genes and the reported prognostic value of ERα expression (41), we examined estrogen receptor alpha (ERα) expression and found that the ERα level in tumor cells was highly associated with expression of the NF-κB module, (Fig. 3*L*).

### Somatic Variant Analyses.

**Fig. 4.**
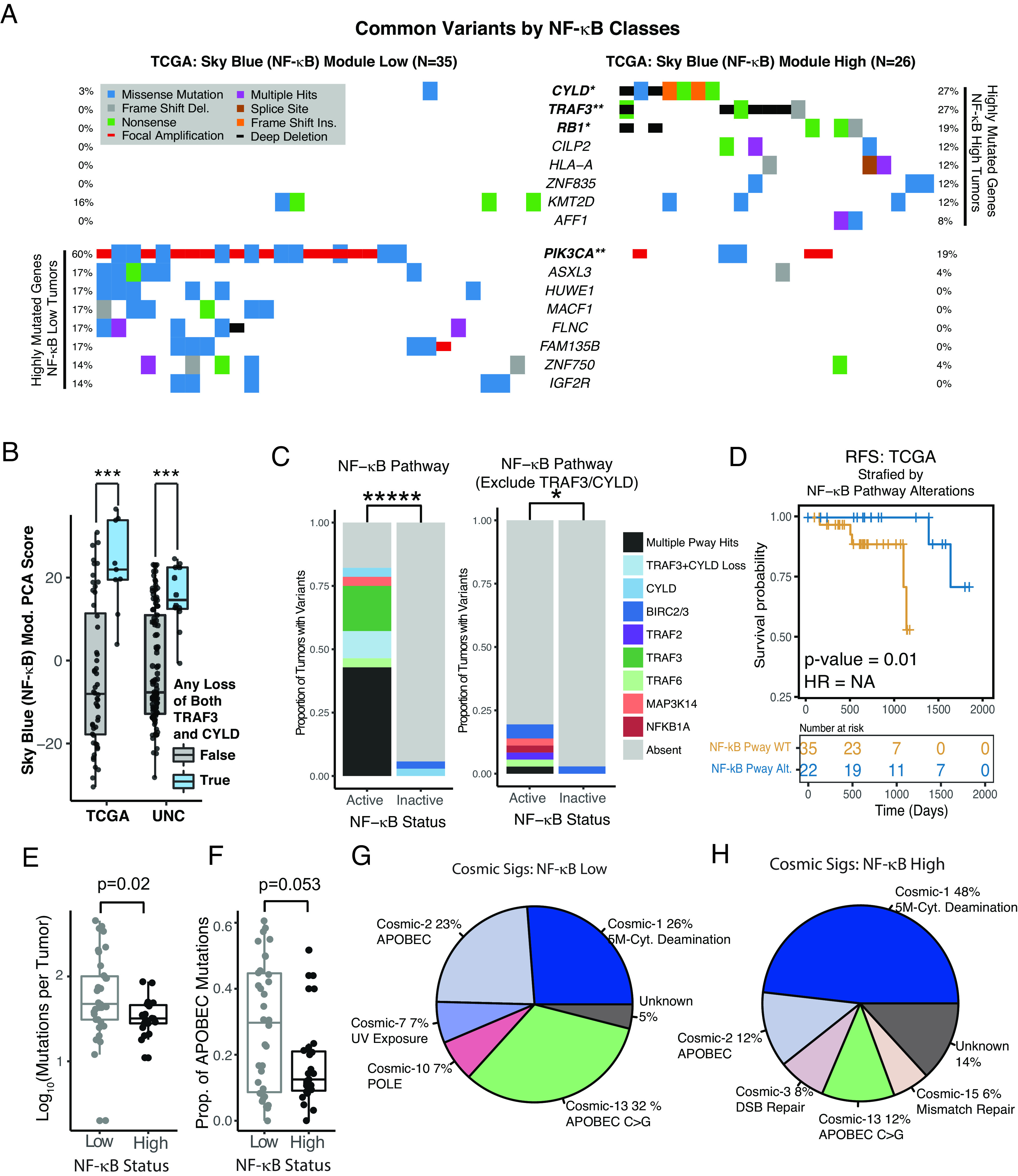
Genotypic associations with the sky blue (NF-κB) module. (*A*) Waterfall plot and frequency of gene alterations in tumors with low (*Left*) or high (*Right*) expression of NF-κB (sky blue) module. The top 8 nonredundant genes in terms of alteration frequency in both groups are displayed, excluding MUC16 and TTN. **z-test of equal proportions *P* value < 0.005. *z-test of equal proportions *P* value < 0.05. (*B*) Box plot comparing sky blue (NF-κB) module expression in tumors with and without simultaneous low (arm) level copy loss of both TRAF3 and CYLD. TCGA—Tumors with GISTIC scores of −1 for both TRAF3 and CYLD are highlighted in blue and compared to those without. UNC—Copy number log2Ratio values were estimated for TRAF3 and CYLD using the CNVkit RNA pipeline. Tumors with log2Ratio < −0.35 for both the TRAF3 and CYLD loci are highlighted in blue and compared to those without. ***Wilcoxon rank-sum test *P* value < 0.0005. (*C*) Pathway oriented mutational analysis of NF-kB pathway genes, stratified by (NF-κB) module low vs. high expression tumors. *(D)* Recurrence free survival of patients with HPV+ HNSCC, stratified by NF-kB pathway alterations, as in panel C. P value represents log-rank test. (*E*) Box plot of the number of somatic SNPs per tumor stratified by expression of the sky blue (NF-κB) module. (*F*) Box plot of the proportion of APOBEC-related somatic SNP per tumor stratified by expression of the sky blue (NF-κB) module. APOBEC-related SNPs as defined by the maftools function trinucleotideMatrix() function. (*G* and *H*) Pie charts showing relative abundance of mutagenic signatures in tumors with low vs. high expression of the sky blue (NF-κB) module. Values represent non-negative matrix factorization weights for the COSMIC2 signatures.

Next, we examined which genes were most frequently altered in the tumors with high versus low expression in the NF-κB module. Publicly available TCGA mutation and copy number calls were downloaded and used without modification (42). Mutations in large genes (Titan, MUC16), known to be primarily associated with tumor mutational burden, were excluded from analysis. Interestingly, alterations in a known head and neck cancer driver, *PIK3CA,* were strongly enriched in the low NF-κB group. Conversely, NF-κB regulatory genes (*CYLD* and *TRAF3*) were nearly exclusively altered in the high NF-κB group (Fig. 4*A*). Notably, *RB1* mutations and deletions were also enhanced in the NF-κB high group. An association between *RB1* and *CYLD* alterations has been previously reported (43).

Considering that single copy (GISTIC score = −1) losses in *TRAF3* and *CYLD* were quite common in the TCGA cohort, we asked whether low level but simultaneous loss of these NF-κB regulators associated with expression of the *sky blue* module. Indeed, tumors with single copy loss of *TRAF3* and *CYLD* had higher expression of the *sky blue* module genes, (Fig. 4*B*). To validate this finding, we performed copy number profiling based on the UNC cohort using the CNVkit RNA pipeline, which identifies large-scale copy number aberrations based on regionally correlated patterns of RNA expression. In the UNC cohort, tumors with simultaneous chromosomal arm level losses (log_2_Ratio < −0.35) in both *TRAF3* and *CYLD* also demonstrated increased expression of the NF-κB module (Fig. 4*B*).

To determine whether other NF-κB pathway components were altered in the high NF-κB group, we performed pathway-oriented mutational analysis, examining a set of 9 NF-κB genes reported to be altered in other NF-κB-driven cancers (Fig. 4*C*). Interestingly, this analysis revealed that many tumors in the NF-κB active group harbored multiple pathway alterations and that ~80% of NF-κB active tumors had some NF-κB pathway change. Excluding TRAF3 and CYLD, other NF-κB pathway alterations were also detectably enhanced in tumors with high expression of the *sky blue* module, Fig. 4*C*. NF-κB pathway mutations were predictive of recurrence-free survival in TCGA data, Fig. 4*D*.

To determine whether differences in mutagenic processes underlie or parallel differences in gene defects, we analyzed single nucleotide polymorphisms (SNPs) and their trinucleotide context, Fig. 4 *E*–*H*. NF-κB low tumors had significantly more SNPs, (Fig. 4*E*). C > G mutations were the most common in both groups, but NF-κB low tumors demonstrated more trinucleotide context specificity (*SI Appendix*, Fig. S3). Related to this, the fraction of APOBEC-related variants (as quantified by Maftools) was marginally higher in the NF-κB low group (Fig. 4*F*). Evaluation of COSMIC signatures in the subgroups revealed that APOBEC mutagenesis was associated with more than 50% of mutations in the NF-κB low group, compared to only 24% in the NF-κB high group (Fig. 4 *G* and *H*). Taken together, these results reveal distinct patterns of somatic alteration and mutagenic processes in high vs. low gene expression subgroups defined by the *sky blue* (NF-κB) module.

### Viral Gene Expression and Integration Analyses.

We previously reported that *TRAF3/CYLD* alterations in HPV+ HNSCC are associated with increased NF-κB activity and lack of viral integration (44). Integration of the HPV genome correlates with viral gene expression patterns (45), and increased expression of E6 and E7 is a hallmark of viral genomic integration in HPV+ HNSCC (45). To determine whether subsets of HPV+ HNSCC identified by high or low expression of the NF-κB module had differences in viral integration or viral gene expression, we investigated the TCGA and UNC cohorts. Viral integration was determined by read pairs that displayed discordant mapping to the human and HPV16 genomes (*SI Materials and Methods*). In our analyses, the ratio of E6 and E7 expression to E2 and E5 was most related to integration, and thresholds for high E6/E7 expression relative to E2/E5 were selected to best agree with integration calls.

**Fig. 5.**
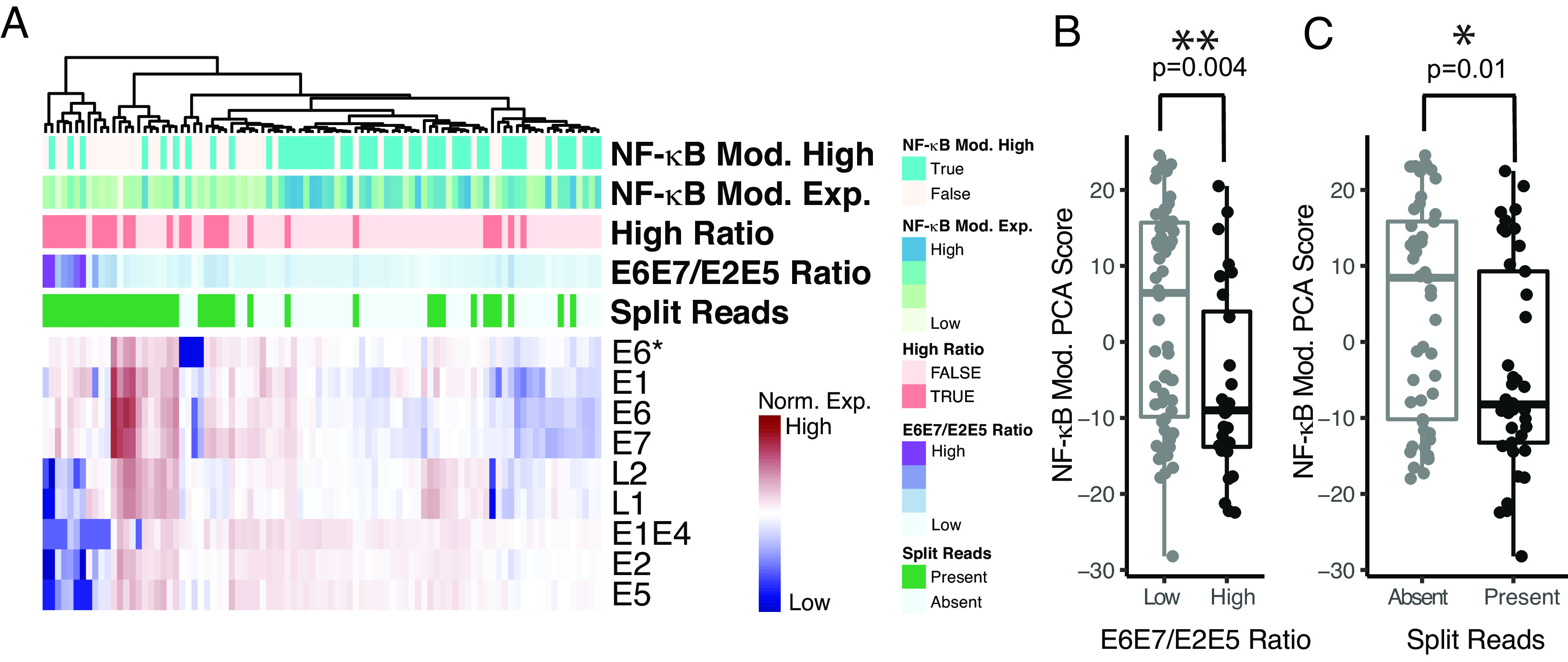
NF-κB module expression is related to patterns of viral gene expression and viral genomic integration. (*A*–*C*) Analysis of RNA sequencing (RNAseq) data from the UNC Cohort. (*A*) Annotated heat map of HPV16 viral gene expression. Columns—tumor samples, organized by clustering on viral gene expression, normalized to human RNA. Sky blue (NF-κB) module high—tumors with high expression of the sky blue (NF-κB) module genes based on the PCA score. Sky blue (NF-κB) module expression—principal component analysis score for the NF-κB (Sky Blue) module. E6E7/E2E5 Ratio—ratio of E6 and E7 expression to E2 and E5 expression. Log2[(readsE6+readsE7)/(readsE2+readsE5)]. Split reads—presence of detectable split read pairs mapping to both the HPV16 and human genome, as identified by the ViFi viral integration software package. (*B*) Box plot of sky blue (NF-κB) module PCA scores for tumors with low and high E6E7/E2E5 ratio, as defined in panel *A*. Significance based on the Wilcoxon rank-sum test. (*C*) Box plot of sky blue (NF-κB) module PCA scores for tumors with and without viral integration, as defined by split read pairs as in panel *A*. Significance based on the Wilcoxon rank-sum test. See *SI Appendix*, Fig. S4 for similar analysis of the TCGA cohort. *P value < 5*10^−2. **P value < 5*10^−3.

Unguided clustering based on viral gene expression demonstrated gross relationships to *sky blue* module, as well as expected relationships to viral integration status (Fig. 5*A* and *SI Appendix*, Fig. S4*A*). Tumors with high E6E7/E2E5 gene expression ratios were associated with lower expression of *sky blue* module genes (Fig. 5*B* and *SI Appendix*, Fig. S4*B*). Tumors with discordant read pairs supporting viral integration were associated with lower expression of *sky blue* (NF-κB) module genes, (Fig. 5*C* and *SI Appendix*, Fig. S4*C*) Taken together, these results demonstrate that tumor subclasses defined by the *sky blue* (NF-κB) module are associated with a lack of viral genomic integration and a distinct pattern of viral gene expression.

### Genomic Methylation Analysis.

**Fig. 6.**
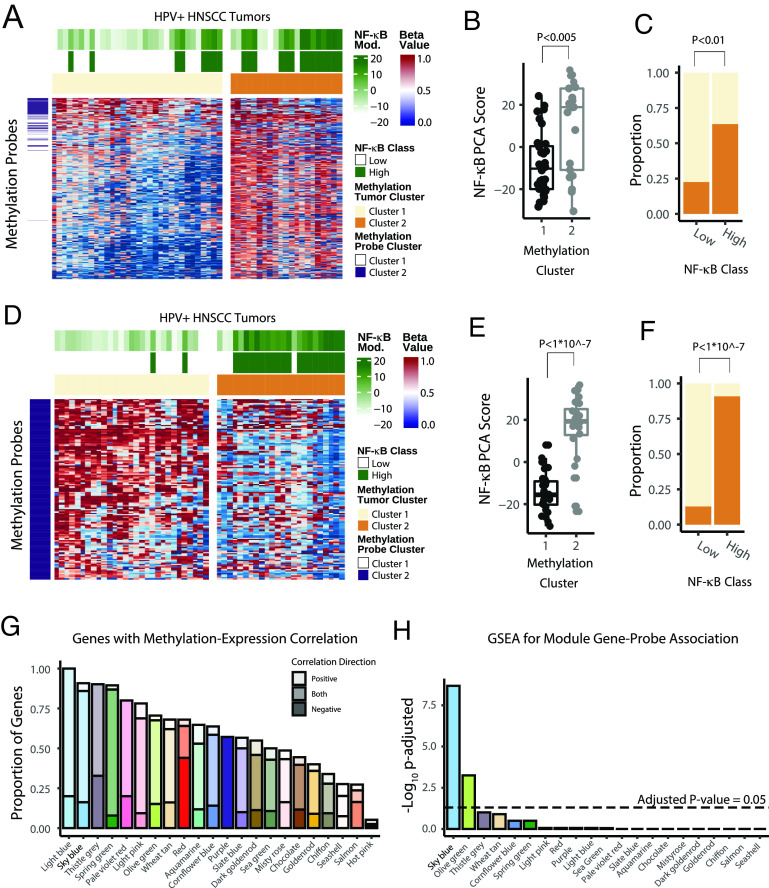
Analysis of genomic methylation in HPV+ HNSCC with consensus clustering. (*A*) Heatmap of methylation probe intensities for HPV+ HNSCC tumors in TCGA organized by consensus clustering (k = 2), see *Materials and Methods* for further details. Green color scale—NF-κB (sky blue) module principal component analysis (PCA) scores. Dark green—denotes high expression of the NF-κB (sky blue) module (as applied in panel *C*). Tan—tumors assigned to methylation cluster 1. Orange—tumors assigned to methylation custer 2. Warm colors—higher beta-values (more methylated). Cool colors—lower beta-values (less methylated). (*B*) Box plot of NF-κB (sky blue) module principal component analysis scores, stratified by methylation subgroup. Color scale as in panel *A*. The *P* value represents the Wilcoxon rank-sum test. (*C*) Proportion bar plot of methylation subgroup, stratified by high and low NF-κB (sky blue) module expression. Colors as in panel *A*. The *P* value represents the z-test of equal proportions. (*D*–*F*) Analysis of minor methylation probe cluster, defined by consensus clustering. *D*–*F* are identical to *A*–*C*, except only the minor group of methylation probes (panel *A*, dark purple) was included. (*G*) Barplot displaying the proportion of module genes with correlated changes in methylation in the area of the gene in question. (*H*) Barplot displaying GSEA-based adjusted *P* value representing the enrichment of each WGCNA module for probe–gene correlation.

**Fig. 7.**
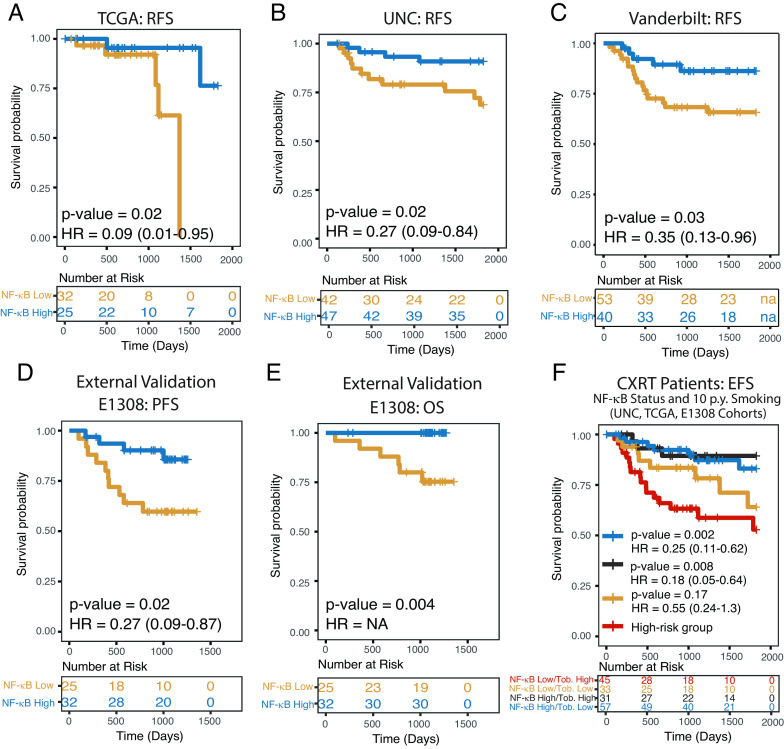
Increased expression of sky blue (NF-κB) module is associated with improved patient outcomes. Kaplan–Meier plots with number at risk tables below. (*A*–*C*) Recurrence-free survival (RFS) of patients included in WGCNA module analysis. (*D* and *E*) Progression-free survival (PFS) and overall survival (OS) of patients in E1308 clinical trial, a validation cohort not used in module development. (*F*) Combined analysis of event-free survival (RFS or PFS based on availability) in all patients treated with chemoradiation with available quantitative smoking data (UNC, TCGA, E1308). Hazard ratio CIs in parentheses represent 95% CI. *P* values represent the log-rank test. Follow-up was limited to 5 y.

Considering that HPV+ HNSCC tumors have hypermethylated genomes compared to HPV- HNSCC tumors, we compared global methylation differences across the tumor sample groups delineated by the *sky blue* (NF-κB) module and correlated expression of module genes located close to CpG islands. Using TCGA data, we performed consensus clustering across the samples using the 2,000 methylation probes with the highest variance among HPV+ HNSCC. This approach resulted in two groups of HPV+ HNSCC tumors, with relatively high and low levels of methylation across the included probes, see Fig. 6*A*. NF-κB (*sky blue*) module expression was higher in tumors in the more methylated group (Fig. 6*B*) with a corresponding enrichment for NF-κB high tumors in the high methylation group (Fig. 6*C*). Although the average beta-value across all probes was not significantly different between the NF-κB active and inactive groups, there was a trend toward hypermethylation in the NF-κB active tumors (*P* = 0.097, Wilcoxon test). This trend is consistent with the clustering on high all variance probes, as well as mutational signature analysis which revealed increased 5-methylcytidine deamination in NF-κB active tumors (Fig. 4*F*). Consensus clustering on the CpG probes identified a smaller (~130) group of probes (Figs. 6*A* and 7*D*, dark purple) with a tighter association to NF-κB (*sky blue*) module expression upon repeat clustering (Fig. 6 *D*–*F*). Interestingly, the probes identified in this unguided manner were largely anticorrelated with the global differences in methylation levels, with these probes showing lower methylation in the NF-κB active group (Fig. 6 *D*–*F*).

Given the relationship between the NF-κB module and gross patterns of genomic methylation, we analyzed the other consensus modules for methylation sites that could influence module gene expression. Methylation probe–gene pairs for each module were identified if probes were proximal in the genomic space and had a high correlation between expression and methylation. We then used robust rank aggregation to control for the number of probes associated with each gene and to produce a gene-level *P*-value (*SI Appendix*). We found that ~80% of the genes in the *sky blue* (NF-κB) module had significant expression-methylation association (Fig. 6*G*). To assess the relative degree to which the expression–methylation correlations in identified modules were stronger than expected at random, we calculated Gene set enrichement analysis scores for each module compared to the distribution of gene-level methylation-expression correlations across all genes. We found that the *sky blue* (NF-κB) module was by far the most enriched compared to all other modules (Fig. 6*H*). In summary, the methylation analysis demonstrated that global epigenetic programming is different between the NF-κB high and low tumors and highlighted a strong relationship between methylation of *sky blue* (NF-κB) module GpC probes and RNA expression of this gene module.

### Survival Analysis.

Considering the general interest in developing predictive biomarkers for guiding therapy in HPV+ HNSCC, we looked for correlations with outcome. For modules that did not intrinsically separate tumors into 2 classes (all but *sky blue*), the median module principal component analysis score was used as the cut point to divide tumors into 2 groups (high and low). For the bimodal *sky blue* module, tumors were stratified by an empiric threshold based on the nadir between the two expression peaks (Fig. 2 *C*–*E*). Interestingly, the *sky blue* (NF-κB) module was the only gene set that segregated patients into groups significantly associated with differences in recurrence-free survival (RFS) in all three cohorts (Fig. 7). Finding that the NF-κB-related module intrinsically segregated patients into two groups with better or worse prognosis confirms the significance of the biological differences between these tumor groups.

**Fig. 8.**
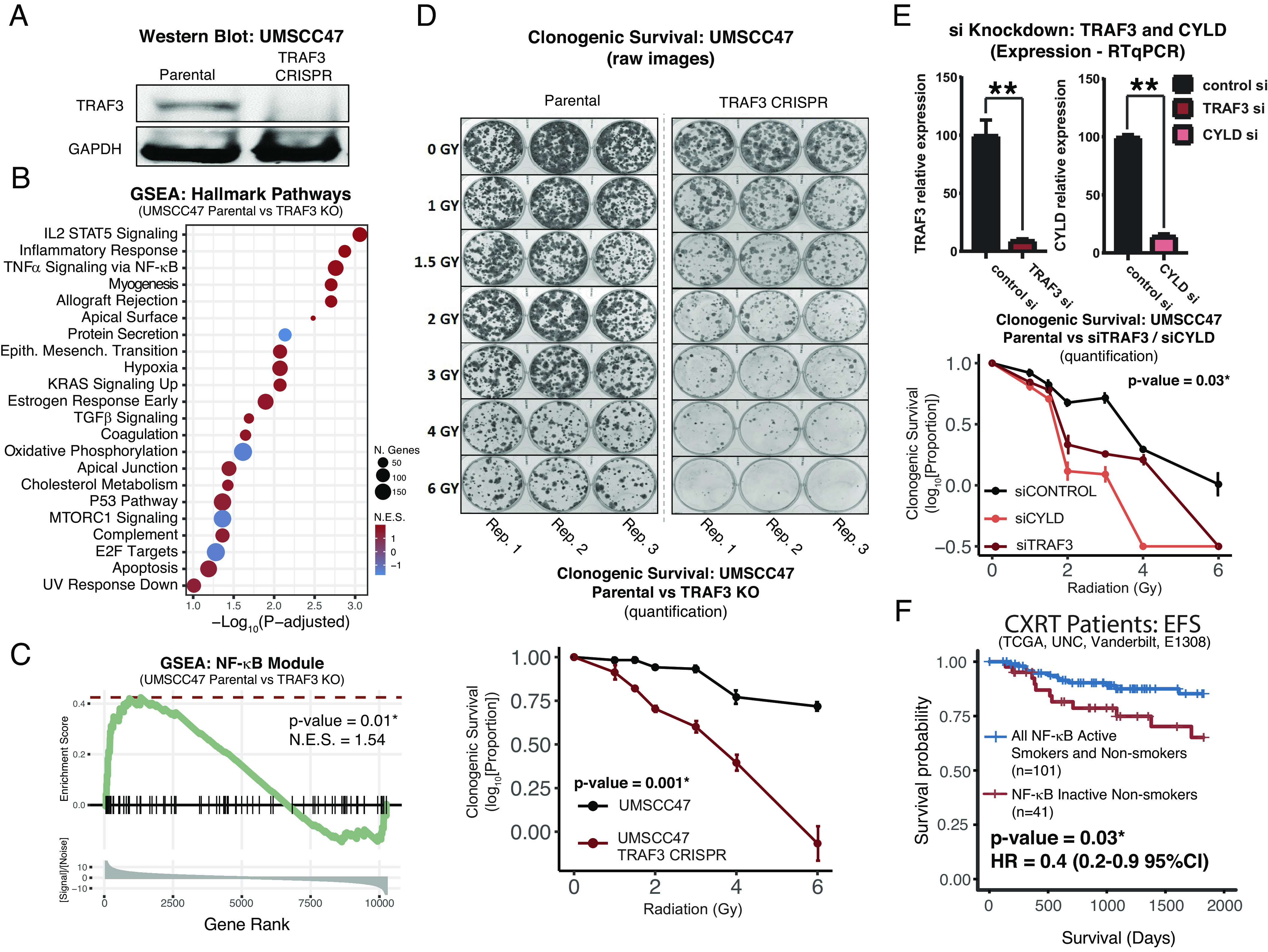
Noncanonical oncogenic alterations activate NF-κB and radiosensitize HPV+ HNSCC cancer cells. (*A*) Immunoblot confirming CRISPR knockout (KO) of *TRAF3* in UMSCC47 cells. (*B* and *C*) RNAseq of UMSCC47 cells with and without TRAF3 KO. (*B*) Gene set enrichement analysis (GSEA) for MSigDB Hallmark gene sets, ranked by adjusted *P* value. Gene sets with *P* > 0.1 are not displayed. (*C*) GSEA of the *sky blue* (NF-κB) module genes. Genes anticorrelated to the module median (12 genes, 6% of module) were excluded. (*D*) Clonogenic survival of UMSCC47 cells with and without TRAF3 KO. *Top*—raw images. *Bottom*—quantification. (*E*) Small interfering RNA knockdown of TRAF3 and CYLD. *Top*—RTqPCR confirmation of knockdown. *Bottom*—quantified clonogenic survival. (*F*) Improved clinical outcomes of NF-κB active patients as compared to NF-κB inactive nonsmokers. Nonsmokers defined as less than 10 pack-years of tobacco smoke exposure. Event-free survival (EFS) represents combined progression-free survival or recurrence-free survival based on availability.

To provide external validation of these clinical findings, we performed RNA sequencing of 57 patients with available Formalin-Fixed Paraffin-Embedded (FFPE) tumor tissue from the ECOG-ACRIN E1308 clinical trial. Tumor groups were defined based on the expression of *sky blue* (NF-κB) module genes based on an empirical threshold. We found that in these patients treated with induction chemotherapy followed by chemoradiation, both progression-free survival and overall survival were improved in the group expressing higher levels of *sky blue* (NF-κB) module genes(Fig. 7 *D* and *E* and *SI Appendix*, Fig. S5). To understand the relationship between disease stage, tobacco smoke exposure, and NF-κB status, we pooled all patients treated with chemoradiation, for whom quantitative smoking data were available (TCGA, UNC, E1308). Using this combined cohort of 166 patients, multivariate analysis was performed and demonstrated that NF-κB active tumors were associated with less tobacco smoke exposure and less tumor stage 4 disease (*SI Appendix*, Tables S2–S4). Multivariate event-free survival analysis of this pooled cohort demonstrated that NF-κB status was not only prognostic while controlling for stage and tobacco smoke exposure (*P* = 0.005) but was also the most prognostic factor in all models we examined (*SI Appendix*, Table S5). Interestingly, although tobacco smoke exposure is an accepted adverse prognostic factor in HPV+ HNSCC, our data suggest that this association is driven exclusively by NF-κB inactive tumors (Fig. 7*F*). To determine whether NF-κB status was relevant to outcomes in patients with low or no tobacco smoke exposure, who might be considered candidates for deintensified therapy, we compared all patients treated with chemoradiation with no or less than 10 pack-years of smoking history to all NF-κB active patients treated with chemoradiation. We found that the NF-κB active group had significantly improved (HR = 0.4, *P* = 0.02) event-free survival (RFS or progression-free survival, based on availability) (Fig. 8*F*).

### Oncogenic NF-κB Activation Promotes Radiation Sensitivity in HPV+ HNSCC.

Locoregionally advanced HPV+ HNSCCs are usually treated with radiation, either in the primary or postoperative setting. Our data show that patients whose tumors display high expression of the *sky blue* module have significant survival benefits that are most likely explained by increased sensitivity of tumors with constitutively active NF-κB to radiation. Therefore, we hypothesized that NF-κB activity may contribute to intrinsic tumor cell characteristics that increase radiation sensitivity in HPV+ HNSCC. Indeed, we found that experimental *TRAF3* and *CYLD* inactivation was sufficient to strikingly sensitize HPV+ head and neck cancer cells to radiation, as well as promote increased expression of well-known NF-κB targets and *sky blue* (NF-κB) module genes (Fig. 8 *A*–*E*). Thus, our data support the premise that NF-κB signaling, which drives an alternative mechanism of HPV carcinogenesis, sensitizes cells to radiation through cancer cell–intrinsic effects.

## Discussion

Using three independent HPV+ HNSCC cohorts, an unbiased approach identified robust gene expression profiles existing in these tumors (Figs. 1 and 2) and identified the major molecular pathways and cell types associated with each of these expression profiles (Fig. 3).

Our group previously identified an etiologically distinct subset of HPV+ HNSCC characterized by the inactivation of *TRAF3* or *CYLD* leading to constitutively active NF-κB (44). This prior work correlated the inactivation of *TRAF3* and *CYLD* with improved patient survival and a lack of HPV integration, suggesting that NF-κB activation may enable cells to maintain HPV episomes as a distinct mechanism of carcinogenesis (44). In the current study, 22 expression modules were identified, but only one had ideal statistical characteristics for tumor subclassification, namely a bimodal distribution in all three independent cohorts (Fig. 2); this *sky blue* module was strongly associated with activated NF-κB signaling and contained all tumors with *TRAF3* and *CYLD* inactivating defects (Figs. 3 and 4). Herein, we also report that tumors with simultaneous shallow (single copy) losses at both, *CYLD* and *TRAF3,* loci were robustly associated with high expression of the *sky blue* (NF-κB) module (Fig. 4), suggesting that mechanisms in addition to deep deletion of *TRAF3* or *CYLD* modulate NF-κB activation in HPV+ HNSCC. Applying pathway-based analysis, we find that while individually rare, when viewed as a group, mutations in other NF-κB pathway genes were also more common in tumors with NF-κB activation (Fig. 4). We also found that RB1 deletions and mutations were significantly enriched in the NF-κB active group (Fig. 4). Interestingly, constitutively active NF-κB has been shown to reduce p53 (46), as well as Rb activity, while phosphorylated Rb can inhibit NF-κB (47), suggesting that the loss of Rb function may serve an important oncogenic role in tumors dependent on highly active NF-κB. An association between *RB1* mutations and *CYLD* mutations in HPV+ HNSCC has recently been reported by other groups (43).

APOBEC3 cytidine deamination hypermutates viral genomes, including HPV (48–51), and deep sequencing of human tumors revealed that APOBECs are major sources of mutations in several cancers, including HNSCC, where they create driver mutations (e.g., *PIK3CA*) (49). We recently reported that HPV+ tumors containing APOBEC signatures had markedly higher mutational burden compared to tumors lacking APOBEC mutations (49). The data presented here further reveal that APOBEC mutation signatures, as well as APOBEC-dependent *PIK3CA* mutations, are significantly more frequent in NF-κB inactive tumors (Fig. 4), which is consistent with the observed relative importance of APOBEC mutagenesis across the entire genome in the NF-κB inactive group (Fig. 4). We also find that missense mutations and focal amplifications of *PIK3CA* are present predominantly in the NF-κB inactive group and are relatively uncommon in the NF-κB active group (Fig. 4). Consistent with our results, *PIK3CA* mutations have been associated with poor prognosis in HPV+ HNSCC (52). Considering that activation of *PIK3CA* may limit the utility of related targeted therapies (cetuximab), it is intriguing to consider that the NF-κB module may represent a biomarker for a subset of patients that are more responsive to this therapy.

The biological distinction between the two tumor groups defined by NF-κB module is further underscored by differences in the mutagenic processes (Fig. 4) and the genome-wide methylation patterns (Fig. 6) between the two groups. The relative generalized hypermethylation of NF-κB active tumor genomes (Fig. 6*A*) is consistent with the predominance of spontaneous deamination of 5-methylcytosine as a mutagenetic process in NF-κB active tumors (54% vs. 27% in NF-κB inactive tumors, Fig. 4). Interestingly, the *sky blue* (NF-κB) module had the highest correlation between gene expression and the methylation of nearby GpC loci (Fig. 6*H*), suggesting that cells with a methylation pattern permissive to NF-κB target expression might promote a more oncogenic phenotype when combined with NF-κB activating mutations. Interestingly, both APOBEC and spontaneous deamination of 5-methylcytosine mutation signatures were recently found in nasopharyngeal cancer that is often driven by the Epstein–Barr virus (53) and harbors inactivating mutations in NF-κB pathway regulators (54).

In line with our prior findings, here we show that NF-κB active tumors were associated with different patterns of viral gene expression and were less associated with HPV viral genomic integration (Fig. 5). Mechanisms explaining how a nonintegrated form of HPV may induce head and neck carcinogenesis are not well described. Considering that NF-κB is activated in various tumor types, and its activity promotes carcinogenesis through cellular proliferation, transformation, and angiogenesis, while protecting tumor cells from apoptosis (55), we hypothesize that activation of NF-κB due to genetic alterations in NF-κB regulators (e.g., *TRAF3* or *CYLD*, Fig. 4) in the context of permissive epigenetic landscape promoting expression of NF-κB target genes (Fig. 6*H*) represents a distinct pathway for HPV16 mediated oncogenesis in the oropharynx (*SI Appendix*, Fig. S6).

We further explored differences between subtypes based on NF-κB signaling and found that patients with tumors harboring constitutively active NF-κB had improved survival in all three independent cohorts (Fig. 7). This finding was validated by performing RNAseq of samples from the ECOG-ACRIN1308 clinical trial (Fig. 7). Furthermore, in multivariate analysis, NF-κB status was more prognostic than established clinical factors and also remained a significant predictor of outcome while controlling for these factors (*SI Appendix*, Table S5). HNSCCs are often treated with radiation; rates of response and cure are higher for patients with HPV+ compared to HPV-negative HNSCC; however, our data suggest that the majority of survival benefit is attributable to the subtype of HNSCC with highly active NF-κB (Fig. 4). Our data suggest that constitutively active NF-κB, determined by the presence of inactivating mutations in NF-κB regulators or by a specific RNA signature (56), may serve as prognostic biomarkers to help clinicians with therapeutic decisions. Other groups have suggested ERα expression as a prognostic marker for this purpose, and we herein show that groups defined by high ERα expression and NF-κB activity are largely identical (Fig. 3*L*) (41). Since NF-κB activity is most commonly associated with resistance to therapy, we searched for drivers of therapeutic sensitivity and found an increased number of tumor-infiltrating CD4+ cells (Fig. 3). Better prognosis and elevated sensitivity to radiotherapy of HPV+ HNSCC, as compared to HPV-negative tumors, has been recently correlated with increased infiltrating immune cells (CD8+ T and B cells) (57), and NF-κB activity has been shown to increase T cell infiltration (58, 59). CD4+ T cells have been implicated as both enhancers and inhibitors of antitumor immune response based on CD4+ T regulator or T helper status (60). A higher proportion of T regulatory cells in NF-κB active tumors (Fig. 3*K* and *SI Appendix*, Fig. S2) indicates a potential for immunotherapy. Importantly, activation of NF-κB via depletion of TRAF3 or CYLD resulted in elevated radiosensitivity of HPV+ head and neck cancer cells (Fig. 8). Thus, increased NF-κB activity in HPV+ HNSCC contributes to intrinsic tumor cell (Fig. 8) and TME characteristics (Fig. 3) that promote increased radiation sensitivity.

In summary, the data presented here reveal the existence of two intrinsically different subtypes of HPV-positive head and neck cancer. The NF-κB active subset of HPV+ HNSCC displays not only a distinct pattern of NF-κB related gene expression but also markedly different mutational and methylation profiles. The striking lack of *PIK3CA* variants, lack of HPV integration, and decreased APOBEC mutagenesis in the subtype of tumors with active NF-κB suggest that they are driven by an alternative mechanism of HPV-related carcinogenesis (*SI Appendix*, Fig. S6).

Our data also have important clinical implications. Clinicians treating HPV+ HNSCC are actively searching for actionable biomarkers to guide therapeutic intensity (61). These data, as well as our prior work (44, 56), demonstrate that two prognostic subclasses with different levels of NF-κB activity can be identified by many approaches related to their striking differences in many aspects of the cancer genomes. These groups are robustly prognostic and integrate prior observations related to the prognostic value of viral integration (62), *PIK3CA* mutations (52), and ERα expression (41). We believe that our data suggest that these two tumor groups harbor fundamentally different requirements for tumorigenesis and tumor maintenance. As such, we hope that the strong biological differences will also present opportunities to explore targeted therapies, which might be effective when applied with precision to these highly distinct tumor classes.

## Materials and Methods (For Detailed Materials and Methods, Please See *SI Appendix*)

Three different HPV+ HNSCC cohorts were used in our study to determine and characterize transcriptional modules and perform survival analysis: TCGA (has both, DNAseq and RNAseq), UNC (RNAseq and immunofluorescent staining), and Vanderbilt (RNAseq). To validate clinical benefits of a sky blue module, we performed RNAseq of an independent, prospectively collected and uniformly treated cohort from the E1308 trial. To investigate whether overactive NF-κB contributes to intrinsic tumor cell characteristics that increase radiation sensitivity in HPV+ HNSCC, we used HPV+ HNSCC cells UMSCC47 and CRISPR/Cas9 system or siRNAs to delete *TRAF3/CYLD.*

### Data Acquisition.

Deidentified publicly available clinical and genomic data from the Cancer Genome Atlas (TCGA) were utilized for this study. In this work, we consider a Gistic score of −2 synonymous with deep deletion. Gistic uses a dynamic segmentation algorithm to define chromosomal arm level (−1) and deeper focal deletions (−2) based on per tumor thresholds (63) Clinical data for the TCGA HNSCC cohort were acquired from Liu et al. (64) Variant calls were downloaded using the R TCGAbiolinks package; (65) calls performed with VarScan (66) were used for all analyses. TCGA RNA sequencing BAMs were downloaded from dbGaP, with NIH request #99293-1 for project #27853.

## Supplementary Material

Appendix 01 (PDF)

Dataset S01 (PDF)

## Data Availability

UNC and E1308 RNAseq data were deposited to dbGaP (dbGaP Study Accession: phs002935.v1.p1, “Transcriptomic Profiling of Oropharyngeal Squamous Cell Carcinoma” (67); and dbGaP Study Accession: phs003320.v1.p1, “RNA sequencing of ECOG-E1308” (68), respectively). All other data are included in the article and/or supporting information.

## References

[1] L. J. Viens , Human papillomavirus-associated cancers–United States, 2008–2012. MMWR Morb. Mortal Wkly. Rep. **65**, 661–666 (2016). 27387669 10.15585/mmwr.mm6526a1

[2] C. H. Shiboski, B. L. Schmidt, R. C. K. Jordan, Tongue and tonsil carcinoma: Increasing trends in the U.S. population ages 20–44 years. Cancer **103**, 1843–1849 (2005). 15772957 10.1002/cncr.20998

[3] J. E. Tota , Evolution of the oropharynx cancer epidemic in the United States: Moderation of increasing incidence in younger individuals and shift in the burden to older individuals. J. Clin. Oncol. **37**, 1538–1546 (2019). 31026209 10.1200/JCO.19.00370 PMC6599405

[4] B. Burtness , Pembrolizumab alone or with chemotherapy versus cetuximab with chemotherapy for recurrent or metastatic squamous cell carcinoma of the head and neck (KEYNOTE-048): A randomised, open-label, phase 3 study. Lancet **394**, 1915–1928 (2019). 31679945 10.1016/S0140-6736(19)32591-7

[5] K. K. Ang , Randomized phase III trial of concurrent accelerated radiation plus cisplatin with or without cetuximab for stage III to IV head and neck carcinoma: RTOG 0522. J. Clin. Oncol. **32**, 2940–2950 (2014). 25154822 10.1200/JCO.2013.53.5633 PMC4162493

[6] C. Fakhry , Human papillomavirus and overall survival after progression of oropharyngeal squamous cell carcinoma. J. Clin. Oncol. **32**, 3365–3373 (2014). 24958820 10.1200/JCO.2014.55.1937 PMC4195851

[7] A. Argiris , Prognostic significance of human papillomavirus in recurrent or metastatic head and neck cancer: An analysis of Eastern Cooperative oncology group trials. Ann. Oncol. **25**, 1410–1416 (2014). 24799460 10.1093/annonc/mdu167 PMC4071756

[8] C. B. Warinner, R. W. Bergmark, R. Sethi, E. M. Rettig, Cancer-related activity limitations among head and neck cancer survivors. Laryngoscope **132**, 593–599 (2022). 34355796 10.1002/lary.29795

[9] S. S. Yom , Reduced-dose radiation therapy for HPV-associated oropharyngeal carcinoma (NRG Oncology HN002). J. Clin. Oncol. **39**, 956–965 (2021). 33507809 10.1200/JCO.20.03128 PMC8078254

[10] S. Marur , E1308: Phase II trial of induction chemotherapy followed by reduced-dose radiation and weekly cetuximab in patients with hpv-associated resectable squamous cell carcinoma of the oropharynx- ecog-acrin cancer research group. J. Clin. Oncol. **35**, 490–497 (2017). 28029303 10.1200/JCO.2016.68.3300 PMC5455313

[11] B. S. Chera , Mature results of a prospective study of deintensified chemoradiotherapy for low-risk human papillomavirus-associated oropharyngeal squamous cell carcinoma. Cancer **124**, 2347–2354 (2018). 29579339 10.1002/cncr.31338

[12] T. P. Schrank , Comprehensive viral genotyping reveals prognostic viral phylogenetic groups in HPV16-Associated squamous cell carcinoma of the oropharynx. Mol. Cancer Res. **20**, 1489–1501 (2022). 35731223 10.1158/1541-7786.MCR-21-0443 PMC11249119

[13] L. Mirabello , HPV16 E7 genetic conservation is critical to carcinogenesis. Cell **170**, 1164–1174.e6 (2017). 28886384 10.1016/j.cell.2017.08.001 PMC5674785

[14] D. Pyeon , Fundamental differences in cell cycle deregulation in human papillomavirus-positive and human papillomavirus-negative head/neck and cervical cancers. Cancer Res. **67**, 4605–4619 (2007). 17510386 10.1158/0008-5472.CAN-06-3619 PMC2858285

[15] N. F. Schlecht , Gene expression profiles in HPV-infected head and neck cancer. J. Pathol. **213**, 283–293 (2007). 17893858 10.1002/path.2227

[16] Cancer Genome Atlas Network, Comprehensive genomic characterization of head and neck squamous cell carcinomas. Nature **517**, 576–582 (2015). 25631445 10.1038/nature14129 PMC4311405

[17] M. Parfenov , Characterization of HPV and host genome interactions in primary head and neck cancers. Proc. Natl. Acad. Sci. U.S.A. **111**, 15544–15549 (2014). 25313082 10.1073/pnas.1416074111 PMC4217452

[18] S. Tilborghs , The role of Nuclear Factor-kappa B signaling in human cervical cancer. Crit. Rev. Oncol. Hematol. **120**, 141–150 (2017). 29198328 10.1016/j.critrevonc.2017.11.001

[19] C. Pan, N. Issaeva, W. G. Yarbrough, HPV-driven oropharyngeal cancer: Current knowledge of molecular biology and mechanisms of carcinogenesis. Cancers Head Neck **3**, 12 (2018). 31093365 10.1186/s41199-018-0039-3 PMC6460765

[20] S. Ren , HPV E2, E4, E5 drive alternative carcinogenic pathways in HPV positive cancers. Oncogene **39**, 6327–6339 (2020). 32848210 10.1038/s41388-020-01431-8 PMC7529583

[21] N. V. Anayannis , Association of an intact E2 gene with higher HPV viral load, higher viral oncogene expression, and improved clinical outcome in HPV16 positive head and neck squamous cell carcinoma. PLoS One **13**, e0191581 (2018). 29451891 10.1371/journal.pone.0191581 PMC5815588

[22] T. P. Schrank , Direct comparison of HPV16 viral genomic integration, copy loss, and structural variants in oropharyngeal and uterine cervical cancers reveal distinct relationships to E2 disruption and somatic alteration. Cancers **14**, 4488 (2022). 36139648 10.3390/cancers14184488 PMC9496734

[23] Z. Wang, M. Gerstein, M. Snyder, RNA-Seq: A revolutionary tool for transcriptomics. Nat. Rev. Genet. **10**, 57–63 (2009). 19015660 10.1038/nrg2484 PMC2949280

[24] L. A. Byers , An epithelial-mesenchymal transition gene signature predicts resistance to EGFR and PI3K inhibitors and identifies Axl as a therapeutic target for overcoming EGFR inhibitor resistance. Clin. Cancer Res. **19**, 279–290 (2013). 23091115 10.1158/1078-0432.CCR-12-1558 PMC3567921

[25] Q.-W. Wang, W.-W. Lin, Y.-J. Zhu, Comprehensive analysis of a TNF family based-signature in diffuse gliomas with regard to prognosis and immune significance. Cell Commun. Signal **20**, 6 (2022). 35000592 10.1186/s12964-021-00814-y PMC8744324

[26] E. R. Malone, M. Oliva, P. J. B. Sabatini, T. L. Stockley, L. L. Siu, Molecular profiling for precision cancer therapies. Genome Med. **12**, 8 (2020). 31937368 10.1186/s13073-019-0703-1 PMC6961404

[27] S. C. Winter , Relation of a hypoxia metagene derived from head and neck cancer to prognosis of multiple cancers. Cancer Res. **67**, 3441–3449 (2007). 17409455 10.1158/0008-5472.CAN-06-3322

[28] M. Rasmussen , RNA profiles reveal signatures of future health and disease in pregnancy. Nature **601**, 422–427 (2022). 34987224 10.1038/s41586-021-04249-w PMC8770117

[29] J. S. Parker , Supervised risk predictor of breast cancer based on intrinsic subtypes. J. Clin. Oncol. **27**, 1160–1167 (2009). 19204204 10.1200/JCO.2008.18.1370 PMC2667820

[30] R. Liu , The prognostic role of a gene signature from tumorigenic breast-cancer cells. New Engl. J. Med. **356**, 217–226 (2007). 17229949 10.1056/NEJMoa063994

[31] A. Li , Unsupervised analysis of transcriptomic profiles reveals six glioma subtypes. Cancer Res. **69**, 2091–2099 (2009). 19244127 10.1158/0008-5472.CAN-08-2100 PMC2845963

[32] L. Spinelli, S. Carpentier, F. Montañana Sanchis, M. Dalod, T.-P. Vu Manh, BubbleGUM: Automatic extraction of phenotype molecular signatures and comprehensive visualization of multiple gene set enrichment analyses. BMC Genom. **16**, 814 (2015). 10.1186/s12864-015-2012-4 PMC4617899 26481321

[33] A. Subramanian , Gene set enrichment analysis: A knowledge-based approach for interpreting genome-wide expression profiles. Proc. Natl. Acad. Sci. U.S.A. **102**, 15545–15550 (2005). 16199517 10.1073/pnas.0506580102 PMC1239896

[34] Z. Xie , Gene set knowledge discovery with enrichr. Curr. Protoc. **1**, e90 (2021). 33780170 10.1002/cpz1.90 PMC8152575

[35] D. Venet, J. E. Dumont, V. Detours, Most random gene expression signatures are significantly associated with breast cancer outcome. PLOS Comput. Biol. **7**, e1002240 (2011). 22028643 10.1371/journal.pcbi.1002240 PMC3197658

[36] A. Dhawan , Guidelines for using sigQC for systematic evaluation of gene signatures. Nat. Protoc. **14**, 1377–1400 (2019). 30971781 10.1038/s41596-019-0136-8

[37] X. Liu , A prognostic gene expression signature for oropharyngeal squamous cell carcinoma. EBioMedicine **61**, 102805 (2020). 33038770 10.1016/j.ebiom.2020.102805 PMC7648117

[38] P. Langfelder, S. Horvath, WGCNA: An R package for weighted correlation network analysis. BMC Bioinf. **9**, 559 (2008). 10.1186/1471-2105-9-559 PMC2631488 19114008

[39] J. Ameijeiras-Alonso, R. M. Crujeiras, A. Rodríguez-Casal, Mode testing, critical bandwidth and excess mass. TEST **28**, 900–919 (2019).

[40] B. A. Luca , Atlas of clinically distinct cell states and ecosystems across human solid tumors. Cell **184**, 5482–5496.e28 (2021). 34597583 10.1016/j.cell.2021.09.014 PMC8526411

[41] M. B. Koenigs , Association of estrogen receptor alpha expression with survival in oropharyngeal cancer following chemoradiation therapy. J. Natl. Cancer Inst. **111**, 933–942 (2019). 30715409 10.1093/jnci/djy224 PMC6748818

[42] M. Deng, J. Brägelmann, I. Kryukov, N. Saraiva-Agostinho, S. Perner, FirebrowseR: An R client to the Broad Institute’s Firehose Pipeline. Database (Oxford) **2017**, baw160 (2017). 28062517 10.1093/database/baw160 PMC5216271

[43] E. A. Williams , CYLD mutation characterizes a subset of HPV-positive head and neck squamous cell carcinomas with distinctive genomics and frequent cylindroma-like histologic features. Mod. Pathol. **34**, 358–370 (2021). 32892208 10.1038/s41379-020-00672-y PMC7817524

[44] M. Hajek , TRAF3/CYLD mutations identify a distinct subset of human papillomavirus-associated head and neck squamous cell carcinoma. Cancer **123**, 1778–1790 (2017). 28295222 10.1002/cncr.30570 PMC5419871

[45] L. A. Koneva , HPV integration in HNSCC correlates with survival outcomes, immune response signatures, and candidate drivers. Mol. Cancer Res. **16**, 90–102 (2018). 28928286 10.1158/1541-7786.MCR-17-0153 PMC5752568

[46] A. V. Gudkov, E. A. Komarova, p53 and the carcinogenicity of chronic inflammation. Cold Spring Harb. Perspect. Med. **6**, a026161 (2016). 27549311 10.1101/cshperspect.a026161 PMC5088512

[47] X. Jin , Phosphorylated RB promotes cancer immunity by inhibiting NF-κB activation and PD-L1 expression. Mol. Cell **73**, 22–35.e6 (2019). 30527665 10.1016/j.molcel.2018.10.034 PMC8968458

[48] D. L. Faden , APOBEC mutagenesis is concordant between tumor and viral genomes in HPV-Positive head and neck squamous cell carcinoma. Viruses **13**, 1666 (2021). 34452530 10.3390/v13081666 PMC8402723

[49] V. L. Cannataro , APOBEC-induced mutations and their cancer effect size in head and neck squamous cell carcinoma. Oncogene **38**, 3475–3487 (2019). 30647454 10.1038/s41388-018-0657-6 PMC6499643

[50] S. Henderson, A. Chakravarthy, X. Su, C. Boshoff, T. R. Fenton, APOBEC-mediated cytosine deamination links PIK3CA helical domain mutations to human papillomavirus-driven tumor development. Cell Rep. **7**, 1833–1841 (2014). 24910434 10.1016/j.celrep.2014.05.012

[51] S. Henderson, A. Chakravarthy, T. Fenton, When defense turns into attack: Antiviral cytidine deaminases linked to somatic mutagenesis in HPV-associated cancer. Mol. Cell Oncol. **1**, e29914 (2014). 27308321 10.4161/mco.29914 PMC4905184

[52] B. T. Beaty , PIK3CA mutation in HPV-associated OPSCC patients receiving deintensified chemoradiation. J. Natl. Cancer Inst. **112**, 855–858 (2019), 10.1093/jnci/djz224. 31747025

[53] W. Dai , Clinical outcome-related mutational signatures identified by integrative genomic analysis in nasopharyngeal carcinoma. Clin. Cancer Res. **26**, 6494–6504 (2020). 32988965 10.1158/1078-0432.CCR-20-2854

[54] R. You , Clonal mutations activate the NF-κB pathway to promote recurrence of nasopharyngeal carcinoma. Cancer Res. **79**, 5930–5943 (2019). 31484669 10.1158/0008-5472.CAN-18-3845

[55] M. H. Park, J. T. Hong, Roles of NF-κB in cancer and inflammatory diseases and their therapeutic approaches. Cells **5**, 15 (2016). 27043634 10.3390/cells5020015 PMC4931664

[56] T. P. Schrank , NF-kB over-activation portends improved outcomes in HPV-associated head and neck cancer. Oncotarget **13**, 707–722 (2022). 35634245 10.18632/oncotarget.28232 PMC9131933

[57] I. Carrero, H.-C. Liu, A. G. Sikora, A. Milosavljevic, Histoepigenetic analysis of HPV- and tobacco-associated head and neck cancer identifies both subtype-specific and common therapeutic targets despite divergent microenvironments. Oncogene **38**, 3551–3568 (2019). 30655605 10.1038/s41388-018-0659-4 PMC6756123

[58] N. Kourtis , A single-cell map of dynamic chromatin landscapes of immune cells in renal cell carcinoma. Nat. Cancer **3**, 885–898 (2022), 10.1038/s43018-022-00391-0. 35668194 PMC9325682

[59] F. Ferrandino , Notch and NF-κB: Coach and players of regulatory T-cell response in cancer. Front. Immunol. **9**, 2165 (2018). 30364244 10.3389/fimmu.2018.02165 PMC6193072

[60] P. Yu, Y.-X. Fu, Tumor-infiltrating T lymphocytes: Friends or foes? Lab. Invest. **86**, 231–245 (2006). 16446705 10.1038/labinvest.3700389

[61] B. S. Chera , Rapid clearance profile of plasma circulating tumor HPV type 16 DNA during Chemoradiotherapy correlates with disease control in HPV-associated oropharyngeal cancer. Clin. Cancer Res. **25**, 4682–4690 (2019). 31088830 10.1158/1078-0432.CCR-19-0211 PMC6679766

[62] H. M. Walline , Genomic integration of high-risk HPV alters gene expression in oropharyngeal squamous cell carcinoma. Mol. Cancer Res. **14**, 941–952 (2016). 27422711 10.1158/1541-7786.MCR-16-0105 PMC5065754

[63] C. H. Mermel , GISTIC2.0 facilitates sensitive and confident localization of the targets of focal somatic copy-number alteration in human cancers. Genome Biol. **12**, R41 (2011). 21527027 10.1186/gb-2011-12-4-r41 PMC3218867

[64] J. Liu , An integrated TCGA pan-cancer clinical data resource to drive high-quality survival outcome analytics. Cell **173**, 400–416.e11 (2018). 29625055 10.1016/j.cell.2018.02.052 PMC6066282

[65] M. Mounir , New functionalities in the TCGAbiolinks package for the study and integration of cancer data from GDC and GTEx. PLoS Comput. Biol. **15**, e1006701 (2019). 30835723 10.1371/journal.pcbi.1006701 PMC6420023

[66] D. C. Koboldt , VarScan 2: Somatic mutation and copy number alteration discovery in cancer by exome sequencing. Genome Res. **22**, 568–576 (2012). 22300766 10.1101/gr.129684.111 PMC3290792

[67] T. P. Schrank, Transcriptomic Profiling of Oropharyngeal Squamous Cell Carcinoma. dbGaP. https://www.ncbi.nlm.nih.gov/projects/gap/cgi-bin/study.cgi?study_id=phs002935.v1.p1. Accessed 2 May 2023.

[68] T. P. Schrank, RNA Sequencing of ECOG-E1308. dbGaP. https://www.ncbi.nlm.nih.gov/projects/gap/cgi-bin/study.cgi?study_id=phs003320.v1.p1. Accessed 3 July 2023.

