# Supplementary material for: Noncanonical HPV carcinogenesis drives radiosensitization of head and neck tumors
Source: Proc Natl Acad Sci U S A. 2023 Jul 31;120(32):e2216532120. doi: 10.1073/pnas.2216532120 (PMC10410762; doi:10.1073/pnas.2216532120)
Supplement: Supplementary file 1 — Appendix 01 (PDF) [file pnas.2216532120.sapp.pdf]

PNAS

Supporting Information for

Non-canonical HPV carcinogenesis drives radio-sensitization of head and neck tumors

Travis P. Schrank[1,2,†], Aditi Kothari[1,2,†], William H. Weir[1], Wesley H. Stepp[1], Hina Rehmani[1,2], Xinyi Liu[3,4], Xiaowei Wang[3,4], Andrew Sewell[1], Xue Li[1], Jason Tasoulas[1], Sulgi Kim[1], Gray Yarbrough[1], Yue Xie[5], Yael Flamand[5], Shanthi Marur[6], Michele C. Hayward[2], Di Wu[7,8], Barbara Burtness[9], Karen S. Anderson[10,11], Albert S. Baldwin[2,12], Wendell G. Yarbrough[1,2,12] and Natalia Issaeva[1,2,12]

Correspondence to: Travis P. Schrank, Wendell G. Yarbrough, Natalia Issaeva.
Email: travis_schrank@med.unc.edu, dell@med.unc.edu, natalia.isaeva@med.unc.edu

This PDF file includes:

Supporting text (SI Materials and Methods)
Figures S1 to S6
Tables S1 to S5
SI References

Supporting Information Text

SI Materials and Methods. ***RNA Isolation, Library Preparation, and Sequencing (UNC and E1308 cohorts) -*** Formalin-fixed paraffin-embedded (FFPE) tissue samples were sent to the UNC Lineberger Comprehensive Cancer Center (LCCC) Translational Genomics Lab (TGL) for RNA isolation using the Maxwell 16 MDx Instrument (Promega AS3000) and the Maxwell 16 LEV RNA FFPE Kit (Promega AS1260) following the manufacturer's protocol (Promega 9FB167). After a pathology review of a hematoxylin and eosin (H&E) stained slide to identify tumor area, RNA was extracted from unstained slides using macro-dissection. Total RNA quality was measured using a NanoDrop spectrophotometer (Thermo Scientific ND-2000C) and a TapeStation 4200 (Agilent G2991AA). Total RNA concentration was quantified using a Qubit 3.0 fluorometer (Life Technologies Q33216). Libraries were prepared with Illumina TruSeq Stranded Total RNA with Ribo-Zero protocol. Libraries were sequenced on an Illumina HiSeq2500 sequencer. Paired end read data, with read lengths of 75 were collected. Archival tissue was sequenced and corresponding chart review performed after IRB approval was obtained: UNC IRB 17-2947 (UNC cohort), UNC IRB 21-1855 (E1308).

Cohort Selection and Inclusion Criteria - TCGA: Tumors with *TP53* mutations or deep deletions were excluded from the analysis. To determine HPV16 status, viral gene expression was also quantified using Salmon(1) and the HPV16 A1 genotype, RefSeq NC_001526.4. HPV16 reads demonstrated an obviously bimodal distribution and visual thresholding was performed to select HPV positive tumors. Firehose clinical annotations to the TCGA data were used to assign tumor subsite.(2) Anatomic subsites from the oropharynx, tonsil, and base of tongue were included; as well, nearby subsites of the hypopharynx and oral tongue were included if the tumor was HPV+ and *TP53* wild-type, as these tumors were considered of oropharyngeal origin. HPV+ tumors from more distal sites (e.g., larynx, alveolar ridge, maxilla) were excluded. A total of 61 patients met these criteria. ***Vanderbilt:*** No DNA sequencing data were available so *TP53* mutations could not be assessed. Raw reads were not available, and therefore published HPV status annotations assigned based on published analysis of RNA reads were applied.(3) Only tumors from the anatomic oropharynx (tonsil and tongue base) were included based on the published clinical annotations.(3) A total of 93 cases met these criteria. ***UNC:*** No DNA sequencing data were available so *TP53* mutations could not be assessed. To determine HPV16 status, viral genes expression was also quantified using Salmon(1) and the HPV16 A1 genotype, RefSeq NC_001526.4. HPV16 reads demonstrated an obviously bimodal distribution and visual thresholding was performed to select HPV positive tumors. Only tumors with primaries in anatomic oropharynx (tonsil or base of tongue) were included. A total of 104 cases met these criteria.

Preprocessing of Gene Expression Data - Starting with TCGA data, raw per-gene RNA read counts were preprocessed by filtering low expression genes to obtain an approximately Gaussian distribution of Log_2CPM values. Genes passing this filtering step were excluded if quantification was not available also in the UNC and Vanderbilt cohorts. RNA sequencing of the UNC cohort was per-gene quantified with Salmon. Publicly available TPM quantified data was used for the Vanderbilt cohort.(3) Log transformed distributions of transcripts per million (TPM) of these genes for both the UNC and Vanderbilt cohorts were found to be approximately gaussian. Filtered data were then normalized using the trimmed means of M values methods provided in the R edgeR package.(4)

Weighted Gene Correlation Network Analysis (WGCNA) - To identify potential biologically relevant autocorrelated gene sets or gene expression modules, the WGCNA algorithm was applied to the above-described normalized RNA expression data.(5) Default parameters according to recommendations from the WGCNA package authors were used unless otherwise noted.(5) The soft threshold network was constructed calculating a scale-free topology fit index for powers ranging from 4-20. The final scale-free network was constructed with soft power set to 6. This identical procedure was performed on the three datasets, independently.

Clinical Factors and Survival Analysis - Survival statistics were generated with the R survival package (v3.2-7) and visualized with the R survminer package (0.4.8). p-values represent log-rank test. Proportional hazard assumption was verified with the cox.zph() R function.

Hypergeometric Gene Set Enrichment Analysis - Hypergeometric (gene ontology) enrichment analysis was performed for the derived consensus modules using the EnrichR package with default parameters.(6) All results were corrected for multiple comparisons by the EnrichR pipeline, and adjusted p-values were considered significant if adjusted $p < 0.05$. Annotation gene sets were downloaded from MSigDB.(7)

Tumor Microenvironment Analysis – Tumor microenvironment decomposition of each sample included in the study from all three cohorts was performed with the Ecotyper pipeline, which is a non-negative matrix factorization approach trained on single cell RNA sequencing data.(8) The Ecotyper carcinoma pipeline was run in “recovery mode” with default settings on unfiltered TPM data based on recommendations from the authors. Cell types well quantified as the sum of their substate intensities. Cell types were quantified as a log fraction, log2((microenvironment cell)/(epithelial cell)).

Evaluating the TCGA Mutational Landscape - The R maftools package was used to summarize and process variant calls from the Varscan pipeline, downloaded using the R TCGA biolinks.(9) Copy number calls (Gistic) were obtained via the Broad Firehose Portal. Focal deletions (Gistic -2) and focal amplifications (+2) were considered.(2, 10) Mutation frequency per tumor was based on the number of variant calls per tumor based on the Varscan pipeline.(11)

APOBEC and Mutational Signature Analysis - The maftools function trinucleotideMatrix() was used to score tumors for the proportion of SNPs potentially related to APOBEC.(9) Nonnegative matrix factorization was used to estimate the contribution of different mutational process influencing somatic alteration, based on mutational contexts as define by the COSMIC mutational signatures, Version 2.(12) This analysis was implemented and visualized with the R package DeconstructSigs, after generating trinucleotide context SNP matrices with in-house scripts.(13)

RNA Based Genomic Copy Number Analysis - Per gene raw read count data for the UNC cohort were extracted from the Salmon output, and was formatted for use with the CNVkit package per the authors’ instructions.(14) The CNVkit RNA pipeline was run in reference free mode with default settings.(14) Log2ratio values < -0.35 were considered to have low level copy loss.

Viral Gene Expression Integration Analysis - For datasets with available raw reads (TCGA and UNC), both viral and human transcripts were quantified using Salmon using HG38 and HPV16-A1, RefSeq NC_001526.4. Viral gene expression was quantified as the log2(1+counts per million reads) including all HPV16 and human transcripts. Outlier low viral expression values were trimmed to the minima of the normally distributed values for visualization and clustering analyses. Discordant human-viral split read pairs were quantified using the ViFi pipeline.(15) An empiric threshold of 25 discordant read pairs was applied as this threshold was highly related to an increased (relative) expression of viral oncogenes *HPV E6* and *E7*, however our downstream results were not sensitive to this threshold.

Methylation Tumor Subclass Analysis – Illumina 450K methylation array data for the HNSC-TCGA were downloaded from the Genomic Data Commons (https://gdc.cancer.gov/) using the TCGAWorkflowData package in R.(16) Analyses were conducted using the level 3, normalized beta-values. For clustering analysis, we selected the 2,000 probes with largest standard deviations in (normalized) beta value (top 50%) when considering only HPV+ HNSCC cases. Consensus clustering was then applied with 1000 replicates. Considering the small number of HPV+ HNSCC cases (n=61), we did not investigate more than 2 methylation clusters. For gene based analyses, a high quality set of probes was retained as detailed by Zhou et al.(17) with removal of non-detected probes, probes associated with polymorphisms, and those on the X/Y chromosomes, resulting in ~310,000 sites. These were mapped to genes using annotations from

Zhou et al.(17) Expression data was filtered and normalized as described above. Significance of expression-methylation correlation for each gene-probe pair was assessed using a linear model. Gene-level significance was assessed using the Robust Rank Aggregation approach proposed Ren et al. in order to account for the bias introduced by the number of probes per gene.(18) In short, for each gene the minimum order statistic is calculated for all associated probes under the assumption that these are drawn uniformly from [0,1] (i.e. no methylation-expression correlation is present).(18) Multiple testing correction is applied individually to each gene-level p-value using Bonferroni correction based on the number of probes, and additionally across all genes using the Benjamini-Hochberg approach. For each gene signature, we assess strength of methylation regulation by comparing the observed percentage of significant genes ($q < 0.05$) to randomly sampled groups of the same size. We also compute the GSEA enrichment score for each cluster (Figure 6H) as detailed in Ren at al.(18)

Immunohistochemical Studies – IF staining was performed at UNC Pathology Services Core. High resolution acquisition of IF slides was performed with the Aperio Versa 200 scanner (Leica Biosystems Inc.) at an apparent magnification of 10X. Images were uploaded to the eSlideManager database (Aperio; eSlideManager version 12.3.3.7075) at the Pathology Services Core at UNC. Regions of interest were manually annotated using Tissue Studio. For analysis we used Definiens Architect XD 2.7.0 Build 60765 x64 (Definiens AG, Munich, Germany) with Tissue Studio version 4.4.2 (IF portal) using the co-expression algorithm. The software can identify regions with co-expression of multiple markers. CD8 was stained with Cell marque Cat# 108R-14, CD4 stained with Cell Marque Cat# 104R-24, Abcam FoxP3 Cat# ab20034 and cytokeratin with Leica Cat# NCL-L-AE1/AE3-601. Hoeschst was used for nuclear staining.

Establishing of TRAF3 knockout cells - UMSCC47 cells were tested mycoplasma negative by MycoAlert Mycoplasma Detection Kit (Lonza) and were cultured in Dulbecco's modified Eagle's medium (DMEM) (Genesee #25-501N) supplemented with 10% FBS (Genesee # 25-514H), 1% penicillin-streptomycin (Genesee #25-512), 1% non-essential amino acids (Genesee #25-536), and 1% L-glutamine (Genesee #25-509) in a humidified atmosphere of 5% CO2 at 37°C.

Co-transfection of TRAF3 CRISPR/Cas KO (Santa Cruz # sc-400473-KO-2) and TRAF3 HDR (Santa Cruz # sc-400473-HDR-2) plasmids with Lipofectamine 2000 (Thermofisher # 11668019) was used per manufacture's protocol to establish TRAF3 knockout UMSCC47 cells. TRAF3 KO clones were selected with 2µg/ml puromycin (InvivoGen ant-pr-1).

siRNAs transfection – was performed using Lipofectamine™ RNAiMAX Transfection Reagent (ThermoFisher # 13778150) according to manufacture instructions. We used Horizon discovery/Dharmacon L-005252-00-0005 ON-TARGETplus siRNA SMARTpool for TRAF3, Horizon discovery/Dharmacon L-004609-00-0005 ON-TARGETplus siRNA SMARTpool for CYLD, and Horizon discovery/Dharmacon ON-TARGETplus Non-targeting siRNAs (D-001210-01-05).

Immunoblotting - Immunoblotting was performed as previously described (19). Briefly, cells were harvested by trypsinization (0.25% Trypsin, Genesee # 25-510) and lysed on ice in radioimmunoprecipitation assay (Chen et al.) lysis buffer (Sigma # R0278) with the addition of protease and phosphatase inhibitors (Thermofisher # 78442) for 15 min. Insoluble material was removed by centrifugation at 14,000 rpm for 15 min at 4^0 C. 40µg of cell lysates were mixed with 2X loading Laemmli buffer (Biorad) supplemented with DTT (Sigma) and incubated for 7 min at 95°C. Proteins were separated in Tris-glycine polyacrylamide gels (Mini-PROTEAN; Bio-Rad) and electrophoretically transferred onto polyvinylidene fluoride membranes. Membranes were blocked with 3% BSA in PBS and incubated with antibodies against TRAF3 (Santa Cruz # 1828) and GPDH (Cell Signaling # 14C10). After incubation with primary antibodies, membranes were washed in TBS-T, incubated with secondary goat anti-rabbit IgG (H+L) DyLight 650 (Thermofisher), and signals were visualized using a Bio-Rad imager.

Clonogenic Survival Assay- Cells were plated into six well plates at a density of 1000 cells/well. The next day, the cells were irradiated in the RS 2000 Small Animal Irradiator (Rad Source). 10

days after irradiation, colonies were fixed and stained with methylene blue in methanol (4 g/L). Stained area was quantified using ImageJ.

RT-qPCR - Total RNA was extracted from cells with the Total RNA Extraction & Purification kit (NEB) and cDNA was synthesized using Verso cDNA Synthesis Kit (ThermoFisher # AB1453B) according to the manufacturer's instructions. Quantitative RT-PCR was performed using Power SYBR™ Green PCR Master Mix (Thermofisher # 4367659) and primer pairs specific to *TRAF3*, *CYLD*, or GPDH in the QuantStudio Real-Time PCR system (Thermofisher). RT-qPCR reactions were done in duplicate at least, and the ΔΔCt method was used to analyze the data; relative expression (% from GPDH) is presented.

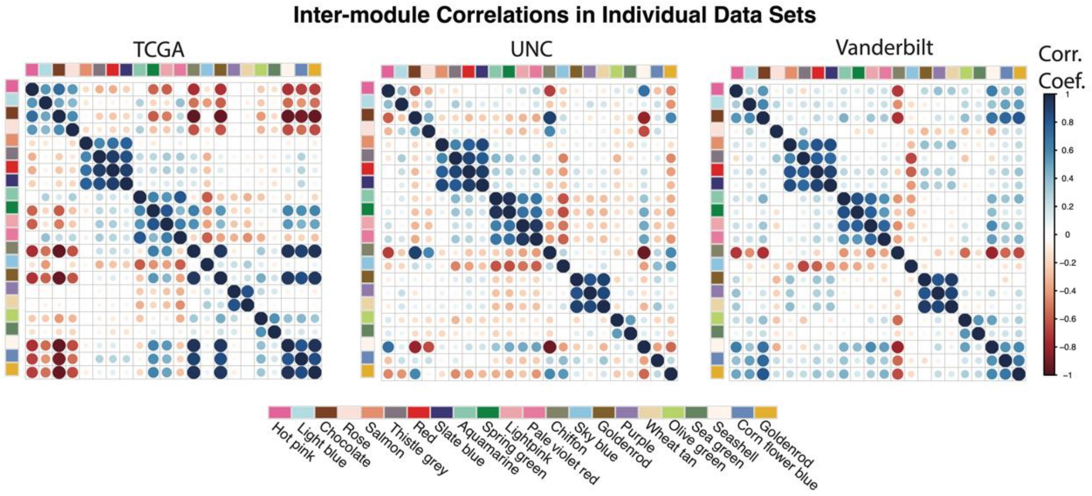

Figure S1. WGCNA of multiple cohorts reveals consensus transcriptional modules in HPV+ HNSCC. Inter-module correlation in individual datasets. Spearman correlation coefficients are displayed for per-tumor module PCA scores within each dataset. Polarity of PCA score values was assigned so that higher scores reflect higher median gene expression for all analyses displayed.

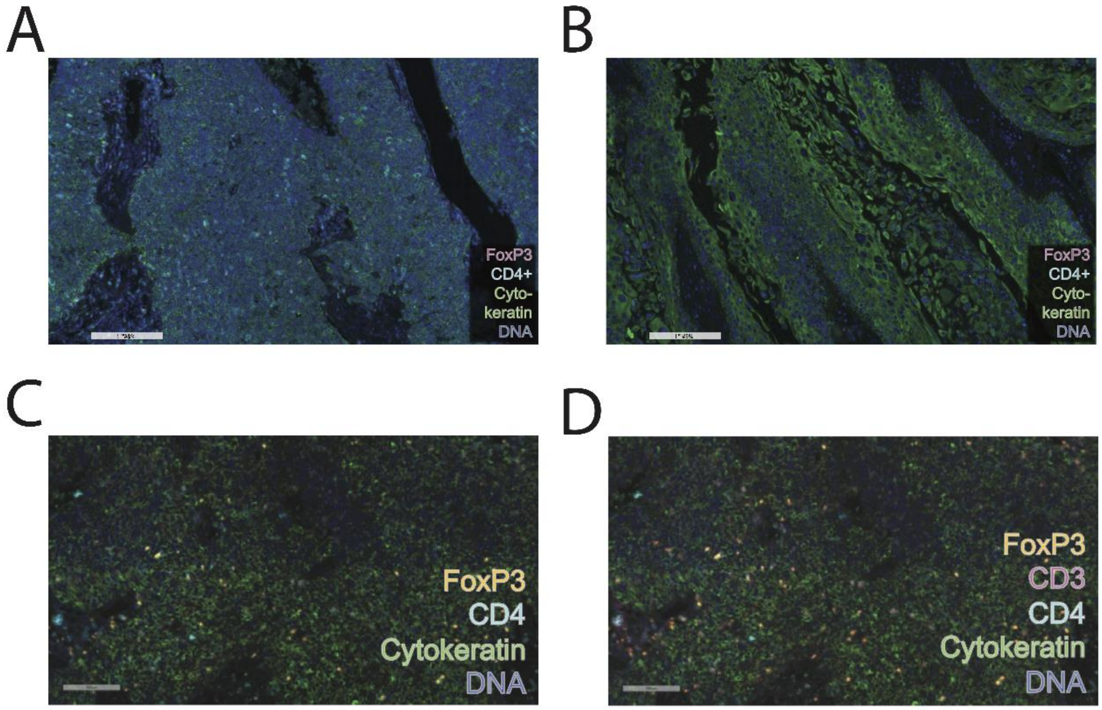

Figure S2. Representative Images of immunofluorescent staining of HPV+ HNSCCs from the UNC cohort. A. NF-κB high tumor. B. NF-κB low tumor. C-D. Co-localization of FoxP3 and CD3 in a NF-κB high tumor.

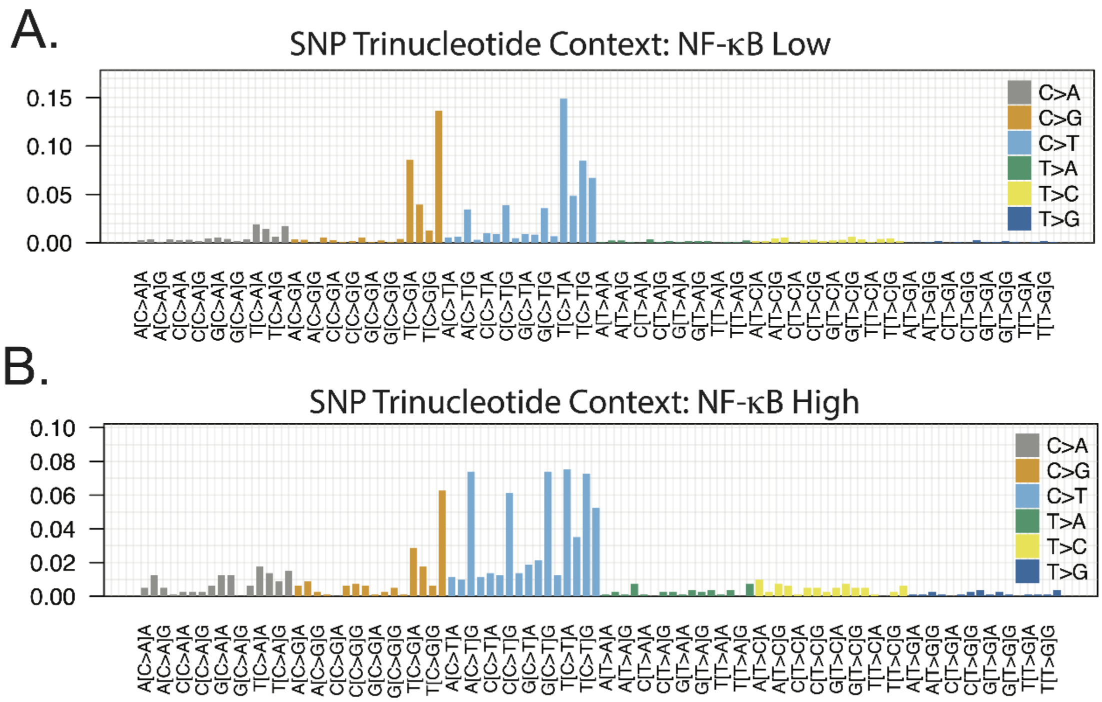

Figure S3. Genotypic associations with the sky blue (NF-κB) module. A-B. Trinucleotide cotexts of SNPs identified in sky blue (NF-κB) module low vs. high expression tumors. TCGA data.

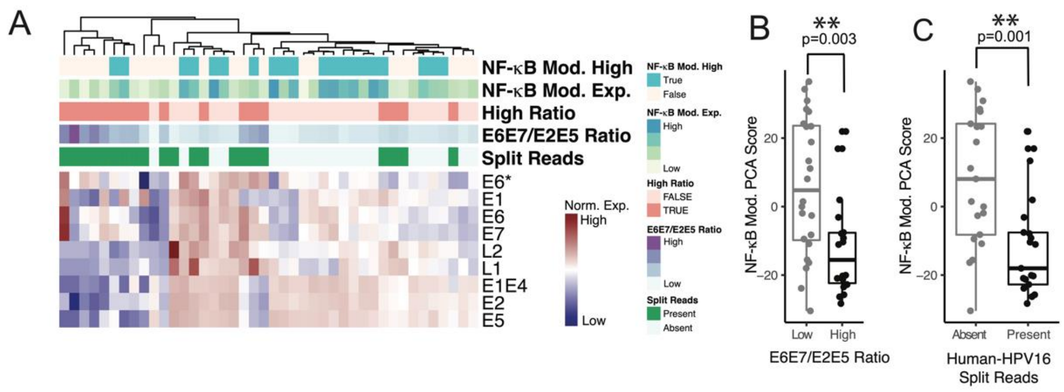

Figure S4. NF-κB Module expression is related to patterns of viral gene expression and viral genomic integration: Analysis of TCGA data. A-C. Analysis of RNAseq data from the UNC Cohort. **A.** Annotated heat map of HPV16 viral gene expression. Columns – tumor samples, organized by clustering on viral gene expression, normalized to human RNA. Sky blue (NF-κB) Module High – Tumors with high expression of the sky blue (NF-κB) module genes based on PCA score. Sky blue (NF-κB) Module Expression – Principal component analysis score for the NF-κB (Sky Blue) module. E6E7/E2E5 Ratio – Ratio of E6 and E7 expression to E2 and E5 expression. Log2[(readsE6+readsE7) / (readsE2+readsE5)]. Split Reads – Presence of detectable split read-pairs mapping to both the HPV16 and human genome, as identified by the ViFi viral integration software package. **B.** Box plot of sky blue (NF-κB) Module PCA scores for tumors with low and high E6E7/E2E5 ratio, as defined in Panel A. Significance based on Wilcoxon Rank-sum test. **C.** Box plot of Sky blue (NF-κB) Module PCA scores for tumors with and without viral integration, as defined by split read-pairs as in Panel A. Significance based on Wilcoxon Rank-sum test.* P-value < 5*10^-2. ** P-value < 5*10^-3

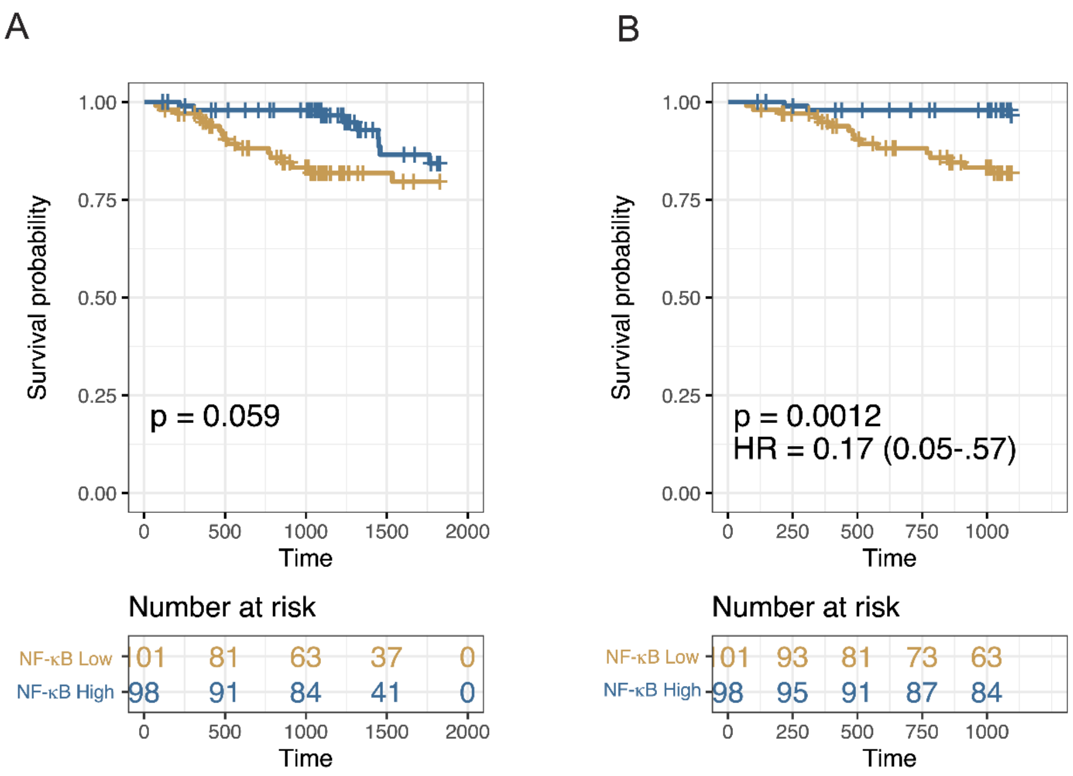

Figure S5. Overall survival (OS) and expression of sky blue (NF-κB) module. Kaplan-Meier plots with number at risk tables below. A. All patients with OS and and quantitative smoking data. Hazard ratio confidence intervals in parentheses represent 95% CI. P-values represent Log-Rank test. Follow-up was limited to 5-years. B. As in A, but follow-up was limited to 3-years.

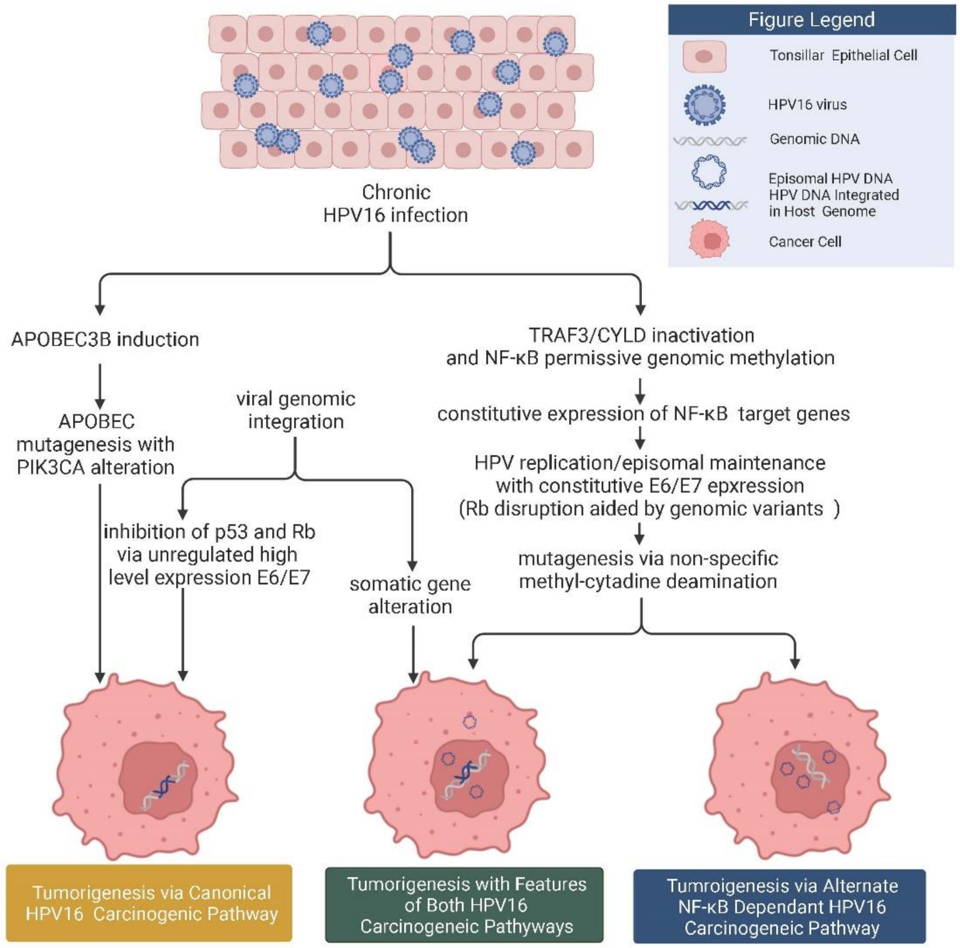

Figure S6. Illustration of alternative vs classical HPV-driven carcinogenesis in HPV+ HNSCC.

Table S1. Module quality metrics

Module name	Module size (n genes)	TCGA: p-value mode test	TCGA: PC1 variance	TCGA: PC2 variance	TCGA: PC1 vs. Median Corr. Spear. P-value	TCGA: PC1 vs. Median Corr. Spear. Rho	UNC: p-value mode test	UNC: PC1 variance	UNC: PC2 variance	UNC: PC1 vs. Median Corr. Spear. P-value	UNC: PC1 vs. Median Corr. Spear. Rho	Vanderbilt: p-value mode test	Vanderbilt: PC1 variance	Vanderbilt: PC2 variance	Vanderbilt: PC1 vs. Median Corr. Spear. P-value	Vanderbilt: PC1 vs. Median Corr. Spear. Rho
aquamarine2	17	0.324	60.15	11.56	0	0.932258065	0.52	68.1	8.84	0	0.951431749	0.03	47.01	14.05	0	0.944809167
chocolate4	205	0.456	20.59	9.02	4.27E-06	0.558963511	0.01	30.74	8.8	0.00096681	0.313094245	0.426	12.61	5.66	2.11E-07	0.514040166
cornflowerblue	165	0.352	19.08	7.28	5.04E-07	0.603014278	0.93	11.09	8.17	3.59E-05	0.387711982	0.248	8.44	6.53	0.776738709	0.029751425
darkgoldenrod4	278	0.29	33.39	7.34	0	0.916604971	0.132	22.68	9.21	1.40E-05	0.406208878	0.664	21.27	8.4	0.025105397	0.232535585
darkolivegreen2	243	0.874	53.37	5.25	0	0.986144897	0.696	51.92	6.23	0	0.983736447	0.854	35.41	4.16	0	0.928709976
darkseagreen4	81	0	82.86	2.84	0	0.978794289	0.112	79.52	4.52	0	0.978111389	0	46.01	5.21	5.13E-36	0.907368599
goldenrod2	158	0.172	23.76	9.21	0.000242446	0.457800106	0.798	19.75	8.84	0.016083415	0.230469836	0.028	15.41	8.75	0.21425192	0.129882725
hotpink2	82	0.544	76.1	4.79	0	0.993918562	0.06	70.51	4.86	0	0.987369104	0.574	58.38	8.13	0	0.985318254
lemonchiffon4	306	0.462	23.59	10.82	0.148038708	0.187308302	0.24	34.03	9.69	2.08E-05	0.398563618	0.148	30.75	7.9	0	0.813508997
lightblue2	5	0.522	71.28	14.3	0	0.954997356	0.96	70.12	12.43	0	0.91225095	0	57.9	21.26	0	0.812434723
lightpink1	33	0.992	64.56	6.54	0	0.983024855	0.79	60.19	8.77	0	0.964127514	0.902	52.99	7.61	0	0.982155113
lightsalmon2	64	0.47	57.61	7	0	0.981068218	0.12	60.66	9.33	0	0.97285701	0.91	46.86	6.09	0	0.969487631
mediumpurple1	71	0.86	57.98	6.28	0	0.960814384	0.102	48.48	6.58	0	0.954369382	0.51	30.9	8.38	0	0.884530453
mistyrose	38	0.894	44.7	12.16	0	0.935695399	0	62.37	6.69	0	0.921508665	0.382	37.14	9.84	0	0.951926233
palevioletred1	17	0.174	65.03	7.59	0	0.947858276	0.3	62.63	8.48	0	0.872189788	0.018	55.73	13.83	0	0.872355346
red	27	0.112	58.23	9.09	0	0.9566367	0.286	62.83	9.42	0	0.963219349	0.364	54.11	8.94	0	0.948882457
seashell1	181	0.314	41.48	6.75	2.56E-06	0.569962983	0.166	43.09	5.89	0	0.95520341	0.502	32.78	8.32	0	0.944197428
skyblue1	203	0.038	51.17	5.07	0	0.976150185	0	52.5	4.53	0	0.975349829	0.012	37.98	3.94	0	0.920712602
slateblue4	9	0.362	61.85	13.14	0	0.92755156	0.248	67.21	12.49	0	0.865508294	0.408	54.98	19.94	0	0.832547522
springgreen4	38	0.352	66.04	8.69	0	0.968217874	0.156	68.12	6.56	0	0.982985822	0.34	60.43	7.24	0	0.968219391
thistle4	65	0.13	52.65	8.43	0	0.954151243	0.526	50.31	7.68	0	0.965851172	0.258	44.74	7.37	0	0.969413029
wheat2	464	0.832	57.34	8.72	0	0.980010576	0.874	61.55	5.86	0	0.987137429	0.472	39.33	5.25	0	0.961251529

Table S2. UNC Clinical Cohort Demographics

	Low NF-κB	High NF-κB	*p-value*
**Total Patients**	42	47	
**Age, mean (SD)**	55.3 (7.7)	57.4 (9.0)	*0.222*
**Female Sex, n (%)**	3 (7.1)	3 (6.4)	*1.000*
**Race, n (%)**			
White	42 (100)	47 (100)	*NA*
Non-white	0 (0)	0 (0)	
**Smoking History, n (%)**			
< 10 pack-years	19 (45.2)	25 (53.2)	*0.591*
< 20 pack-years	24 (57.1)	33 (70.2)	*0.288*
< 30 pack-years	31 (73.8)	37 (78.7)	*0.768*
**TNM Stage (AJCC 7th ed.), n (%)**			
T Low-risk (T1-3)	33 (81)	43 (91.5)	*0.253*
T High-risk (T4)	8 (19)	4 (8.5)	
N Low-risk (N0-2b)	26 (64.3)	27 (57.4)	*0.658*
N High-risk (N2c-3)	15 (35.7)	20 (42.6)	
**T Stage (AJCC 8th ed.), n (%)**			
T1	13 (31.7)	14 (29.8)	*0.446*
T2	15 (36.6)	21 (44.7)	
T3	5 (12.2)	8 (17)	
T4	8 (19.5)	4 (8.5)	
**N Stage (AJCC 8th ed.), n (%)**			
N0	2 (4.9)	3 (6.4)	*0.805*
N1	24 (58.5)	24 (51.1)	
N2	10 (24.4)	11 (23.4)	
N3	5 (12.2)	9 (19.1)	
**Primary Surgery, n (%)**	3 (7.3)	4 (8.5)	*1.000*

*One patient without initial TNM staging available.

Table S3. E1308 Clinical Cohort Demographics

	Low NF-κB	High NF-κB	*p-value*
**Number Total**	26	33	
**Age, mean (SD)**	57.4 (7.3)	57.9 (6.0)	*0.762*
**Female Sex, n (%)**	3 (11.5)	2 (6.1)	*0.780*
**Race, n (%)**			*0.508*
White	25 (96.2)	29 (87.9)	
Non-white	1 (3.8)	4 (12.1)	
**Smoking History, n (%)**			
< 10 pack-years	9 (34.6)	25 (75.8)	***0.004***
< 20 pack-years	14 (53.8)	26 (78.8)	*0.079*
< 30 pack-years	20 (76.9)	30 (90.9)	*0.263*
**TNM Stage (AJCC 7th ed.), n (%)**			
T Low-risk (T1-3)	21 (80.8)	32 (97)	*0.107*
T High-risk (T4)	5 (19.2)	1 (3)	
N Low-risk (N0-2b)	15 (57.7)	24 (72.7)	*0.350*
N High-risk (N2c-3)	11 (42.3)	9 (27.3)	
**T Stage (AJCC 8th ed.), n (%)**			
T1	4 (15.4)	5 (15.2)	*0.232*
T2	13 (50)	21 (63.6)	
T3	4 (15.4)	6 (18.2)	
T4	5 (19.2)	1 (3)	
**N Stage (AJCC 8th ed.), n (%)**			
N0	0 (0)	1 (3)	*0.353*
N1	15 (57.7)	23 (69.7)	
N2	11 (42.3)	9 (27.3)	
**Complete Response, n (%)**	17 (65.4)	16 (48.5)	*0.301*

Table S4. Combined E1308, TCGA, UNC Clinical Demographics – Non-Surgical Management

	Low NF-κB	High NF-κB	*p-value*
**Total Patients**	78	88	
**Age, mean (SD)**	56.1 (8.1)	57.8 (8.2)	*0.176*
**Female Sex, n (%)**	6 (7.7)	5 (5.7)	*0.836*
**Smoking History, n (%)**			
< 10 pack-years	33 (42.3)	57 (64.8)	***0.006***
**TNM Stage (AJCC 7th ed.), n (%)**			
T Low-risk (T1-3)	61 (79.5)	81 (92)	***0.035***
T High-risk (T4)	16 (20.5)	7 (8)	
N Low-risk (N0-2b)	48 (62.8)	58 (65.9)	*0.801*
N High-risk (N2c-3)	29 (37.2)	30 (34.1)	
**T Stage (AJCC 8th ed.), n (%)**			
T1	24 (31.2)	22 (25)	***0.047***
T2	28 (36.4)	45 (51.1)	
T3	9 (11.7)	14 (15.9)	
T4	16 (20.8)	7 (8)	
**N Stage (AJCC 8th ed.), n (%)**			
N0	2 (2.6)	5 (5.7)	*0.729*
N1	45 (58.4)	53 (60.2)	
N2	23 (29.9)	22 (25)	
N3	7 (9.1)	8 (9.1)	
**Cohort Contribution, n (%)**			
E1308	26 (33.3)	33 (37.5)	*0.707*
TCGA	14 (17.9)	12 (13.6)	
UNC	38 (48.7)	43 (48.9)	

*One patient without initial TNM staging available

Table S5.
Combined Cohort: Univariate and Multivariate Cox Proportional Analysis for Event-Free Survival

Univariate Analysis	HR (95% CI)	*p-value (log-rank)*	# of Patients	# of Events
**NF-κB Status**			166	35
Low NF-κB	Ref	-		
High NF-κB	0.290 (0.139-0.605)	***0.001***		
**Smoking History**			166	35
< 10 pack-years	0.591 (0.302-1.154)	*0.123*		
> 10 pack-years	Ref	-		
**TNM Stage (AJCC 7th ed.)**			165	34
T Low-risk (T1-3)	Ref	-		
T High-risk (T4)	1.947 (0.881-4.303)	*0.099*		
N Low-risk (N0-2b)	Ref	-		
N High-risk (N2c-3)	1.755 (0.896-3.438)	*0.101*		
**T Stage (AJCC 8th ed.)**			165	34
T1	Ref	-		
T2	1.695 (0.610-4.707)	*0.311*		
T3	2.932 (0.930-9.243)	*0.066*		
T4	3.235 (1.058-9.891)	***0.039***		
**N Stage (AJCC 8th ed.)**			158	34
N1	Ref	-		
N2	1.835 (0.904-3.724)	*0.093*		
N3	1.001 (0.293-3.424)	*0.998*		
**Multivariate Analysis**				
Model 1			166	35
High NF-κB	0.307 (0.146-0.647)	***0.002***		
<10 pack-years	0.718 (0.364-1.414)	*0.338*		
Model 2			165	34
High NF-κB	0.328 (0.154-0.699)	***0.004***		
<10 pack-years	0.759 (0.38-1.519)	*0.437*		
High-risk T stage (AJCC 7th ed.)	1.46 (0.649-3.289)	*0.361*		
Model 3			165	34
High NF-κB	0.333 (0.155-0.712)	***0.005***		
<10 pack-years	0.756 (0.378-1.512)	*0.429*		
High-risk T stage (AJCC 7th ed.)	1.355 (0.601-3.055)	*0.463*		
High-risk N stage (AJCC 7th ed.)	1.628 (0.826-3.209)	*0.160*		

*One patient without initial TNM staging available. Adverse oncologic events were defined as recurrence or progression event, based on data availability.

SI References

1. R. Patro, G. Duggal, M. I. Love, R. A. Irizarry, C. Kingsford, Salmon provides fast and bias-aware quantification of transcript expression. *Nat Methods* **14**, 417–419 (2017).

2. M. Deng, J. Brägelmann, I. Kryukov, N. Saraiva-Agostinho, S. Perner, FirebrowseR: an R client to the Broad Institute's Firehose Pipeline. *Database (Oxford)* **2017** (2017).

3. X. Liu, *et al.*, A prognostic gene expression signature for oropharyngeal squamous cell carcinoma. *EBioMedicine* **61**, 102805 (2020).

4. M. D. Robinson, D. J. McCarthy, G. K. Smyth, edgeR: a Bioconductor package for differential expression analysis of digital gene expression data. *Bioinformatics* **26**, 139–140 (2010).

5. P. Langfelder, S. Horvath, WGCNA: an R package for weighted correlation network analysis. *BMC Bioinformatics* **9**, 559 (2008).

6. Z. Xie, *et al.*, Gene Set Knowledge Discovery with Enrichr. *Curr Protoc* **1**, e90 (2021).

7. A. Liberzon, *et al.*, The Molecular Signatures Database (MSigDB) hallmark gene set collection. *Cell Syst* **1**, 417–425 (2015).

8. B. A. Luca, *et al.*, Atlas of clinically distinct cell states and ecosystems across human solid tumors. *Cell* **184**, 5482-5496.e28 (2021).

9. A. Mayakonda, D. Lin, Y. Assenov, C. Plass, P. H. Koeffler, Maftools: efficient and comprehensive analysis of somatic variants in cancer. *Genome Research* (2018) https:/doi.org/http://dx.doi.org/10.1101/gr.239244.118.

10. C. H. Mermel, *et al.*, GISTIC2.0 facilitates sensitive and confident localization of the targets of focal somatic copy-number alteration in human cancers. *Genome Biol* **12**, R41 (2011).

11. D. C. Koboldt, *et al.*, VarScan 2: somatic mutation and copy number alteration discovery in cancer by exome sequencing. *Genome Res* **22**, 568–576 (2012).

12. L. B. Alexandrov, S. Nik-Zainal, D. C. Wedge, P. J. Campbell, M. R. Stratton, Deciphering signatures of mutational processes operative in human cancer. *Cell Rep* **3**, 246–259 (2013).

13. R. Rosenthal, *deconstructSigs: Identifies Signatures Present in a Tumor Sample* (2016).

14. E. Talevich, A. H. Shain, T. Botton, B. C. Bastian, CNVkit: Genome-Wide Copy Number Detection and Visualization from Targeted DNA Sequencing. *PLOS Computational Biology* **12**, e1004873 (2016).

15. N.-P. D. Nguyen, V. Deshpande, J. Luebeck, P. S. Mischel, V. Bafna, ViFi: accurate detection of viral integration and mRNA fusion reveals indiscriminate and unregulated transcription in proximal genomic regions in cervical cancer. *Nucleic Acids Res.* **46**, 3309–3325 (2018).

16. T. C. Silva, *et al.*, TCGA Workflow: Analyze cancer genomics and epigenomics data using Bioconductor packages. *F1000Res* **5**, 1542 (2016).

17. W. Zhou, P. W. Laird, H. Shen, Comprehensive characterization, annotation and innovative use of Infinium DNA methylation BeadChip probes. *Nucleic Acids Res* **45**, e22 (2017).

18. X. Ren, P. F. Kuan, methylGSA: a Bioconductor package and Shiny app for DNA methylation data length bias adjustment in gene set testing. *Bioinformatics* **35**, 1958–1959 (2019).

19. M. Hajek, *et al.*, TRAF3/CYLD mutations identify a distinct subset of human papillomavirus-associated head and neck squamous cell carcinoma. *Cancer* **123**, 1778–1790 (2017).
