# Supplementary material for: Noncanonical HPV carcinogenesis drives radiosensitization of head and neck tumors
Source: Proc Natl Acad Sci U S A. 2023 Jul 31;120(32):e2216532120. doi: 10.1073/pnas.2216532120 (PMC10410762; doi:10.1073/pnas.2216532120)
Supplement: Supplementary file 2 — Dataset S01 (PDF) [file pnas.2216532120.sd01.pdf]

SI Dataset: Module definitions

Gene (Hugo) Symbol	Module Size (n genes)	Module Name
A1BG	205	chocolate4
A2ML1	27	red
AAGAB	205	chocolate4
AARSD1	306	lemonchiffon4
ABCA1	205	chocolate4
ABCA3	203	skyblue1
ABCA5	158	goldenrod2
ABCB1	464	wheat2
ABCB10	205	chocolate4
ABCB6	278	darkgoldenrod4
ABCC5	38	springgreen4
ABCE1	181	seashell1
ABCF3	17	aquamarine2
ABHD14B	5	lightblue2
ABHD2	205	chocolate4
ABI1	205	chocolate4
ABI3	464	wheat2
ABTB1	278	darkgoldenrod4
ACAA1	278	darkgoldenrod4
ACAD9	17	palevioletred1
ACADVL	158	goldenrod2
ACAP1	464	wheat2
ACAP2	38	springgreen4
ACBD3	205	chocolate4
ACBD5	306	lemonchiffon4
ACBD6	181	seashell1
ACER2	158	goldenrod2
ACOT9	306	lemonchiffon4
ACSL5	464	wheat2
ACTA1	81	darkseagreen4
ACTA2	243	darkolivegreen2
ACTC1	81	darkseagreen4
ACTL6A	38	springgreen4
ACTN1	243	darkolivegreen2
ACTN2	81	darkseagreen4
ACTR5	158	goldenrod2
ACVR1B	278	darkgoldenrod4
ACVR1C	158	goldenrod2
ADAM12	243	darkolivegreen2
ADAM22	203	skyblue1
ADAMDEC1	464	wheat2

ADAMTS12	243	darkolivegreen2
ADAMTS14	243	darkolivegreen2
ADAMTS16	243	darkolivegreen2
ADAMTS2	243	darkolivegreen2
ADAMTS5	243	darkolivegreen2
ADAMTS7	243	darkolivegreen2
ADAMTS9	243	darkolivegreen2
ADAMTSL1	243	darkolivegreen2
ADARB2	203	skyblue1
ADAT3	38	mistyrose
ADCY7	278	darkgoldenrod4
ADCY9	165	cornflowerblue
ADK	306	lemonchiffon4
ADNP2	278	darkgoldenrod4
ADPRH	464	wheat2
ADRA2A	464	wheat2
ADSS	306	lemonchiffon4
AEBP1	243	darkolivegreen2
AES	38	mistyrose
AFF2	165	cornflowerblue
AFF3	464	wheat2
AFF4	181	seashell1
AGAP2	464	wheat2
AGGF1	158	goldenrod2
AGK	158	goldenrod2
AGL	181	seashell1
AGPAT3	203	skyblue1
AGPS	181	seashell1
AHI1	158	goldenrod2
AIDA	181	seashell1
AIMP1	205	chocolate4
AK2	278	darkgoldenrod4
AKAP1	278	darkgoldenrod4
AKAP2	278	darkgoldenrod4
AKAP6	81	darkseagreen4
AKNA	464	wheat2
ALDH18A1	306	lemonchiffon4
ALDH2	203	skyblue1
ALDH3B2	65	thistle4
ALG10	158	goldenrod2
ALG14	306	lemonchiffon4
ALG5	306	lemonchiffon4
ALKBH3	205	chocolate4
ALKBH4	306	lemonchiffon4
ALKBH8	278	darkgoldenrod4
ALOX12B	64	lightsalmon2

ALOX15	165	cornflowerblue
ALOX5	71	mediumpurple1
ALPK3	81	darkseagreen4
ALX1	165	cornflowerblue
AMIGO1	278	darkgoldenrod4
AMN	158	goldenrod2
AMPD2	243	darkolivegreen2
AMPD3	203	skyblue1
ANAPC16	306	lemonchiffon4
ANG	205	chocolate4
ANGPTL2	243	darkolivegreen2
ANK3	165	cornflowerblue
ANKH	203	skyblue1
ANKHD1	181	seashell1
ANKIB1	181	seashell1
ANKRA2	306	lemonchiffon4
ANKRD1	81	darkseagreen4
ANKRD13A	278	darkgoldenrod4
ANKRD29	203	skyblue1
ANKRD44	278	darkgoldenrod4
ANKRD50	278	darkgoldenrod4
ANKS3	205	chocolate4
ANO4	203	skyblue1
ANO5	81	darkseagreen4
ANP32B	205	chocolate4
ANPEP	243	darkolivegreen2
ANXA1	27	red
ANXA6	464	wheat2
ANXA7	181	seashell1
AOAH	71	mediumpurple1
AP2M1	17	aquamarine2
AP3B1	205	chocolate4
AP4B1	278	darkgoldenrod4
AP4S1	306	lemonchiffon4
APBA1	158	goldenrod2
APBB1IP	464	wheat2
APBB2	243	darkolivegreen2
APEH	5	lightblue2
APEX1	82	hotpink2
APH1B	464	wheat2
APLN	464	wheat2
APLP1	205	chocolate4
APOBEC3C	278	darkgoldenrod4
APOBEC3G	464	wheat2
APOOL	165	cornflowerblue
AQP3	65	thistle4

ARAP1	71	mediumpurple1
ARCN1	181	seashell1
ARFGAP2	158	goldenrod2
ARFGAP3	181	seashell1
ARHGAP15	464	wheat2
ARHGAP17	464	wheat2
ARHGAP21	278	darkgoldenrod4
ARHGAP23	65	thistle4
ARHGAP24	165	cornflowerblue
ARHGAP25	464	wheat2
ARHGAP26	203	skyblue1
ARHGAP27	65	thistle4
ARHGAP30	464	wheat2
ARHGAP9	464	wheat2
ARHGDIB	464	wheat2
ARHGEF1	464	wheat2
ARHGEF10L	65	thistle4
ARHGEF17	243	darkolivegreen2
ARHGEF37	65	thistle4
ARHGEF6	464	wheat2
ARIH1	158	goldenrod2
ARL16	306	lemonchiffon4
ARL4C	165	cornflowerblue
ARL6IP1	306	lemonchiffon4
ARMC8	33	lightpink1
ARMCX1	243	darkolivegreen2
ARNT2	203	skyblue1
ARPC3	181	seashell1
ARPC5	306	lemonchiffon4
ARPP19	181	seashell1
ARPP21	81	darkseagreen4
ARRDC3	181	seashell1
ARSK	306	lemonchiffon4
ARV1	205	chocolate4
ARVCF	278	darkgoldenrod4
AS3MT	306	lemonchiffon4
ASAP2	278	darkgoldenrod4
ASB5	81	darkseagreen4
ASB7	205	chocolate4
ASCL4	203	skyblue1
ASH1L	158	goldenrod2
ASPH	278	darkgoldenrod4
ASPN	243	darkolivegreen2
ATAD3C	306	lemonchiffon4
ATF5	203	skyblue1
ATF7IP	278	darkgoldenrod4

ATG3	17	palevioletred1
ATL2	203	skyblue1
ATL3	181	seashell1
ATM	278	darkgoldenrod4
ATP10B	27	red
ATP10D	205	chocolate4
ATP11A	165	cornflowerblue
ATP11B	38	springgreen4
ATP12A	64	lightsalmon2
ATP13A3	38	springgreen4
ATP13A4	65	thistle4
ATP2A3	464	wheat2
ATP2C1	33	lightpink1
ATP6V0A2	306	lemonchiffon4
ATP6V0A4	64	lightsalmon2
ATP6V0E1	181	seashell1
ATP6V1A	33	lightpink1
ATP6V1E1	181	seashell1
ATP6V1H	205	chocolate4
ATP8A1	464	wheat2
ATP8B2	464	wheat2
ATR	33	lightpink1
AVL9	158	goldenrod2
AXIN2	243	darkolivegreen2
AXL	243	darkolivegreen2
B3GALNT2	278	darkgoldenrod4
B3GALT5	165	cornflowerblue
B3GNT2	306	lemonchiffon4
B3GNT5	38	springgreen4
B3GNT6	306	lemonchiffon4
B4GALT4	17	aquamarine2
B4GALT6	203	skyblue1
B9D2	158	goldenrod2
BACE1	243	darkolivegreen2
BACH2	278	darkgoldenrod4
BAIAP2L1	464	wheat2
BANK1	464	wheat2
BATF	464	wheat2
BAZ1A	205	chocolate4
BCAR3	165	cornflowerblue
BCAS2	181	seashell1
BCAT1	243	darkolivegreen2
BCL11B	464	wheat2
BCL2	203	skyblue1
BCL6B	243	darkolivegreen2
BCL9	278	darkgoldenrod4

BCS1L	165	cornflowerblue
BGN	243	darkolivegreen2
BHLHB9	205	chocolate4
BICC1	243	darkolivegreen2
BIN2	464	wheat2
BIRC3	203	skyblue1
BLCAP	158	goldenrod2
BLK	464	wheat2
BLOC1S2	306	lemonchiffon4
BMPR1B	464	wheat2
BNC2	243	darkolivegreen2
BOC	243	darkolivegreen2
BOLA3	205	chocolate4
BRF2	165	cornflowerblue
BRSK1	205	chocolate4
BRWD1	158	goldenrod2
BRWD3	165	cornflowerblue
BTBD1	306	lemonchiffon4
BTBD2	38	mistyrose
BTF3	82	hotpink2
BTG2	278	darkgoldenrod4
BTK	464	wheat2
BTLA	464	wheat2
BTNL9	203	skyblue1
BUD13	205	chocolate4
C10orf71	81	darkseagreen4
C11orf1	205	chocolate4
C11orf45	158	goldenrod2
C11orf80	165	cornflowerblue
C12orf4	165	cornflowerblue
C12orf43	306	lemonchiffon4
C12orf75	205	chocolate4
C14orf119	205	chocolate4
C15orf39	65	thistle4
C16orf45	243	darkolivegreen2
C16orf54	464	wheat2
C16orf72	158	goldenrod2
C17orf75	306	lemonchiffon4
C18orf25	165	cornflowerblue
C1orf116	65	thistle4
C1orf162	71	mediumpurple1
C1orf216	306	lemonchiffon4
C1QTNF1	243	darkolivegreen2
C1QTNF2	306	lemonchiffon4
C1QTNF4	205	chocolate4
C1R	243	darkolivegreen2

C1S	243	darkolivegreen2
C20orf96	158	goldenrod2
C2CD2	203	skyblue1
C2CD3	205	chocolate4
C3orf38	181	seashell1
C3orf52	464	wheat2
C3orf58	33	lightpink1
C6orf132	65	thistle4
C6orf203	205	chocolate4
C6orf223	464	wheat2
C7	464	wheat2
C8G	203	skyblue1
C9orf129	165	cornflowerblue
C9orf3	205	chocolate4
C9orf64	306	lemonchiffon4
C9orf78	306	lemonchiffon4
CA5B	306	lemonchiffon4
CAB39	306	lemonchiffon4
CACHD1	165	cornflowerblue
CACNA1C	243	darkolivegreen2
CACNA1S	81	darkseagreen4
CACNB4	205	chocolate4
CACYBP	181	seashell1
CADPS2	203	skyblue1
CALCRL	243	darkolivegreen2
CALD1	243	darkolivegreen2
CALM1	203	skyblue1
CALML3	65	thistle4
CAMK1D	306	lemonchiffon4
CAMK2G	306	lemonchiffon4
CAMK4	278	darkgoldenrod4
CANX	306	lemonchiffon4
CAP1	181	seashell1
CAPN14	27	red
CAPS	306	lemonchiffon4
CAPZA1	181	seashell1
CARD11	464	wheat2
CARD8	464	wheat2
CASKIN2	278	darkgoldenrod4
CASP2	158	goldenrod2
CASQ2	81	darkseagreen4
CBL	278	darkgoldenrod4
CBLN2	203	skyblue1
CBLN3	203	skyblue1
CBR1	158	goldenrod2
CBWD1	158	goldenrod2

CBX1	464	wheat2
CBX6	165	cornflowerblue
CBY1	278	darkgoldenrod4
CCDC120	65	thistle4
CCDC126	306	lemonchiffon4
CCDC127	205	chocolate4
CCDC152	71	mediumpurple1
CCDC155	205	chocolate4
CCDC24	181	seashell1
CCDC3	243	darkolivegreen2
CCDC47	205	chocolate4
CCDC50	38	springgreen4
CCDC58	17	palevioletred1
CCDC69	464	wheat2
CCDC8	203	skyblue1
CCDC82	158	goldenrod2
CCL19	464	wheat2
CCL20	203	skyblue1
CCL21	464	wheat2
CCL4L2	464	wheat2
CCND1	203	skyblue1
CCND2	464	wheat2
CCND3	278	darkgoldenrod4
CCNJ	158	goldenrod2
CCR2	464	wheat2
CCR4	278	darkgoldenrod4
CCR5	464	wheat2
CCR7	464	wheat2
CD109	278	darkgoldenrod4
CD163L1	71	mediumpurple1
CD180	464	wheat2
CD19	464	wheat2
CD1C	464	wheat2
CD2	464	wheat2
CD200R1	464	wheat2
CD22	464	wheat2
CD24	27	red
CD244	464	wheat2
CD247	464	wheat2
CD248	243	darkolivegreen2
CD27	464	wheat2
CD274	464	wheat2
CD28	464	wheat2
CD37	464	wheat2
CD3D	464	wheat2
CD3E	464	wheat2

CD3G	464	wheat2
CD4	71	mediumpurple1
CD44	306	lemonchiffon4
CD48	464	wheat2
CD5	464	wheat2
CD52	464	wheat2
CD53	464	wheat2
CD63	306	lemonchiffon4
CD69	464	wheat2
CD7	464	wheat2
CD72	464	wheat2
CD74	464	wheat2
CD79A	464	wheat2
CD79B	464	wheat2
CD80	464	wheat2
CD83	464	wheat2
CD84	464	wheat2
CD86	71	mediumpurple1
CD8A	464	wheat2
CD8B	464	wheat2
CD93	243	darkolivegreen2
CD96	464	wheat2
CD99	306	lemonchiffon4
CDA	64	lightsalmon2
CDC123	181	seashell1
CDC14A	278	darkgoldenrod4
CDC26	205	chocolate4
CDC37L1	205	chocolate4
CDC42EP4	203	skyblue1
CDC42SE1	165	cornflowerblue
CDC42SE2	464	wheat2
CDC45	278	darkgoldenrod4
CDH11	243	darkolivegreen2
CDH5	243	darkolivegreen2
CDK12	306	lemonchiffon4
CDK18	203	skyblue1
CDKAL1	306	lemonchiffon4
CDKN2AIPNL	306	lemonchiffon4
CDON	203	skyblue1
CDRT4	203	skyblue1
CDV3	33	lightpink1
CDYL	158	goldenrod2
CDYL2	278	darkgoldenrod4
CEACAM1	27	red
CEACAM5	27	red
CEACAM6	27	red

CEACAM7	9	slateblue4
CEBPB	278	darkgoldenrod4
CELF1	165	cornflowerblue
CELF2	464	wheat2
CELSR1	278	darkgoldenrod4
CEMP1	181	seashell1
CEP120	278	darkgoldenrod4
CEP135	203	skyblue1
CEP70	17	palevioletred1
CFTR	165	cornflowerblue
CGN	65	thistle4
CHCHD4	205	chocolate4
CHCHD5	306	lemonchiffon4
CHCHD7	158	goldenrod2
CHGB	81	darkseagreen4
CHI3L2	464	wheat2
CHIC2	205	chocolate4
CHIT1	71	mediumpurple1
CHML	278	darkgoldenrod4
CHMP1B	158	goldenrod2
CHMP7	464	wheat2
CHPF	243	darkolivegreen2
CHRD	243	darkolivegreen2
CHRNA1	81	darkseagreen4
CHST11	71	mediumpurple1
CHST2	464	wheat2
CHST3	278	darkgoldenrod4
CILP	81	darkseagreen4
CILP2	243	darkolivegreen2
CINP	205	chocolate4
CISH	464	wheat2
CKM	81	darkseagreen4
CKS2	306	lemonchiffon4
CLCN3	205	chocolate4
CLDN3	203	skyblue1
CLDND1	33	lightpink1
CLEC10A	464	wheat2
CLEC12A	71	mediumpurple1
CLEC2D	464	wheat2
CLIC3	64	lightsalmon2
CLIC6	165	cornflowerblue
CLINT1	205	chocolate4
CLIP2	203	skyblue1
CLIP3	203	skyblue1
CLK4	203	skyblue1
CLMN	165	cornflowerblue

CLPB	205	chocolate4
CLSTN2	243	darkolivegreen2
CLTC	181	seashell1
CLUAP1	306	lemonchiffon4
CMTM6	181	seashell1
CMTM7	464	wheat2
CMYA5	81	darkseagreen4
CNBP	17	palevioletred1
CNFN	64	lightsalmon2
CNNM4	158	goldenrod2
CNOT4	158	goldenrod2
CNPY2	205	chocolate4
CNPY4	306	lemonchiffon4
CNR1	464	wheat2
CNTN1	165	cornflowerblue
CNTNAP2	203	skyblue1
CNTROB	278	darkgoldenrod4
COG1	205	chocolate4
COL12A1	243	darkolivegreen2
COL15A1	243	darkolivegreen2
COL16A1	203	skyblue1
COL18A1	243	darkolivegreen2
COL19A1	203	skyblue1
COL1A1	243	darkolivegreen2
COL1A2	243	darkolivegreen2
COL22A1	203	skyblue1
COL23A1	203	skyblue1
COL3A1	243	darkolivegreen2
COL4A1	243	darkolivegreen2
COL4A2	243	darkolivegreen2
COL4A4	203	skyblue1
COL4A5	278	darkgoldenrod4
COL5A1	243	darkolivegreen2
COL5A2	243	darkolivegreen2
COL5A3	243	darkolivegreen2
COL6A1	243	darkolivegreen2
COL6A2	243	darkolivegreen2
COL6A3	243	darkolivegreen2
COL6A6	278	darkgoldenrod4
COL7A1	278	darkgoldenrod4
COL8A1	243	darkolivegreen2
COL8A2	243	darkolivegreen2
COLEC11	205	chocolate4
COMMD8	181	seashell1
COMMD9	306	lemonchiffon4
COMT	278	darkgoldenrod4

COPA	306	lemonchiffon4
COPB1	181	seashell1
COPS7B	205	chocolate4
COQ6	205	chocolate4
CORO1A	464	wheat2
COTL1	464	wheat2
COX19	306	lemonchiffon4
COX7A2	181	seashell1
COX7C	306	lemonchiffon4
CPNE5	464	wheat2
CPS1	205	chocolate4
CPSF3	181	seashell1
CPXM1	243	darkolivegreen2
CPXM2	243	darkolivegreen2
CR1	464	wheat2
CR2	464	wheat2
CRB2	203	skyblue1
CRCT1	64	lightsalmon2
CREB3	306	lemonchiffon4
CREB3L1	203	skyblue1
CREB3L2	278	darkgoldenrod4
CRISP3	64	lightsalmon2
CRISPLD2	243	darkolivegreen2
CRNKL1	181	seashell1
CRNN	64	lightsalmon2
CRTC1	38	mistyrose
CRTC3	278	darkgoldenrod4
CRYL1	278	darkgoldenrod4
CRYM	9	slateblue4
CS	306	lemonchiffon4
CSDE1	181	seashell1
CSK	464	wheat2
CSMD2	243	darkolivegreen2
CSNK1E	464	wheat2
CSNK1G2	38	mistyrose
CSRP3	81	darkseagreen4
CST1	243	darkolivegreen2
CST7	464	wheat2
CSTB	27	red
CT45A1	205	chocolate4
CTBP2	464	wheat2
CTDP1	38	mistyrose
CTGF	243	darkolivegreen2
CTHRC1	243	darkolivegreen2
CTLA4	464	wheat2
CTNNBL1	205	chocolate4

CTR9	306	lemonchiffon4
CTSC	71	mediumpurple1
CTSE	203	skyblue1
CTSH	71	mediumpurple1
CTSK	243	darkolivegreen2
CTSW	464	wheat2
CTTN	464	wheat2
CUEDC1	464	wheat2
CUL1	306	lemonchiffon4
CUL2	306	lemonchiffon4
CUL4A	165	cornflowerblue
CUL5	278	darkgoldenrod4
CWC22	205	chocolate4
CWF19L1	306	lemonchiffon4
CWF19L2	278	darkgoldenrod4
CWH43	64	lightsalmon2
CXCL13	464	wheat2
CXCL17	65	thistle4
CXCL9	464	wheat2
CXCR3	464	wheat2
CXCR4	464	wheat2
CXCR5	464	wheat2
CXorf38	306	lemonchiffon4
CYB5D2	306	lemonchiffon4
CYBA	278	darkgoldenrod4
CYBB	71	mediumpurple1
CYFIP2	464	wheat2
CYHR1	38	mistyrose
CYLD	278	darkgoldenrod4
CYP11A1	205	chocolate4
CYP20A1	306	lemonchiffon4
CYP26B1	243	darkolivegreen2
CYP2C18	65	thistle4
CYP3A5	9	slateblue4
CYP4F22	64	lightsalmon2
CYP7B1	165	cornflowerblue
CYR61	243	darkolivegreen2
CYTH3	243	darkolivegreen2
CYTH4	464	wheat2
CYTIP	464	wheat2
DACT1	243	darkolivegreen2
DACT2	203	skyblue1
DAD1	306	lemonchiffon4
DAPK3	38	mistyrose
DBN1	464	wheat2
DBNDD2	205	chocolate4

DCAF15	38	mistyrose
DCAF7	165	cornflowerblue
DCBLD2	278	darkgoldenrod4
DCHS1	243	darkolivegreen2
DCN	243	darkolivegreen2
DCUN1D1	38	springgreen4
DCUN1D2	306	lemonchiffon4
DCUN1D5	306	lemonchiffon4
DDB1	306	lemonchiffon4
DDHD1	278	darkgoldenrod4
DDIT4L	81	darkseagreen4
DDN	81	darkseagreen4
DDR2	243	darkolivegreen2
DDTL	181	seashell1
DDX46	205	chocolate4
DDX51	306	lemonchiffon4
DDX6	278	darkgoldenrod4
DEDD2	278	darkgoldenrod4
DEF6	464	wheat2
DEFB1	158	goldenrod2
DENND1C	464	wheat2
DENND2D	464	wheat2
DENND4B	464	wheat2
DERL3	464	wheat2
DES	81	darkseagreen4
DGAT1	38	mistyrose
DHRS12	278	darkgoldenrod4
DHRS7B	205	chocolate4
DHTKD1	306	lemonchiffon4
DHX15	165	cornflowerblue
DHX29	306	lemonchiffon4
DHX36	33	lightpink1
DHX57	158	goldenrod2
DHX8	205	chocolate4
DHX9	181	seashell1
DIAPH3	278	darkgoldenrod4
DIP2A	306	lemonchiffon4
DIRC2	17	palevioletred1
DIS3L	306	lemonchiffon4
DIXDC1	243	darkolivegreen2
DKK3	243	darkolivegreen2
DLC1	243	darkolivegreen2
DLG1	38	springgreen4
DLG5	278	darkgoldenrod4
DMBX1	306	lemonchiffon4
DMD	203	skyblue1

DMKN	64	lightsalmon2
DMXL1	278	darkgoldenrod4
DNAH1	464	wheat2
DNAH11	203	skyblue1
DNAH14	278	darkgoldenrod4
DNAJB11	17	aquamarine2
DNAJB4	205	chocolate4
DNAJC13	33	lightpink1
DNAJC30	306	lemonchiffon4
DNAJC7	205	chocolate4
DNAL1	306	lemonchiffon4
DNASE1L3	464	wheat2
DOCK10	464	wheat2
DOCK11	464	wheat2
DOCK2	464	wheat2
DOCK5	278	darkgoldenrod4
DOCK8	464	wheat2
DOK1	71	mediumpurple1
DOK2	464	wheat2
DOK3	464	wheat2
DOT1L	38	mistyrose
DPF2	205	chocolate4
DPM1	181	seashell1
DPYSL3	243	darkolivegreen2
DRAM1	203	skyblue1
DRG1	205	chocolate4
DSCAML1	203	skyblue1
DSG1	64	lightsalmon2
DSG2	278	darkgoldenrod4
DSN1	278	darkgoldenrod4
DST	278	darkgoldenrod4
DSTYK	306	lemonchiffon4
DTNA	81	darkseagreen4
DTWD2	306	lemonchiffon4
DTX1	464	wheat2
DTX3L	33	lightpink1
DUOX1	65	thistle4
DUOXA1	65	thistle4
DUOXA2	65	thistle4
DUSP12	306	lemonchiffon4
DUSP13	81	darkseagreen4
DUSP18	306	lemonchiffon4
DUSP27	81	darkseagreen4
DUSP4	464	wheat2
DUSP9	464	wheat2
DVL3	38	springgreen4

DYNC2LI1	205	chocolate4
DYNLL1	181	seashell1
DYNLL2	165	cornflowerblue
DYRK2	278	darkgoldenrod4
E2F6	278	darkgoldenrod4
EAF1	158	goldenrod2
EAF2	278	darkgoldenrod4
EBI3	464	wheat2
ECE1	203	skyblue1
ECHS1	158	goldenrod2
ECM1	64	lightsalmon2
ECT2	38	springgreen4
EDEM1	278	darkgoldenrod4
EDEM3	181	seashell1
EED	278	darkgoldenrod4
EEF1A1	82	hotpink2
EEF1A2	81	darkseagreen4
EEF1B2	82	hotpink2
EEF1E1	306	lemonchiffon4
EEF1G	82	hotpink2
EEF2	82	hotpink2
EFCAB2	306	lemonchiffon4
EFR3A	181	seashell1
EGFR	278	darkgoldenrod4
EGLN2	38	mistyrose
EHBP1	278	darkgoldenrod4
EHD1	464	wheat2
EHHADH	38	springgreen4
EID2	165	cornflowerblue
EIF1	306	lemonchiffon4
EIF2A	33	lightpink1
EIF2B1	205	chocolate4
EIF2B5	17	aquamarine2
EIF3D	82	hotpink2
EIF3E	82	hotpink2
EIF3F	82	hotpink2
EIF3H	82	hotpink2
EIF3L	82	hotpink2
EIF4A2	38	springgreen4
EIF4E3	464	wheat2
EIF4G1	38	springgreen4
EIF4G2	181	seashell1
EIF5B	306	lemonchiffon4
ELF1	181	seashell1
ELF3	65	thistle4
ELF4	158	goldenrod2

ELFN1	243	darkolivegreen2
ELMO1	464	wheat2
EMB	464	wheat2
EMILIN1	243	darkolivegreen2
EML1	243	darkolivegreen2
EML4	278	darkgoldenrod4
EMP1	9	slateblue4
ENAH	278	darkgoldenrod4
ENC1	243	darkolivegreen2
ENDOU	64	lightsalmon2
ENPP2	464	wheat2
ENPP6	203	skyblue1
ENSA	306	lemonchiffon4
ENTPD2	278	darkgoldenrod4
ENTPD7	158	goldenrod2
EOMES	464	wheat2
EP300	181	seashell1
EP400	181	seashell1
EPAS1	158	goldenrod2
EPC1	278	darkgoldenrod4
EPHA1	65	thistle4
EPHA2	65	thistle4
EPHA4	165	cornflowerblue
EPHA6	158	goldenrod2
EPHB1	203	skyblue1
EPN3	65	thistle4
EPOR	165	cornflowerblue
EPS15	278	darkgoldenrod4
EPS8L1	9	slateblue4
EPS8L2	65	thistle4
EPYC	243	darkolivegreen2
ERAL1	158	goldenrod2
ERCC4	306	lemonchiffon4
ERCC8	205	chocolate4
ERGIC1	165	cornflowerblue
ERI3	278	darkgoldenrod4
ERLIN1	205	chocolate4
ERMAP	205	chocolate4
ERN1	278	darkgoldenrod4
ERN2	205	chocolate4
ESM1	278	darkgoldenrod4
ESRP2	65	thistle4
ETF1	181	seashell1
ETS1	464	wheat2
ETV6	203	skyblue1
EVI2A	464	wheat2

EVI2B	464	wheat2
EVL	464	wheat2
EVPL	65	thistle4
EXOC2	306	lemonchiffon4
EXOC6B	278	darkgoldenrod4
EXOC8	165	cornflowerblue
EXOSC1	205	chocolate4
EXOSC2	306	lemonchiffon4
EXOSC8	165	cornflowerblue
EXOSC9	165	cornflowerblue
F2RL1	203	skyblue1
F8A1	165	cornflowerblue
FAM107B	464	wheat2
FAM120C	158	goldenrod2
FAM129A	165	cornflowerblue
FAM129B	65	thistle4
FAM131A	17	aquamarine2
FAM13B	278	darkgoldenrod4
FAM161B	306	lemonchiffon4
FAM162A	17	palevioletred1
FAM167A	203	skyblue1
FAM169A	165	cornflowerblue
FAM171A1	203	skyblue1
FAM177A1	306	lemonchiffon4
FAM189A2	203	skyblue1
FAM198B	243	darkolivegreen2
FAM25A	64	lightsalmon2
FAM3B	243	darkolivegreen2
FAM3D	64	lightsalmon2
FAM49A	464	wheat2
FAM53B	203	skyblue1
FAM76B	278	darkgoldenrod4
FAM78A	464	wheat2
FAM98A	205	chocolate4
FAP	243	darkolivegreen2
FAS	203	skyblue1
FASLG	464	wheat2
FASTKD5	205	chocolate4
FAT1	278	darkgoldenrod4
FBLN1	278	darkgoldenrod4
FBLN2	243	darkolivegreen2
FBN1	243	darkolivegreen2
FBXL17	165	cornflowerblue
FBXL20	306	lemonchiffon4
FBXO21	181	seashell1
FBXO3	158	goldenrod2

FBXO36	205	chocolate4
FBXO44	306	lemonchiffon4
FBXO45	38	springgreen4
FBXW11	306	lemonchiffon4
FBXW2	165	cornflowerblue
FCAMR	464	wheat2
FCER2	464	wheat2
FCGR2B	71	mediumpurple1
FCHO1	464	wheat2
FCHO2	306	lemonchiffon4
FCHSD2	278	darkgoldenrod4
FCRL1	464	wheat2
FCRL3	464	wheat2
FCRL4	464	wheat2
FCRL5	464	wheat2
FCRLA	464	wheat2
FEM1B	181	seashell1
FEM1C	181	seashell1
FERMT1	278	darkgoldenrod4
FERMT2	243	darkolivegreen2
FERMT3	464	wheat2
FGD2	464	wheat2
FGD3	464	wheat2
FGD5	243	darkolivegreen2
FGF2	203	skyblue1
FGFR1	243	darkolivegreen2
FGFR2	278	darkgoldenrod4
FGR	71	mediumpurple1
FH	181	seashell1
FHOD3	203	skyblue1
FILIP1L	243	darkolivegreen2
FIS1	158	goldenrod2
FKBP10	243	darkolivegreen2
FKBP1A	306	lemonchiffon4
FLCN	306	lemonchiffon4
FLI1	464	wheat2
FLNC	81	darkseagreen4
FLRT2	243	darkolivegreen2
FMNL1	464	wheat2
FN1	243	darkolivegreen2
FNBP1	464	wheat2
FNBP1L	278	darkgoldenrod4
FNDC1	243	darkolivegreen2
FNDC3A	278	darkgoldenrod4
FNDC4	243	darkolivegreen2
FOXG1	165	cornflowerblue

FOXJ3	278	darkgoldenrod4
FOXN1	203	skyblue1
FOXO1	278	darkgoldenrod4
FOXO4	165	cornflowerblue
FOXP3	464	wheat2
FOXQ1	203	skyblue1
FRMD4A	203	skyblue1
FRZB	464	wheat2
FSTL1	243	darkolivegreen2
FUBP3	205	chocolate4
FUCA1	464	wheat2
FUT3	65	thistle4
FUT6	65	thistle4
FUT7	464	wheat2
FUT8	278	darkgoldenrod4
FXR1	38	springgreen4
FXYD2	203	skyblue1
FXYD6	203	skyblue1
FYN	464	wheat2
FYTTD1	38	springgreen4
FZD7	203	skyblue1
FZR1	38	mistyrose
G0S2	243	darkolivegreen2
G3BP2	181	seashell1
GAB3	464	wheat2
GABPB2	306	lemonchiffon4
GAD1	203	skyblue1
GALNT1	181	seashell1
GALNT11	203	skyblue1
GANAB	158	goldenrod2
GANC	278	darkgoldenrod4
GAPT	464	wheat2
GAR1	205	chocolate4
GART	306	lemonchiffon4
GAS1	243	darkolivegreen2
GATA3	464	wheat2
GATAD1	306	lemonchiffon4
GBE1	306	lemonchiffon4
GBGT1	71	mediumpurple1
GBP5	464	wheat2
GCC1	165	cornflowerblue
GCK	165	cornflowerblue
GCNT2	165	cornflowerblue
GCNT4	278	darkgoldenrod4
GDI1	158	goldenrod2
GFI1	464	wheat2

GFM1	33	lightpink1
GFPT2	243	darkolivegreen2
GGA1	181	seashell1
GGA2	203	skyblue1
GGCX	306	lemonchiffon4
GGNBP2	165	cornflowerblue
GGT5	243	darkolivegreen2
GGTLC2	165	cornflowerblue
GHR	306	lemonchiffon4
GIMAP1	464	wheat2
GIMAP4	464	wheat2
GIMAP5	464	wheat2
GIMAP6	464	wheat2
GIMAP7	464	wheat2
GIMAP8	464	wheat2
GIPC1	65	thistle4
GIPC2	203	skyblue1
GIT2	464	wheat2
GJA1	306	lemonchiffon4
GJA3	203	skyblue1
GJB4	203	skyblue1
GLCCI1	464	wheat2
GLIPR1	71	mediumpurple1
GLIS2	243	darkolivegreen2
GLIS3	243	darkolivegreen2
GLT8D2	243	darkolivegreen2
GLYCTK	278	darkgoldenrod4
GMEB1	306	lemonchiffon4
GMFG	464	wheat2
GMIP	464	wheat2
GMPPB	5	lightblue2
GMPS	33	lightpink1
GNA13	181	seashell1
GNB1	306	lemonchiffon4
GNE	306	lemonchiffon4
GNG10	181	seashell1
GNG11	243	darkolivegreen2
GNG12	181	seashell1
GNG2	464	wheat2
GNG5	181	seashell1
GNG7	464	wheat2
GNGT2	464	wheat2
GNLY	464	wheat2
GNS	181	seashell1
GOLGA6L1	306	lemonchiffon4
GOLGA6L6	306	lemonchiffon4

GOLIM4	38	springgreen4
GOLPH3	181	seashell1
GOLPH3L	205	chocolate4
GORASP2	181	seashell1
GOSR1	306	lemonchiffon4
GPC6	243	darkolivegreen2
GPKOW	158	goldenrod2
GPN1	181	seashell1
GPR132	464	wheat2
GPR143	205	chocolate4
GPR171	464	wheat2
GPR18	464	wheat2
GPR183	464	wheat2
GPR55	464	wheat2
GPRIN3	278	darkgoldenrod4
GPSM2	278	darkgoldenrod4
GPSM3	464	wheat2
GPT2	65	thistle4
GPX4	158	goldenrod2
GRAP	464	wheat2
GRAP2	464	wheat2
GREB1	306	lemonchiffon4
GRHL1	65	thistle4
GRHL2	278	darkgoldenrod4
GRHL3	65	thistle4
GRIN2A	203	skyblue1
GRK6	278	darkgoldenrod4
GRPEL1	205	chocolate4
GSK3B	33	lightpink1
GSN	181	seashell1
GSPT1	181	seashell1
GSR	158	goldenrod2
GSTK1	278	darkgoldenrod4
GSTO1	306	lemonchiffon4
GSTZ1	205	chocolate4
GTF2H3	306	lemonchiffon4
GTF2H5	306	lemonchiffon4
GTF3C2	158	goldenrod2
GTPBP4	464	wheat2
GULP1	165	cornflowerblue
GUSB	165	cornflowerblue
GZMA	464	wheat2
GZMB	464	wheat2
GZMH	464	wheat2
GZMK	464	wheat2
GZMM	464	wheat2

H2AFZ	306	lemonchiffon4
H3F3A	181	seashell1
HADHA	306	lemonchiffon4
HAP1	205	chocolate4
HARBI1	181	seashell1
HARS	205	chocolate4
HARS2	205	chocolate4
HCK	71	mediumpurple1
HCLS1	464	wheat2
HDAC3	205	chocolate4
HEATR5A	306	lemonchiffon4
HECTD1	205	chocolate4
HECW2	165	cornflowerblue
HELZ	165	cornflowerblue
HEPH	243	darkolivegreen2
HEPHL1	64	lightsalmon2
HERC1	278	darkgoldenrod4
HERC2	158	goldenrod2
HERC3	278	darkgoldenrod4
HERPUD1	464	wheat2
HERPUD2	278	darkgoldenrod4
HES1	464	wheat2
HEXB	181	seashell1
HEXIM2	165	cornflowerblue
HEYL	243	darkolivegreen2
HHLA3	306	lemonchiffon4
HIBADH	306	lemonchiffon4
HIC1	243	darkolivegreen2
HIF1A	205	chocolate4
HIST3H2A	165	cornflowerblue
HIVEP2	205	chocolate4
HLA.DMA	71	mediumpurple1
HLA.DMB	71	mediumpurple1
HLA.DOA	464	wheat2
HLA.DOB	464	wheat2
HLA.DPA1	71	mediumpurple1
HLA.DPB1	71	mediumpurple1
HLA.DQA1	71	mediumpurple1
HLA.DQB1	71	mediumpurple1
HLA.DRA	71	mediumpurple1
HLA.DRB1	71	mediumpurple1
HLA.DRB5	71	mediumpurple1
HM13	306	lemonchiffon4
HMCN1	243	darkolivegreen2
HMG20B	38	mistyrose
HMGCL	205	chocolate4

HMGCR	306	lemonchiffon4
HMX2	165	cornflowerblue
HNF4G	165	cornflowerblue
HNMT	71	mediumpurple1
HNRNPC	181	seashell1
HNRNPK	181	seashell1
HNRNPR	158	goldenrod2
HOMER2	203	skyblue1
HOMER3	278	darkgoldenrod4
HOPX	64	lightsalmon2
HOXD9	165	cornflowerblue
HPCAL1	306	lemonchiffon4
HPS1	278	darkgoldenrod4
HPS3	33	lightpink1
HRC	81	darkseagreen4
HRH1	278	darkgoldenrod4
HS1BP3	165	cornflowerblue
HSCB	306	lemonchiffon4
HSD17B4	205	chocolate4
HSD17B7	205	chocolate4
HSDL2	306	lemonchiffon4
HSH2D	464	wheat2
HSP90B1	306	lemonchiffon4
HSPA13	181	seashell1
HSPA4	205	chocolate4
HSPB3	81	darkseagreen4
HSPB6	81	darkseagreen4
HSPB7	81	darkseagreen4
HSPE1	181	seashell1
HTATIP2	205	chocolate4
HTRA1	243	darkolivegreen2
HTRA3	243	darkolivegreen2
HVCN1	464	wheat2
HYLS1	306	lemonchiffon4
IAH1	205	chocolate4
IARS	306	lemonchiffon4
ICAM1	203	skyblue1
ICAM2	464	wheat2
ICAM3	464	wheat2
ICAM5	203	skyblue1
ICOS	464	wheat2
ICOSLG	203	skyblue1
IDE	306	lemonchiffon4
IDH3B	158	goldenrod2
IER5	165	cornflowerblue
IFFO1	71	mediumpurple1

IFI27L1	165	cornflowerblue
IFNAR2	464	wheat2
IFNG	464	wheat2
IFRD2	5	lightblue2
IFT52	306	lemonchiffon4
IFT81	278	darkgoldenrod4
IGDCC4	243	darkolivegreen2
IGF1R	278	darkgoldenrod4
IGF2	243	darkolivegreen2
IGFBP4	243	darkolivegreen2
IGFBP5	243	darkolivegreen2
IGFBP7	243	darkolivegreen2
IGFBPL1	278	darkgoldenrod4
IGSF9B	203	skyblue1
IKZF1	464	wheat2
IKZF3	278	darkgoldenrod4
IL11	306	lemonchiffon4
IL15	203	skyblue1
IL16	464	wheat2
IL17REL	203	skyblue1
IL18	71	mediumpurple1
IL18RAP	464	wheat2
IL1RN	64	lightsalmon2
IL21R	464	wheat2
IL27RA	203	skyblue1
IL2RA	464	wheat2
IL2RB	464	wheat2
IL2RG	464	wheat2
IL33	278	darkgoldenrod4
IL6R	278	darkgoldenrod4
ILF2	181	seashell1
ILKAP	205	chocolate4
IMMP1L	306	lemonchiffon4
IMP3	205	chocolate4
IMPDH1	158	goldenrod2
IMPDH2	82	hotpink2
ING1	203	skyblue1
INHBA	243	darkolivegreen2
INO80C	158	goldenrod2
INPP1	203	skyblue1
INPP4B	278	darkgoldenrod4
INPP5D	464	wheat2
INSM1	203	skyblue1
INTS7	306	lemonchiffon4
IP6K3	81	darkseagreen4
IPCEF1	464	wheat2

IPO11	181	seashell1
IQCE	203	skyblue1
IQCK	158	goldenrod2
IQGAP1	306	lemonchiffon4
IQGAP2	71	mediumpurple1
IRAK3	203	skyblue1
IRF4	464	wheat2
IRF6	278	darkgoldenrod4
IRF8	464	wheat2
IRGQ	306	lemonchiffon4
IRS2	203	skyblue1
ISG20L2	306	lemonchiffon4
ISL1	203	skyblue1
ISLR	243	darkolivegreen2
ISM1	243	darkolivegreen2
ISYNA1	203	skyblue1
ITGA11	243	darkolivegreen2
ITGA2B	205	chocolate4
ITGA4	464	wheat2
ITGA5	243	darkolivegreen2
ITGA6	158	goldenrod2
ITGAL	464	wheat2
ITGAM	203	skyblue1
ITGAX	71	mediumpurple1
ITGB2	71	mediumpurple1
ITGB3	243	darkolivegreen2
ITGB7	464	wheat2
ITGBL1	243	darkolivegreen2
ITK	464	wheat2
ITM2A	464	wheat2
ITM2C	464	wheat2
ITPKA	278	darkgoldenrod4
ITPKB	278	darkgoldenrod4
ITPKC	65	thistle4
ITPR1	464	wheat2
ITPR2	278	darkgoldenrod4
IVL	64	lightsalmon2
IWS1	205	chocolate4
JAK1	181	seashell1
JAK2	464	wheat2
JAK3	464	wheat2
JAKMIP1	464	wheat2
JAM3	243	darkolivegreen2
JDP2	205	chocolate4
JMJD6	306	lemonchiffon4
JMY	165	cornflowerblue

JPH2	81	darkseagreen4
JRKL	165	cornflowerblue
KBTBD4	306	lemonchiffon4
KBTBD8	464	wheat2
KCNA2	203	skyblue1
KCNB2	203	skyblue1
KCNC4	203	skyblue1
KCNE3	306	lemonchiffon4
KCNG1	205	chocolate4
KCNJ2	165	cornflowerblue
KCNJ8	243	darkolivegreen2
KCTD18	158	goldenrod2
KCTD20	158	goldenrod2
KCTD7	464	wheat2
KDM3A	278	darkgoldenrod4
KDM3B	205	chocolate4
KDM4A	205	chocolate4
KDM5A	165	cornflowerblue
KDM5B	203	skyblue1
KEL	203	skyblue1
KHK	165	cornflowerblue
KIAA0040	464	wheat2
KIAA0100	158	goldenrod2
KIAA0355	306	lemonchiffon4
KIAA1109	278	darkgoldenrod4
KIAA1217	278	darkgoldenrod4
KIAA1324L	165	cornflowerblue
KIAA1671	278	darkgoldenrod4
KIAA1755	243	darkolivegreen2
KIAA1958	306	lemonchiffon4
KIF16B	158	goldenrod2
KIF21B	464	wheat2
KIF26B	243	darkolivegreen2
KIF2A	181	seashell1
KIF5B	181	seashell1
KIF9	165	cornflowerblue
KL	203	skyblue1
KLF11	278	darkgoldenrod4
KLF12	278	darkgoldenrod4
KLF16	38	mistyrose
KLF2	464	wheat2
KLHL11	205	chocolate4
KLHL12	181	seashell1
KLHL20	306	lemonchiffon4
KLHL35	306	lemonchiffon4
KLHL6	464	wheat2

KLHL7	158	goldenrod2
KLK1	205	chocolate4
KLK10	64	lightsalmon2
KLK11	64	lightsalmon2
KLK12	64	lightsalmon2
KLK6	64	lightsalmon2
KLK7	64	lightsalmon2
KLK8	64	lightsalmon2
KLRB1	464	wheat2
KLRK1	464	wheat2
KMO	464	wheat2
KPNA3	181	seashell1
KPNA4	33	lightpink1
KPRP	64	lightsalmon2
KREMEN2	203	skyblue1
KRT13	27	red
KRT17	464	wheat2
KRT2	203	skyblue1
KRT23	64	lightsalmon2
KRT4	64	lightsalmon2
KRT78	64	lightsalmon2
KRT84	203	skyblue1
KRTDAP	64	lightsalmon2
L3MBTL2	205	chocolate4
L3MBTL3	278	darkgoldenrod4
L3MBTL4	203	skyblue1
LAD1	65	thistle4
LAG3	464	wheat2
LAMA4	243	darkolivegreen2
LAMB1	243	darkolivegreen2
LAMB2	243	darkolivegreen2
LANCL3	165	cornflowerblue
LAPTM5	71	mediumpurple1
LARP1	158	goldenrod2
LARP4B	165	cornflowerblue
LAS1L	165	cornflowerblue
LAT2	464	wheat2
LAX1	464	wheat2
LBH	464	wheat2
LCA5	278	darkgoldenrod4
LCK	464	wheat2
LCMT2	205	chocolate4
LCN2	27	red
LCP1	464	wheat2
LCP2	464	wheat2
LDB3	81	darkseagreen4

LEF1	464	wheat2
LGI2	203	skyblue1
LGR4	278	darkgoldenrod4
LIAS	306	lemonchiffon4
LIFR	203	skyblue1
LILRA4	464	wheat2
LILRB1	71	mediumpurple1
LIMCH1	81	darkseagreen4
LIMD2	464	wheat2
LIMK2	464	wheat2
LIN52	306	lemonchiffon4
LIN7B	205	chocolate4
LINGO1	243	darkolivegreen2
LIPH	65	thistle4
LLPH	306	lemonchiffon4
LMAN2L	181	seashell1
LMBR1L	205	chocolate4
LMCD1	243	darkolivegreen2
LMO2	464	wheat2
LMOD2	81	darkseagreen4
LNPEP	278	darkgoldenrod4
LOX	158	goldenrod2
LOXL2	243	darkolivegreen2
LPAR6	464	wheat2
LPCAT1	71	mediumpurple1
LPXN	464	wheat2
LRIG2	158	goldenrod2
LRIG3	203	skyblue1
LRMP	278	darkgoldenrod4
LRP5	278	darkgoldenrod4
LRPPRC	181	seashell1
LRRC15	243	darkolivegreen2
LRRC27	306	lemonchiffon4
LRRC3	306	lemonchiffon4
LRRC32	243	darkolivegreen2
LRRC49	203	skyblue1
LRRC57	306	lemonchiffon4
LRRC8C	278	darkgoldenrod4
LRRC8D	165	cornflowerblue
LRRK2	278	darkgoldenrod4
LSG1	38	springgreen4
LSM10	158	goldenrod2
LSM11	306	lemonchiffon4
LSP1	464	wheat2
LTA	464	wheat2
LTB	464	wheat2

LTBP2	243	darkolivegreen2
LUM	243	darkolivegreen2
LY6G6C	64	lightsalmon2
LY86	464	wheat2
LY9	464	wheat2
LYPD2	64	lightsalmon2
LYPD3	27	red
LYPLAL1	165	cornflowerblue
LYRM2	306	lemonchiffon4
LYRM4	306	lemonchiffon4
LYRM7	306	lemonchiffon4
LYST	278	darkgoldenrod4
MACC1	65	thistle4
MAFB	203	skyblue1
MAGEF1	17	aquamarine2
MAGI3	165	cornflowerblue
MAGIX	181	seashell1
MAL	64	lightsalmon2
MALL	27	red
MAMSTR	306	lemonchiffon4
MAN1A1	278	darkgoldenrod4
MAN1C1	464	wheat2
MAN2B1	464	wheat2
MANBA	71	mediumpurple1
MAP1B	243	darkolivegreen2
MAP2K1	306	lemonchiffon4
MAP3K1	278	darkgoldenrod4
MAP3K3	464	wheat2
MAP4K1	464	wheat2
MAP7	278	darkgoldenrod4
1-Mar	464	wheat2
3-Mar	158	goldenrod2
6-Mar	165	cornflowerblue
8-Mar	278	darkgoldenrod4
MARVELD1	243	darkolivegreen2
MAST3	464	wheat2
MAT2B	181	seashell1
MATK	464	wheat2
MB	81	darkseagreen4
MBD3	38	mistyrose
MBD4	17	palevioletred1
MBNL2	203	skyblue1
MBP	278	darkgoldenrod4
MBTD1	158	goldenrod2
MCAM	243	darkolivegreen2
MCCC1	17	aquamarine2

MCCC2	181	seashell1
MCF2L	203	skyblue1
MCM3AP	464	wheat2
MCOLN2	464	wheat2
MCTP2	158	goldenrod2
MDFIC	278	darkgoldenrod4
MDH1	181	seashell1
MDK	278	darkgoldenrod4
MDM2	165	cornflowerblue
MECOM	306	lemonchiffon4
MED1	165	cornflowerblue
MED12	158	goldenrod2
MED16	38	mistyrose
MED27	158	goldenrod2
MED8	181	seashell1
MEGF10	203	skyblue1
MEIS3	243	darkolivegreen2
MEOX1	464	wheat2
METAP2	181	seashell1
METTL14	158	goldenrod2
METTL2B	306	lemonchiffon4
MEX3A	71	mediumpurple1
MFAP2	243	darkolivegreen2
MFN1	38	springgreen4
MFNG	464	wheat2
MFSD6	205	chocolate4
MGA	278	darkgoldenrod4
MGAT2	205	chocolate4
MGAT3	203	skyblue1
MGAT4A	464	wheat2
MGAT4B	278	darkgoldenrod4
MICAL1	464	wheat2
MICAL2	243	darkolivegreen2
MICAL3	306	lemonchiffon4
MICALL2	203	skyblue1
MID1	278	darkgoldenrod4
MIS12	158	goldenrod2
MKKS	165	cornflowerblue
MLKL	71	mediumpurple1
MLXIP	181	seashell1
MMAB	306	lemonchiffon4
MME	243	darkolivegreen2
MMP1	243	darkolivegreen2
MMP11	243	darkolivegreen2
MMP13	243	darkolivegreen2
MMP14	243	darkolivegreen2

MMP19	203	skyblue1
MMP2	243	darkolivegreen2
MMP25	71	mediumpurple1
MMP28	203	skyblue1
MMP3	243	darkolivegreen2
MMP9	71	mediumpurple1
MNT	205	chocolate4
MORF4L1	306	lemonchiffon4
MOXD1	203	skyblue1
MPEG1	464	wheat2
MPST	205	chocolate4
MPV17	306	lemonchiffon4
MPZL2	27	red
MRC2	243	darkolivegreen2
MRFAP1	306	lemonchiffon4
MRPL16	306	lemonchiffon4
MRPL3	33	lightpink1
MRPL33	205	chocolate4
MRPL36	205	chocolate4
MRPL47	17	aquamarine2
MRPL51	181	seashell1
MRPL52	306	lemonchiffon4
MRPS21	165	cornflowerblue
MRPS31	278	darkgoldenrod4
MRPS36	306	lemonchiffon4
MRPS9	205	chocolate4
MS4A1	464	wheat2
MSI2	205	chocolate4
MSL1	158	goldenrod2
MSL2	33	lightpink1
MSL3	464	wheat2
MT1F	205	chocolate4
MTCH2	181	seashell1
MTM1	306	lemonchiffon4
MTOR	158	goldenrod2
MTX3	158	goldenrod2
MUC13	306	lemonchiffon4
MUC16	165	cornflowerblue
MUC20	65	thistle4
MUC21	64	lightsalmon2
MUM1L1	81	darkseagreen4
MUT	181	seashell1
MXD1	27	red
MXI1	278	darkgoldenrod4
MXRA5	243	darkolivegreen2
MXRA8	243	darkolivegreen2

MYADM	243	darkolivegreen2
MYADML2	81	darkseagreen4
MYBPC2	81	darkseagreen4
MYBPH	81	darkseagreen4
MYCBP2	278	darkgoldenrod4
MYCT1	243	darkolivegreen2
MYF6	81	darkseagreen4
MYH1	81	darkseagreen4
MYH2	81	darkseagreen4
MYH4	81	darkseagreen4
MYH6	81	darkseagreen4
MYH7	81	darkseagreen4
MYH8	81	darkseagreen4
MYL1	81	darkseagreen4
MYL12B	181	seashell1
MYL2	81	darkseagreen4
MYL6B	278	darkgoldenrod4
MYL9	243	darkolivegreen2
MYLK	243	darkolivegreen2
MYO18B	81	darkseagreen4
MYO1D	306	lemonchiffon4
MYO1F	464	wheat2
MYO7A	71	mediumpurple1
MYOM3	81	darkseagreen4
MYOZ1	81	darkseagreen4
MYPN	81	darkseagreen4
NAA10	165	cornflowerblue
NAA20	205	chocolate4
NAA25	158	goldenrod2
NAA40	306	lemonchiffon4
NAA50	33	lightpink1
NAB2	278	darkgoldenrod4
NACA	82	hotpink2
NAGK	306	lemonchiffon4
NAGPA	306	lemonchiffon4
NAGS	165	cornflowerblue
NAMPT	181	seashell1
NANOS1	306	lemonchiffon4
NARS	181	seashell1
NAV2	203	skyblue1
NBAS	306	lemonchiffon4
NBEAL1	278	darkgoldenrod4
NBPF1	158	goldenrod2
NBPF3	158	goldenrod2
NBPF9	165	cornflowerblue
NCAM1	81	darkseagreen4

NCBP2	38	springgreen4
NCCRP1	64	lightsalmon2
NCF1	464	wheat2
NCF4	464	wheat2
NCKAP1L	464	wheat2
NCLN	38	mistyrose
NCOA3	278	darkgoldenrod4
NCOA4	306	lemonchiffon4
NCOA5	158	goldenrod2
NCOA7	158	goldenrod2
NCS1	464	wheat2
NDFIP2	306	lemonchiffon4
NDST2	464	wheat2
NDUFA1	205	chocolate4
NDUFA10	306	lemonchiffon4
NDUFA4	181	seashell1
NDUFA6	181	seashell1
NDUFAF3	5	lightblue2
NDUFB3	181	seashell1
NDUFB5	17	aquamarine2
NDUFC2	306	lemonchiffon4
NDUFS5	181	seashell1
NDUFV3	306	lemonchiffon4
NEB	81	darkseagreen4
NEDD1	203	skyblue1
NEIL1	181	seashell1
NEK1	165	cornflowerblue
NEK6	71	mediumpurple1
NEK7	181	seashell1
NEXN	81	darkseagreen4
NFATC2	278	darkgoldenrod4
NFATC4	243	darkolivegreen2
NFE2L3	203	skyblue1
NFIC	278	darkgoldenrod4
NFIX	278	darkgoldenrod4
NFKB2	203	skyblue1
NGFR	203	skyblue1
NHSL1	278	darkgoldenrod4
NID1	243	darkolivegreen2
NID2	243	darkolivegreen2
NIPA1	165	cornflowerblue
NIPAL3	205	chocolate4
NIPSNAP1	464	wheat2
NIT1	306	lemonchiffon4
NKPD1	306	lemonchiffon4
NKX2.3	203	skyblue1

NKX2.4	165	cornflowerblue
NLN	306	lemonchiffon4
NLRC3	464	wheat2
NMD3	33	lightpink1
NMT1	158	goldenrod2
NNMT	243	darkolivegreen2
NOL10	181	seashell1
NOL12	306	lemonchiffon4
NOL9	306	lemonchiffon4
NOM1	306	lemonchiffon4
NOMO2	306	lemonchiffon4
NOP58	205	chocolate4
NOS1AP	306	lemonchiffon4
NOX4	243	darkolivegreen2
NPAT	278	darkgoldenrod4
NPM1	82	hotpink2
NPTN	306	lemonchiffon4
NQO2	205	chocolate4
NR1H3	71	mediumpurple1
NRAP	81	darkseagreen4
NRAS	181	seashell1
NRG1	165	cornflowerblue
NRM	278	darkgoldenrod4
NRP1	243	darkolivegreen2
NRSN2	278	darkgoldenrod4
NRXN3	306	lemonchiffon4
NSD1	205	chocolate4
NSUN4	306	lemonchiffon4
NT5E	243	darkolivegreen2
NTF4	278	darkgoldenrod4
NTN4	205	chocolate4
NUDT11	65	thistle4
NUDT2	158	goldenrod2
NUFIP1	205	chocolate4
NUP133	181	seashell1
NUP153	181	seashell1
NUP160	306	lemonchiffon4
NUP205	181	seashell1
NUP98	181	seashell1
NWD1	306	lemonchiffon4
OAT	306	lemonchiffon4
OCA2	203	skyblue1
OCEL1	158	goldenrod2
OGFOD2	181	seashell1
OGG1	205	chocolate4
OLFM1	203	skyblue1

OLFML2B	243	darkolivegreen2
OLFML3	243	darkolivegreen2
ONECUT2	205	chocolate4
OPA1	38	springgreen4
OR2A4	205	chocolate4
OSBP	181	seashell1
OSBPL11	33	lightpink1
OTUD7B	278	darkgoldenrod4
OVOL1	65	thistle4
P2RX1	464	wheat2
P2RX5	464	wheat2
P2RY10	464	wheat2
P2RY11	38	mistyrose
P2RY13	464	wheat2
P2RY14	464	wheat2
P2RY2	65	thistle4
P2RY8	464	wheat2
P4HA2	464	wheat2
P4HA3	243	darkolivegreen2
PACSIN1	464	wheat2
PADI1	9	slateblue4
PADI2	81	darkseagreen4
PAG1	464	wheat2
PAICS	278	darkgoldenrod4
PAK2	38	springgreen4
PAQR8	464	wheat2
PARD6A	158	goldenrod2
PARL	17	aquamarine2
PARM1	203	skyblue1
PARP4	306	lemonchiffon4
PARP8	278	darkgoldenrod4
PARVG	464	wheat2
PAWR	278	darkgoldenrod4
PAX5	464	wheat2
PAXIP1	158	goldenrod2
PCDH18	243	darkolivegreen2
PCDHB2	165	cornflowerblue
PCF11	278	darkgoldenrod4
PCGF2	464	wheat2
PCGF5	278	darkgoldenrod4
PCIF1	158	goldenrod2
PCNA	181	seashell1
PCYT1A	38	springgreen4
PDCD1	464	wheat2
PDCD10	17	aquamarine2
PDCD1LG2	71	mediumpurple1

PDE3B	278	darkgoldenrod4
PDE4A	243	darkolivegreen2
PDGFC	243	darkolivegreen2
PDGFRA	243	darkolivegreen2
PDIA3	306	lemonchiffon4
PDPN	203	skyblue1
PDS5B	165	cornflowerblue
PDZD11	205	chocolate4
PDZD2	203	skyblue1
PDZK1IP1	27	red
PDZRN3	243	darkolivegreen2
PECAM1	464	wheat2
PEX12	205	chocolate4
PEX26	306	lemonchiffon4
PFDN1	306	lemonchiffon4
PFKFB2	203	skyblue1
PFKFB3	278	darkgoldenrod4
PGLYRP3	27	red
PGPEP1	306	lemonchiffon4
PHACTR3	158	goldenrod2
PHACTR4	306	lemonchiffon4
PHAX	306	lemonchiffon4
PHC3	38	springgreen4
PHF12	306	lemonchiffon4
PHF19	165	cornflowerblue
PHF5A	205	chocolate4
PHLDB1	243	darkolivegreen2
PHLPP1	278	darkgoldenrod4
PI3	64	lightsalmon2
PICALM	306	lemonchiffon4
PIGC	165	cornflowerblue
PIGO	158	goldenrod2
PIK3AP1	464	wheat2
PIK3CA	38	springgreen4
PIK3CD	464	wheat2
PIK3CG	464	wheat2
PIK3IP1	464	wheat2
PIK3R4	33	lightpink1
PIK3R5	464	wheat2
PIK3R6	464	wheat2
PIM2	464	wheat2
PIP4K2A	464	wheat2
PIP4K2B	165	cornflowerblue
PITPNC1	464	wheat2
PITRM1	205	chocolate4
PIWIL4	203	skyblue1

PKDCC	203	skyblue1
PKP2	203	skyblue1
PKP4	278	darkgoldenrod4
PLA2G2D	464	wheat2
PLA2G4D	203	skyblue1
PLAA	181	seashell1
PLBD1	27	red
PLCB2	464	wheat2
PLCL1	203	skyblue1
PLCL2	464	wheat2
PLCXD2	278	darkgoldenrod4
PLD4	464	wheat2
PLEK	464	wheat2
PLEKHA2	464	wheat2
PLEKHB1	464	wheat2
PLEKHG2	306	lemonchiffon4
PLEKHG5	38	mistyrose
PLEKHG6	464	wheat2
PLEKHN1	65	thistle4
PLEKHO1	71	mediumpurple1
PLEKHO2	464	wheat2
PLIN3	27	red
PLVAP	243	darkolivegreen2
PLXDC2	243	darkolivegreen2
PLXND1	243	darkolivegreen2
PMEPA1	243	darkolivegreen2
PNKD	203	skyblue1
PNOC	464	wheat2
PNPLA7	464	wheat2
PNPT1	306	lemonchiffon4
PODN	243	darkolivegreen2
PODXL	203	skyblue1
POGK	278	darkgoldenrod4
POLH	306	lemonchiffon4
POLL	181	seashell1
POLQ	33	lightpink1
POLR2H	17	aquamarine2
POLR3A	205	chocolate4
POLRMT	38	mistyrose
POMT1	165	cornflowerblue
POMZP3	158	goldenrod2
POSTN	243	darkolivegreen2
POU2F2	464	wheat2
PPARA	306	lemonchiffon4
PPCDC	205	chocolate4
PPFIA1	165	cornflowerblue

PPFIBP1	278	darkgoldenrod4
PPFIBP2	203	skyblue1
PPIE	165	cornflowerblue
PPM1M	464	wheat2
PPME1	464	wheat2
PPOX	205	chocolate4
PPP1R12B	158	goldenrod2
PPP1R16A	38	mistyrose
PPP1R16B	464	wheat2
PPP1R2	38	springgreen4
PPP1R3A	81	darkseagreen4
PPP1R3F	181	seashell1
PPP1R7	158	goldenrod2
PPP2R5A	203	skyblue1
PPP2R5C	278	darkgoldenrod4
PPP3CC	464	wheat2
PPP3R1	181	seashell1
PPP4R4	203	skyblue1
PRDM10	165	cornflowerblue
PRDX6	181	seashell1
PRELID2	306	lemonchiffon4
PREP	205	chocolate4
PREX1	464	wheat2
PRF1	464	wheat2
PRICKLE1	243	darkolivegreen2
PRICKLE2	205	chocolate4
PRKACB	278	darkgoldenrod4
PRKCB	464	wheat2
PRKCE	165	cornflowerblue
PRKCH	278	darkgoldenrod4
PRKCI	38	springgreen4
PRODH	203	skyblue1
PROM2	65	thistle4
PRPF40A	181	seashell1
PRR11	306	lemonchiffon4
PRR13	278	darkgoldenrod4
PRR3	306	lemonchiffon4
PRR5L	203	skyblue1
PRSS12	165	cornflowerblue
PRSS22	65	thistle4
PRSS27	64	lightsalmon2
PRSS3	64	lightsalmon2
PRSS8	65	thistle4
PRUNE2	81	darkseagreen4
PSCA	64	lightsalmon2
PSD4	464	wheat2

PSMA1	181	seashell1
PSMA4	181	seashell1
PSMA5	181	seashell1
PSMA6	181	seashell1
PSMC2	181	seashell1
PSMC5	158	goldenrod2
PSMD2	38	springgreen4
PSMD5	181	seashell1
PSME4	181	seashell1
PSMF1	205	chocolate4
PSTK	205	chocolate4
PSTPIP1	464	wheat2
PTGDS	464	wheat2
PTGER3	243	darkolivegreen2
PTGER4	464	wheat2
PTGES	203	skyblue1
PTK2	278	darkgoldenrod4
PTK2B	464	wheat2
PTMS	278	darkgoldenrod4
PTP4A1	181	seashell1
PTPMT1	165	cornflowerblue
PTPN1	278	darkgoldenrod4
PTPN11	181	seashell1
PTPN14	278	darkgoldenrod4
PTPN2	306	lemonchiffon4
PTPN22	464	wheat2
PTPN6	464	wheat2
PTPN9	165	cornflowerblue
PTPRC	464	wheat2
PTPRCAP	464	wheat2
PTPRD	243	darkolivegreen2
PTPRF	278	darkgoldenrod4
PTPRJ	464	wheat2
PTPRM	203	skyblue1
PTPRO	71	mediumpurple1
PUM2	181	seashell1
PUS7	278	darkgoldenrod4
PWP1	181	seashell1
PWP2	165	cornflowerblue
PXDN	243	darkolivegreen2
PYGL	203	skyblue1
PYHIN1	464	wheat2
QDPR	203	skyblue1
QPCTL	306	lemonchiffon4
QSER1	278	darkgoldenrod4
RAB25	27	red

RAB31	243	darkolivegreen2
RAB32	205	chocolate4
RAB37	464	wheat2
RAB39B	464	wheat2
RAB3IL1	243	darkolivegreen2
RAB5B	165	cornflowerblue
RAB6A	181	seashell1
RAB7A	17	palevioletred1
RAB8B	464	wheat2
RABEPK	181	seashell1
RABGAP1L	278	darkgoldenrod4
RAC1	181	seashell1
RAC2	464	wheat2
RAD50	165	cornflowerblue
RAD9A	205	chocolate4
RAE1	158	goldenrod2
RAG1	165	cornflowerblue
RAI14	203	skyblue1
RALGAPA2	165	cornflowerblue
RALGAPB	165	cornflowerblue
RAMP1	203	skyblue1
RAMP3	464	wheat2
RAN	181	seashell1
RANBP17	203	skyblue1
RANBP6	158	goldenrod2
RANBP9	205	chocolate4
RANGAP1	278	darkgoldenrod4
RAPGEF1	464	wheat2
RAPGEF5	278	darkgoldenrod4
RAPGEF6	278	darkgoldenrod4
RAPGEFL1	65	thistle4
RAPH1	278	darkgoldenrod4
RARB	165	cornflowerblue
RARRES2	243	darkolivegreen2
RARS	306	lemonchiffon4
RASAL2	278	darkgoldenrod4
RASAL3	464	wheat2
RASGRF2	243	darkolivegreen2
RASGRP1	464	wheat2
RASGRP2	464	wheat2
RASL12	243	darkolivegreen2
RASSF2	464	wheat2
RASSF4	71	mediumpurple1
RASSF5	464	wheat2
RASSF6	158	goldenrod2
RB1	306	lemonchiffon4

RBBP5	306	lemonchiffon4
RBBP8	278	darkgoldenrod4
RBKS	165	cornflowerblue
RBL2	278	darkgoldenrod4
RBM12B	158	goldenrod2
RBM18	181	seashell1
RBM22	306	lemonchiffon4
RBM24	81	darkseagreen4
RBM7	306	lemonchiffon4
RCN3	243	darkolivegreen2
RCSD1	464	wheat2
RDH10	203	skyblue1
RECQL4	38	mistyrose
RELB	203	skyblue1
RELL1	306	lemonchiffon4
RETSAT	158	goldenrod2
REXO1	38	mistyrose
RFC4	17	aquamarine2
RFTN1	464	wheat2
RFX7	165	cornflowerblue
RGP1	165	cornflowerblue
RGS1	464	wheat2
RGS18	464	wheat2
RGS19	464	wheat2
RGS4	243	darkolivegreen2
RHBDD1	278	darkgoldenrod4
RHBDF1	278	darkgoldenrod4
RHBDL3	203	skyblue1
RHCG	27	red
RHEB	306	lemonchiffon4
RHEBL1	165	cornflowerblue
RHOBTB1	243	darkolivegreen2
RHOH	464	wheat2
RIC3	158	goldenrod2
RICTOR	158	goldenrod2
RILP	158	goldenrod2
RIMBP3	464	wheat2
RIMS2	205	chocolate4
RIN3	464	wheat2
RIPK3	71	mediumpurple1
RNASE6	71	mediumpurple1
RNASE7	64	lightsalmon2
RNASET2	278	darkgoldenrod4
RND2	306	lemonchiffon4
RNF13	33	lightpink1
RNF135	165	cornflowerblue

RNF139	181	seashell1
RNF150	203	skyblue1
RNF165	203	skyblue1
RNF166	71	mediumpurple1
RNF169	158	goldenrod2
RNF175	158	goldenrod2
RNF19A	203	skyblue1
RNF20	205	chocolate4
RNF220	306	lemonchiffon4
RNF25	158	goldenrod2
RNF31	158	goldenrod2
RNF41	306	lemonchiffon4
RNF6	165	cornflowerblue
RNFT2	205	chocolate4
ROBO1	203	skyblue1
RORA	278	darkgoldenrod4
RPF1	306	lemonchiffon4
RPL10	82	hotpink2
RPL10A	82	hotpink2
RPL11	82	hotpink2
RPL12	82	hotpink2
RPL13	82	hotpink2
RPL14	82	hotpink2
RPL15	82	hotpink2
RPL17	82	hotpink2
RPL18	82	hotpink2
RPL18A	82	hotpink2
RPL19	82	hotpink2
RPL22	82	hotpink2
RPL23	82	hotpink2
RPL23A	82	hotpink2
RPL24	82	hotpink2
RPL26	82	hotpink2
RPL27	82	hotpink2
RPL27A	82	hotpink2
RPL28	82	hotpink2
RPL29	82	hotpink2
RPL3	82	hotpink2
RPL30	82	hotpink2
RPL31	82	hotpink2
RPL32	82	hotpink2
RPL34	82	hotpink2
RPL35	82	hotpink2
RPL36	82	hotpink2
RPL37	82	hotpink2
RPL37A	82	hotpink2

RPL38	82	hotpink2
RPL39	82	hotpink2
RPL4	82	hotpink2
RPL41	82	hotpink2
RPL6	82	hotpink2
RPL7	82	hotpink2
RPL7A	82	hotpink2
RPL7L1	306	lemonchiffon4
RPL8	82	hotpink2
RPLP0	82	hotpink2
RPLP1	82	hotpink2
RPLP2	82	hotpink2
RPN1	17	palevioletred1
RPN2	306	lemonchiffon4
RPP38	158	goldenrod2
RPP40	306	lemonchiffon4
RPRD1B	165	cornflowerblue
RPRD2	165	cornflowerblue
RPS10	82	hotpink2
RPS11	82	hotpink2
RPS12	82	hotpink2
RPS13	82	hotpink2
RPS14	82	hotpink2
RPS15A	82	hotpink2
RPS16	82	hotpink2
RPS18	82	hotpink2
RPS19	82	hotpink2
RPS2	82	hotpink2
RPS20	82	hotpink2
RPS21	82	hotpink2
RPS23	82	hotpink2
RPS24	82	hotpink2
RPS25	82	hotpink2
RPS27	82	hotpink2
RPS27A	82	hotpink2
RPS3	82	hotpink2
RPS3A	82	hotpink2
RPS4X	82	hotpink2
RPS5	82	hotpink2
RPS6	82	hotpink2
RPS6KL1	306	lemonchiffon4
RPS7	82	hotpink2
RPS8	82	hotpink2
RPSA	82	hotpink2
RPUSD3	205	chocolate4
RRAGA	306	lemonchiffon4

RRAGC	306	lemonchiffon4
RRP1B	165	cornflowerblue
RSC1A1	205	chocolate4
RSF1	158	goldenrod2
RSPH3	306	lemonchiffon4
RSRC1	33	lightpink1
RSU1	205	chocolate4
RTF1	205	chocolate4
RTKN	278	darkgoldenrod4
RTN1	71	mediumpurple1
RTN4RL1	203	skyblue1
RTP3	203	skyblue1
RUFY1	158	goldenrod2
RUNDC1	306	lemonchiffon4
RUNX2	243	darkolivegreen2
RUNX3	464	wheat2
RUVBL1	17	palevioletred1
RYK	33	lightpink1
RYR1	81	darkseagreen4
RYR3	203	skyblue1
S100A12	9	slateblue4
S100A13	158	goldenrod2
S100A14	65	thistle4
S100A7	64	lightsalmon2
S100A8	27	red
S100A9	9	slateblue4
S100P	64	lightsalmon2
S1PR4	464	wheat2
S1PR5	278	darkgoldenrod4
SAA1	158	goldenrod2
SALL3	278	darkgoldenrod4
SAMD3	464	wheat2
SAMD8	158	goldenrod2
SAMHD1	278	darkgoldenrod4
SAMSN1	464	wheat2
SAP130	205	chocolate4
SARDH	464	wheat2
SARS	205	chocolate4
SART3	165	cornflowerblue
SASH3	464	wheat2
SATB1	278	darkgoldenrod4
SATB2	243	darkolivegreen2
SBSN	64	lightsalmon2
SCAF1	38	mistyrose
SCEL	9	slateblue4
SCHIP1	464	wheat2

SCML4	464	wheat2
SCN4A	81	darkseagreen4
SCN8A	165	cornflowerblue
SCNN1A	65	thistle4
SCNN1G	203	skyblue1
SCRIB	38	mistyrose
SCYL2	181	seashell1
SDAD1	181	seashell1
SDC2	243	darkolivegreen2
SDCBP	181	seashell1
SDCBP2	65	thistle4
SDHC	181	seashell1
SDR42E1	158	goldenrod2
SEC11A	306	lemonchiffon4
SEC14L5	158	goldenrod2
SEC22A	17	palevioletred1
SEC23B	306	lemonchiffon4
SEC24A	181	seashell1
SEC61A1	17	palevioletred1
SEC61B	181	seashell1
SEC61G	306	lemonchiffon4
SEC62	38	springgreen4
SEH1L	181	seashell1
SEL1L	278	darkgoldenrod4
SEL1L3	464	wheat2
SELE	243	darkolivegreen2
SELPLG	464	wheat2
SEMA3B	464	wheat2
SEMA3D	165	cornflowerblue
SEMA4C	203	skyblue1
SEMA4D	464	wheat2
SEMA6A	203	skyblue1
SEMA7A	464	wheat2
SENP2	38	springgreen4
SENP5	38	springgreen4
1-Sep	464	wheat2
6-Sep	464	wheat2
SERAC1	205	chocolate4
SERP1	17	palevioletred1
SERPINB11	64	lightsalmon2
SERPINB12	165	cornflowerblue
SERPINE1	243	darkolivegreen2
SERPINH1	243	darkolivegreen2
SET	181	seashell1
SETDB2	464	wheat2
SETX	278	darkgoldenrod4

SF3A1	306	lemonchiffon4
SF3A2	38	mistyrose
SFMBT2	278	darkgoldenrod4
SFRP2	243	darkolivegreen2
SFRP4	243	darkolivegreen2
SGCA	81	darkseagreen4
SGCD	243	darkolivegreen2
SGCG	81	darkseagreen4
SGK3	306	lemonchiffon4
SGPL1	181	seashell1
SGPP1	464	wheat2
SH2D1A	464	wheat2
SH2D3C	464	wheat2
SH3BP4	278	darkgoldenrod4
SH3BP5	203	skyblue1
SH3KBP1	464	wheat2
SH3PXD2A	278	darkgoldenrod4
SH3RF1	278	darkgoldenrod4
SH3RF3	243	darkolivegreen2
SHC2	243	darkolivegreen2
SIAH2	17	palevioletred1
SIDT1	464	wheat2
SIDT2	464	wheat2
SIGLEC14	71	mediumpurple1
SIGLEC5	71	mediumpurple1
SIK2	205	chocolate4
SIK3	278	darkgoldenrod4
SIL1	158	goldenrod2
SIM1	203	skyblue1
SIN3A	205	chocolate4
SIPA1	464	wheat2
SIRPG	464	wheat2
SIT1	464	wheat2
SKAP1	464	wheat2
SKAP2	278	darkgoldenrod4
SKIL	38	springgreen4
SLA	464	wheat2
SLA2	464	wheat2
SLAMF1	464	wheat2
SLAMF6	464	wheat2
SLAMF7	464	wheat2
SLC12A7	203	skyblue1
SLC17A9	71	mediumpurple1
SLC22A15	203	skyblue1
SLC25A10	38	mistyrose
SLC25A15	306	lemonchiffon4

SLC25A22	203	skyblue1
SLC25A24	181	seashell1
SLC25A26	158	goldenrod2
SLC25A29	181	seashell1
SLC25A36	33	lightpink1
SLC25A6	82	hotpink2
SLC26A2	165	cornflowerblue
SLC29A3	71	mediumpurple1
SLC30A1	205	chocolate4
SLC30A4	205	chocolate4
SLC30A5	165	cornflowerblue
SLC30A9	165	cornflowerblue
SLC31A1	306	lemonchiffon4
SLC35C2	181	seashell1
SLC35E1	306	lemonchiffon4
SLC35E3	306	lemonchiffon4
SLC35E4	205	chocolate4
SLC35F2	203	skyblue1
SLC38A2	181	seashell1
SLC39A1	278	darkgoldenrod4
SLC39A2	27	red
SLC45A4	203	skyblue1
SLC46A3	71	mediumpurple1
SLC4A1AP	306	lemonchiffon4
SLC4A5	306	lemonchiffon4
SLC6A14	65	thistle4
SLC7A2	203	skyblue1
SLC7A6	278	darkgoldenrod4
SLC8A1	71	mediumpurple1
SLC9A2	203	skyblue1
SLC9A3R1	65	thistle4
SLCO3A1	165	cornflowerblue
SLFN5	278	darkgoldenrod4
SLIT3	243	darkolivegreen2
SLN	81	darkseagreen4
SLU7	306	lemonchiffon4
SLURP1	64	lightsalmon2
SMAGP	65	thistle4
SMAP1	181	seashell1
SMAP2	464	wheat2
SMARCA1	278	darkgoldenrod4
SMARCC2	181	seashell1
SMC1A	278	darkgoldenrod4
SMCHD1	278	darkgoldenrod4
SMG1	158	goldenrod2
SMPD3	464	wheat2

SMUG1	306	lemonchiffon4
SMYD1	81	darkseagreen4
SMYD4	306	lemonchiffon4
SNAI1	243	darkolivegreen2
SNAI3	464	wheat2
SNAP25	203	skyblue1
SNAP29	306	lemonchiffon4
SNAPC1	306	lemonchiffon4
SNIP1	306	lemonchiffon4
SNRNP200	158	goldenrod2
SNRNP35	306	lemonchiffon4
SNRNP48	158	goldenrod2
SNRPE	205	chocolate4
SNRPG	181	seashell1
SNUPN	205	chocolate4
SNX10	71	mediumpurple1
SNX20	464	wheat2
SNX27	158	goldenrod2
SNX29	278	darkgoldenrod4
SNX33	65	thistle4
SNX4	33	lightpink1
SNX6	181	seashell1
SOAT1	306	lemonchiffon4
SOBP	243	darkolivegreen2
SOCS7	306	lemonchiffon4
SOD1	181	seashell1
SORCS2	243	darkolivegreen2
SOX14	203	skyblue1
SOX4	71	mediumpurple1
SOX8	203	skyblue1
SP140	464	wheat2
SP5	203	skyblue1
SP6	165	cornflowerblue
SPA17	205	chocolate4
SPAG9	278	darkgoldenrod4
SPARC	243	darkolivegreen2
SPATA13	278	darkgoldenrod4
SPATS2	306	lemonchiffon4
SPG11	278	darkgoldenrod4
SPHK1	278	darkgoldenrod4
SPIN4	165	cornflowerblue
SPINK5	64	lightsalmon2
SPINK7	64	lightsalmon2
SPNS2	64	lightsalmon2
SPOCK1	243	darkolivegreen2
SPOCK2	464	wheat2

SPON1	243	darkolivegreen2
SPPL2B	38	mistyrose
SPRR1A	64	lightsalmon2
SPRR1B	64	lightsalmon2
SPRR2A	64	lightsalmon2
SPRR2B	64	lightsalmon2
SPRR2D	64	lightsalmon2
SPRR2E	64	lightsalmon2
SPRR2F	64	lightsalmon2
SPRR2G	64	lightsalmon2
SPRR3	64	lightsalmon2
SPRYD4	306	lemonchiffon4
SRD5A3	65	thistle4
SRGAP1	158	goldenrod2
SRP14	181	seashell1
SRP19	306	lemonchiffon4
SRP72	205	chocolate4
SRR	165	cornflowerblue
SRRM1	158	goldenrod2
SSBP2	306	lemonchiffon4
SSC5D	243	darkolivegreen2
SSH2	278	darkgoldenrod4
SSH3	65	thistle4
SSU72	205	chocolate4
SSX2IP	278	darkgoldenrod4
ST3GAL5	464	wheat2
ST6GAL1	464	wheat2
ST6GALNAC2	464	wheat2
ST8SIA4	464	wheat2
STAM	306	lemonchiffon4
STAMBPL1	464	wheat2
STAP1	464	wheat2
STARD13	243	darkolivegreen2
STAT4	464	wheat2
STAT5A	464	wheat2
STAU1	205	chocolate4
STC1	278	darkgoldenrod4
STC2	464	wheat2
STIM2	203	skyblue1
STK10	464	wheat2
STK11	38	mistyrose
STK16	165	cornflowerblue
STK17B	464	wheat2
STK3	278	darkgoldenrod4
STK32C	165	cornflowerblue
STK39	306	lemonchiffon4

STK4	464	wheat2
STRN3	205	chocolate4
STX11	71	mediumpurple1
STX17	158	goldenrod2
STX18	158	goldenrod2
STX3	464	wheat2
STX7	278	darkgoldenrod4
STXBP1	203	skyblue1
STXBP4	158	goldenrod2
STXBP6	203	skyblue1
STYXL1	158	goldenrod2
SUCLA2	205	chocolate4
SUDS3	158	goldenrod2
SULF2	243	darkolivegreen2
SULT1E1	203	skyblue1
SUMO1	181	seashell1
SUMO2	181	seashell1
SUN1	278	darkgoldenrod4
SUPT16H	205	chocolate4
SUSD3	464	wheat2
SWAP70	278	darkgoldenrod4
SYDE2	306	lemonchiffon4
SYK	464	wheat2
SYNPO2	203	skyblue1
SYNRG	278	darkgoldenrod4
SYPL1	181	seashell1
SYPL2	81	darkseagreen4
SYT11	464	wheat2
SYT8	205	chocolate4
SYVN1	464	wheat2
TACSTD2	65	thistle4
TAF12	165	cornflowerblue
TAF1B	205	chocolate4
TAF1L	306	lemonchiffon4
TAGAP	464	wheat2
TAGLN	243	darkolivegreen2
TANC1	278	darkgoldenrod4
TANC2	278	darkgoldenrod4
TAOK3	278	darkgoldenrod4
TBC1D10B	38	mistyrose
TBC1D14	158	goldenrod2
TBC1D16	243	darkolivegreen2
TBC1D23	33	lightpink1
TBC1D2B	278	darkgoldenrod4
TBK1	181	seashell1
TBL1XR1	38	springgreen4

TBX18	205	chocolate4
TBX21	464	wheat2
TCAP	81	darkseagreen4
TCEAL3	165	cornflowerblue
TCERG1	278	darkgoldenrod4
TCF20	278	darkgoldenrod4
TCF7	464	wheat2
TCF7L2	278	darkgoldenrod4
TCL1A	464	wheat2
TDRD9	165	cornflowerblue
TEAD1	278	darkgoldenrod4
TES	181	seashell1
TEX15	165	cornflowerblue
TEX261	306	lemonchiffon4
TFEC	71	mediumpurple1
TFRC	38	springgreen4
TG	203	skyblue1
TGFB3	243	darkolivegreen2
TGFBI	243	darkolivegreen2
TGFBR1	306	lemonchiffon4
TGIF1	464	wheat2
TGIF2	203	skyblue1
TGM1	64	lightsalmon2
TGM3	64	lightsalmon2
THAP1	181	seashell1
THBS1	243	darkolivegreen2
THBS2	243	darkolivegreen2
THEM5	165	cornflowerblue
THEMIS	464	wheat2
THOC5	205	chocolate4
THRAP3	205	chocolate4
THY1	243	darkolivegreen2
TICAM1	65	thistle4
TIFAB	464	wheat2
TIGD5	38	mistyrose
TIGIT	464	wheat2
TIMP1	243	darkolivegreen2
TIMP2	243	darkolivegreen2
TIMP3	243	darkolivegreen2
TJAP1	181	seashell1
TLE4	165	cornflowerblue
TLN2	243	darkolivegreen2
TLR1	203	skyblue1
TLR9	464	wheat2
TM7SF3	203	skyblue1
TM9SF2	181	seashell1

TMBIM4	278	darkgoldenrod4
TMBIM6	181	seashell1
TMC7	306	lemonchiffon4
TMC8	464	wheat2
TMCC1	33	lightpink1
TMED2	181	seashell1
TMED7	181	seashell1
TMEM101	158	goldenrod2
TMEM107	205	chocolate4
TMEM120B	306	lemonchiffon4
TMEM126A	205	chocolate4
TMEM132A	278	darkgoldenrod4
TMEM14B	306	lemonchiffon4
TMEM150C	203	skyblue1
TMEM163	464	wheat2
TMEM173	203	skyblue1
TMEM176A	71	mediumpurple1
TMEM184A	65	thistle4
TMEM185A	158	goldenrod2
TMEM192	306	lemonchiffon4
TMEM219	158	goldenrod2
TMEM223	165	cornflowerblue
TMEM39B	158	goldenrod2
TMEM41A	17	aquamarine2
TMEM43	205	chocolate4
TMEM44	17	aquamarine2
TMEM45B	64	lightsalmon2
TMEM52	165	cornflowerblue
TMEM59	181	seashell1
TMEM60	205	chocolate4
TMEM79	65	thistle4
TMEM87A	181	seashell1
TMEM91	205	chocolate4
TMEM99	158	goldenrod2
TMEM9B	306	lemonchiffon4
TMPRSS11A	27	red
TMPRSS11B	64	lightsalmon2
TMPRSS11D	27	red
TMSB15B	306	lemonchiffon4
TMUB2	205	chocolate4
TMX2	181	seashell1
TNC	243	darkolivegreen2
TNFAIP6	243	darkolivegreen2
TNFAIP8L1	306	lemonchiffon4
TNFAIP8L2	464	wheat2
TNFRSF13B	464	wheat2

TNFRSF17	464	wheat2
TNFRSF1B	464	wheat2
TNFRSF8	464	wheat2
TNFSF11	464	wheat2
TNFSF8	464	wheat2
TNFSF9	165	cornflowerblue
TNKS1BP1	464	wheat2
TNNC1	81	darkseagreen4
TNNC2	81	darkseagreen4
TNNI2	81	darkseagreen4
TNNI3	165	cornflowerblue
TNNT1	81	darkseagreen4
TNNT3	81	darkseagreen4
TNS3	243	darkolivegreen2
TNS4	278	darkgoldenrod4
TOB2	158	goldenrod2
TOE1	158	goldenrod2
TOMM22	181	seashell1
TOP1	205	chocolate4
TOP3A	306	lemonchiffon4
TOPBP1	33	lightpink1
TOPORS	205	chocolate4
TOR1AIP2	306	lemonchiffon4
TOR3A	205	chocolate4
TOX	464	wheat2
TOX2	464	wheat2
TP53I11	203	skyblue1
TP53INP1	464	wheat2
TPBG	464	wheat2
TPD52L1	203	skyblue1
TPK1	71	mediumpurple1
TPM1	243	darkolivegreen2
TPR	165	cornflowerblue
TPRG1L	306	lemonchiffon4
TPT1	82	hotpink2
TRA2B	17	aquamarine2
TRAF2	203	skyblue1
TRAF3	464	wheat2
TRAF3IP3	464	wheat2
TRAF5	464	wheat2
TRANK1	464	wheat2
TRAPPC10	278	darkgoldenrod4
TRAT1	464	wheat2
TRDN	81	darkseagreen4
TRIB3	165	cornflowerblue
TRIM16	65	thistle4

TRIM29	65	thistle4
TRIM36	165	cornflowerblue
TRIM4	278	darkgoldenrod4
TRIM54	81	darkseagreen4
TRIM56	181	seashell1
TRIM63	81	darkseagreen4
TRIM68	306	lemonchiffon4
TRIM8	203	skyblue1
TRIP12	165	cornflowerblue
TRNAU1AP	205	chocolate4
TRNP1	27	red
TRO	243	darkolivegreen2
TRPM2	71	mediumpurple1
TRPV2	71	mediumpurple1
TRPV6	203	skyblue1
TRRAP	278	darkgoldenrod4
TSC22D1	203	skyblue1
TSC22D3	278	darkgoldenrod4
TSEN54	38	mistyrose
TSFM	205	chocolate4
TSG101	306	lemonchiffon4
TSPAN17	203	skyblue1
TSPAN2	243	darkolivegreen2
TSPAN31	306	lemonchiffon4
TSPAN33	464	wheat2
TSR2	306	lemonchiffon4
TTC1	205	chocolate4
TTC13	278	darkgoldenrod4
TTC22	65	thistle4
TTC37	181	seashell1
TTC39B	165	cornflowerblue
TTC5	158	goldenrod2
TTC9	65	thistle4
TTLL4	203	skyblue1
TTN	81	darkseagreen4
TTYH2	71	mediumpurple1
TUBG2	278	darkgoldenrod4
TWIST1	243	darkolivegreen2
TWIST2	243	darkolivegreen2
TWISTNB	181	seashell1
TXN	181	seashell1
TXNDC11	464	wheat2
TXNDC5	464	wheat2
TXNDC9	181	seashell1
TXNIP	464	wheat2
TXNRD2	205	chocolate4

TYK2	203	skyblue1
TYW1	205	chocolate4
TYW3	158	goldenrod2
UACA	278	darkgoldenrod4
UBA5	17	palevioletred1
UBA52	82	hotpink2
UBA7	464	wheat2
UBAP2	205	chocolate4
UBASH3A	464	wheat2
UBASH3B	464	wheat2
UBD	203	skyblue1
UBE2D2	205	chocolate4
UBE2F	158	goldenrod2
UBE2G1	181	seashell1
UBE2J1	464	wheat2
UBE3C	205	chocolate4
UBE4A	278	darkgoldenrod4
UBOX5	306	lemonchiffon4
UBQLN1	181	seashell1
UBQLN2	306	lemonchiffon4
UBQLN4	278	darkgoldenrod4
UBTD1	243	darkolivegreen2
UCHL3	181	seashell1
UCK2	464	wheat2
UCN2	278	darkgoldenrod4
UCP2	464	wheat2
UGCG	181	seashell1
ULBP1	464	wheat2
ULK4	464	wheat2
UMPS	17	palevioletred1
UNC119B	278	darkgoldenrod4
UNC13D	71	mediumpurple1
UNC45B	81	darkseagreen4
UPF2	306	lemonchiffon4
UQCRFS1	306	lemonchiffon4
USF1	158	goldenrod2
USP15	278	darkgoldenrod4
USP30	278	darkgoldenrod4
USP31	278	darkgoldenrod4
USP32	205	chocolate4
USP35	165	cornflowerblue
USP38	181	seashell1
USP40	165	cornflowerblue
USP48	278	darkgoldenrod4
USP53	158	goldenrod2
USP6	306	lemonchiffon4

USP8	205	chocolate4
USP9Y	165	cornflowerblue
UST	158	goldenrod2
UTP20	181	seashell1
UTP3	306	lemonchiffon4
UTP6	205	chocolate4
UTRN	278	darkgoldenrod4
VANGL1	278	darkgoldenrod4
VAV1	464	wheat2
VAV2	203	skyblue1
VCAM1	203	skyblue1
VCAN	243	darkolivegreen2
VEZF1	278	darkgoldenrod4
VEZT	158	goldenrod2
VNN2	464	wheat2
VOPP1	71	mediumpurple1
VPREB3	464	wheat2
VPS13C	278	darkgoldenrod4
VPS33B	205	chocolate4
VPS36	158	goldenrod2
VPS37B	65	thistle4
VPS53	306	lemonchiffon4
VPS72	158	goldenrod2
VPS8	38	springgreen4
VRK2	203	skyblue1
VSIG10L	64	lightsalmon2
VSTM2L	203	skyblue1
VTI1A	306	lemonchiffon4
VWA2	278	darkgoldenrod4
WAS	464	wheat2
WASL	306	lemonchiffon4
WDFY4	464	wheat2
WDR12	306	lemonchiffon4
WDR31	306	lemonchiffon4
WDR36	181	seashell1
WDR37	278	darkgoldenrod4
WDR45	306	lemonchiffon4
WDR54	306	lemonchiffon4
WDR83	205	chocolate4
WDR92	306	lemonchiffon4
WDTC1	165	cornflowerblue
WDYHV1	158	goldenrod2
WEE1	278	darkgoldenrod4
WIPF1	464	wheat2
WIPI2	158	goldenrod2
WNK2	203	skyblue1

WNT10A	203	skyblue1
WNT2	243	darkolivegreen2
WSCD1	203	skyblue1
WTIP	158	goldenrod2
XBP1	464	wheat2
XDH	65	thistle4
XIRP1	81	darkseagreen4
XIRP2	81	darkseagreen4
XKR4	203	skyblue1
XPNPEP3	306	lemonchiffon4
XPO6	278	darkgoldenrod4
XPR1	278	darkgoldenrod4
XRCC5	181	seashell1
XRCC6	306	lemonchiffon4
XRN1	33	lightpink1
XYLB	306	lemonchiffon4
YAP1	278	darkgoldenrod4
YEATS2	38	springgreen4
YIPF5	205	chocolate4
YME1L1	181	seashell1
YTHDC2	278	darkgoldenrod4
YWHAE	306	lemonchiffon4
YY1AP1	165	cornflowerblue
ZADH2	158	goldenrod2
ZAP70	464	wheat2
ZBED2	278	darkgoldenrod4
ZBED4	278	darkgoldenrod4
ZBP1	464	wheat2
ZBTB2	306	lemonchiffon4
ZBTB38	278	darkgoldenrod4
ZBTB4	165	cornflowerblue
ZBTB40	278	darkgoldenrod4
ZBTB46	203	skyblue1
ZBTB7A	181	seashell1
ZBTB8A	306	lemonchiffon4
ZBTB8OS	306	lemonchiffon4
ZC3H15	205	chocolate4
ZC3H3	38	mistyrose
ZC3HAV1	278	darkgoldenrod4
ZC3HAV1L	278	darkgoldenrod4
ZCCHC24	243	darkolivegreen2
ZCCHC8	306	lemonchiffon4
ZDBF2	165	cornflowerblue
ZEB1	243	darkolivegreen2
ZFAND2A	181	seashell1
ZFP3	158	goldenrod2

ZFPL1	205	chocolate4
ZFPM1	38	mistyrose
ZFYVE19	158	goldenrod2
ZFYVE28	464	wheat2
ZFYVE9	278	darkgoldenrod4
ZIC2	165	cornflowerblue
ZIC5	165	cornflowerblue
ZKSCAN1	306	lemonchiffon4
ZKSCAN2	278	darkgoldenrod4
ZKSCAN3	306	lemonchiffon4
ZKSCAN4	306	lemonchiffon4
ZKSCAN5	158	goldenrod2
ZMAT2	205	chocolate4
ZMAT5	205	chocolate4
ZMIZ2	203	skyblue1
ZMYM1	165	cornflowerblue
ZMYM2	165	cornflowerblue
ZMYM3	165	cornflowerblue
ZMYND10	205	chocolate4
ZNF100	165	cornflowerblue
ZNF131	158	goldenrod2
ZNF133	158	goldenrod2
ZNF14	464	wheat2
ZNF143	205	chocolate4
ZNF148	33	lightpink1
ZNF169	205	chocolate4
ZNF174	306	lemonchiffon4
ZNF177	165	cornflowerblue
ZNF184	306	lemonchiffon4
ZNF185	27	red
ZNF20	205	chocolate4
ZNF200	158	goldenrod2
ZNF217	165	cornflowerblue
ZNF268	158	goldenrod2
ZNF32	205	chocolate4
ZNF333	306	lemonchiffon4
ZNF341	205	chocolate4
ZNF347	306	lemonchiffon4
ZNF423	243	darkolivegreen2
ZNF426	306	lemonchiffon4
ZNF431	306	lemonchiffon4
ZNF441	464	wheat2
ZNF460	306	lemonchiffon4
ZNF462	278	darkgoldenrod4
ZNF469	243	darkolivegreen2
ZNF48	38	mistyrose

ZNF484	165	cornflowerblue
ZNF500	306	lemonchiffon4
ZNF506	464	wheat2
ZNF511	158	goldenrod2
ZNF521	243	darkolivegreen2
ZNF532	278	darkgoldenrod4
ZNF544	306	lemonchiffon4
ZNF558	205	chocolate4
ZNF567	165	cornflowerblue
ZNF576	205	chocolate4
ZNF580	181	seashell1
ZNF587	306	lemonchiffon4
ZNF592	165	cornflowerblue
ZNF598	38	mistyrose
ZNF609	165	cornflowerblue
ZNF639	38	springgreen4
ZNF649	158	goldenrod2
ZNF652	158	goldenrod2
ZNF658	165	cornflowerblue
ZNF664	278	darkgoldenrod4
ZNF683	464	wheat2
ZNF70	306	lemonchiffon4
ZNF704	278	darkgoldenrod4
ZNF708	278	darkgoldenrod4
ZNF71	158	goldenrod2
ZNF750	65	thistle4
ZNF770	181	seashell1
ZNF771	205	chocolate4
ZNF786	181	seashell1
ZNF787	38	mistyrose
ZNF8	205	chocolate4
ZNF805	306	lemonchiffon4
ZNF813	158	goldenrod2
ZNF831	464	wheat2
ZNF853	243	darkolivegreen2
ZNHIT1	205	chocolate4
ZSCAN22	306	lemonchiffon4
ZSWIM1	306	lemonchiffon4
ZSWIM6	205	chocolate4
ZSWIM7	306	lemonchiffon4
ZZEF1	278	darkgoldenrod4
